# Transcriptomic Signature Differences Between SARS-CoV-2 and Influenza Virus Infected Patients

**DOI:** 10.3389/fimmu.2021.666163

**Published:** 2021-05-31

**Authors:** Stéphanie Bibert, Nicolas Guex, Joao Lourenco, Thomas Brahier, Matthaios Papadimitriou-Olivgeris, Lauro Damonti, Oriol Manuel, Robin Liechti, Lou Götz, Jonathan Tschopp, Mathieu Quinodoz, Peter Vollenweider, Jean-Luc Pagani, Mauro Oddo, Olivier Hügli, Frédéric Lamoth, Véronique Erard, Cathy Voide, Mauro Delorenzi, Nathalie Rufer, Fabio Candotti, Carlo Rivolta, Noémie Boillat-Blanco, Pierre-Yves Bochud, the RegCOVID Study Group, Bochud Pierre-Yves

**Affiliations:** ^1^ Infectious Diseases Service, Department of Medicine, University Hospital and University of Lausanne, Lausanne, Switzerland; ^2^ Bioinformatics Competence Center, University of Lausanne, Lausanne, Switzerland; ^3^ SIB Swiss Institute of Bioinformatics and Department of Fundamenal Oncology, University of Lausanne, Lausanne, Switzerland; ^4^ Department of Infectious Diseases, Bern University Hospital, Bern, Switzerland; ^5^ Infectious Diseases Service and Transplantation Center, Department of Medicine, University Hospital and University of Lausanne, Lausanne, Switzerland; ^6^ Institute of Molecular and Clinical Ophthalmology Basel (IOB), Basel, Switzerland; ^7^ Department of Ophthalmology, University Hospital Basel, Basel, Switzerland; ^8^ Department of Genetics and Genome Biology, University of Leicester, Leicester, United Kingdom; ^9^ Internal Medicine Service, Department of Medicine, University Hospital and University of Lausanne, Lausanne, Switzerland; ^10^ Department of Adult Intensive Care Medicine, University Hospital and University of Lausanne, Lausanne, Switzerland; ^11^ Emergency Department, University Hospital and University of Lausanne, Lausanne, Switzerland; ^12^ Department of Laboratory Medicine, Institute of Microbiology, University Hospital and University of Lausanne, Lausanne, Switzerland; ^13^ Clinique de Médecine et spécialités, Infectiologie, Hôpital Fribourgeois-Fribourg, Fribourg, Switzerland; ^14^ Department of Infectious Diseases, Central Institute, Valais Hospital, Sion, Switzerland; ^15^ Department of Oncology, University Hospital and University of Lausanne, Epalinges, Switzerland; ^16^ Division of Immunology and Allergy, University Hospital and University of Lausanne, Lausanne, Switzerland

**Keywords:** COVID-19, SARS-CoV-2, influenza, whole blood transcriptome, RNA-sequencing, immune profiling

## Abstract

The reason why most individuals with COVID-19 have relatively limited symptoms while other develop respiratory distress with life-threatening complications remains unknown. Increasing evidence suggests that COVID-19 associated adverse outcomes mainly rely on dysregulated immunity. Here, we compared transcriptomic profiles of blood cells from 103 patients with different severity levels of COVID-19 with that of 27 healthy and 22 influenza-infected individuals. Data provided a complete overview of SARS-CoV-2-induced immune signature, including a dramatic defect in IFN responses, a reduction of toxicity-related molecules in NK cells, an increased degranulation of neutrophils, a dysregulation of T cells, a dramatic increase in B cell function and immunoglobulin production, as well as an important over-expression of genes involved in metabolism and cell cycle in patients infected with SARS-CoV-2 compared to those infected with influenza viruses. These features also differed according to COVID-19 severity. Overall and specific gene expression patterns across groups can be visualized on an interactive website (https://bix.unil.ch/covid/). Collectively, these transcriptomic host responses to SARS-CoV-2 infection are discussed in the context of current studies, thereby improving our understanding of COVID-19 pathogenesis and shaping the severity level of COVID-19.

## Introduction

Coronaviruses (CoV) are enveloped single-stranded positive-sense RNA viruses surrounded by spike glycoproteins shaping the typical “corona-like” appearance (1). To date, seven strains of human CoV have been identified. Four of them (HCoV-229E, HCoV-OC43, HCoV-NL63, HKU1) circulate in the population and cause only mild upper-respiratory tract infections in immunocompetent individuals (2). In the last two decades, three highly pathogenic viruses acquired by zoonotic transmission caused outbreaks of severe pneumonia. Severe acute respiratory virus (SARS)-related CoV 1 infected ~8000 individuals in 2002-3, with a fatality rate of 10%; the Middle-East respiratory syndrome CoV (MERS-CoV) infected ~8000 individuals since 2012, with a ~36% fatality rate; the new coronavirus SARS-CoV-2 emerged in the province of Wuhan (China) in the end of 2019, infecting more than 100,200,000 individuals worldwide and killing over 2’150’000 as of January 2021.

While the immune response to SARS-CoV-2 has not yet been fully characterized, it is likely to engage immune mechanisms similar to those previously described for SARS-CoV and MERS-CoV, and, more generally, other RNA viruses (3, 4). Viral single stranded and double-stranded RNA are recognized by at least 3 families of pattern recognition receptors (PRR), including extracellular and endosomal Toll-like receptors (TLR3/4/7/8), cytoplasmic retinoic acid-inducible gene I-like receptors (RIG-I/MDA5), and the cytosolic RNA-activated protein kinase (PKR). Signal transduction by PRR subsequently leads to the induction of transcription factors such as interferon regulatory factors (IRFs) and nuclear factor κ B (NF-κ B), thereby inducing the synthesis and secretion of pro-inflammatory cytokines such as Type I and III interferons (IFNs), as well as the production of chemokines inducing adaptive immunity. In turn, both type I and III IFNs induce the expression of interferon stimulated genes (ISGs), which restrict and limit viral spread and stimulate the adaptive immune responses (5, 6), resulting in the generation of viral peptide-specific T cells (7, 8) and the production of viral-specific antibodies (9–11).

Increasing evidences suggest that SARS-CoV-2 induces specific response mechanisms, probably distinct from those triggered by other viruses, that can lead to major immune dysregulation (12). However, only few clinical studies have investigated the comparison of immune activation responses during COVID-19 to other viral infections. To better understand the specific features of the immune response to COVID-19, we compared the transcriptional profiles of patients infected by SARS-CoV-2 across different disease severity to that of patients infected by Influenza A or B viruses. Altogether, our results suggest that severe COVID-19 presentation results from a defect in or escape from innate immunity associated with an unbalanced adaptive immune response.

## Methods

### Patients

This prospective observational study of SARS-CoV2 and Influenza viruses-infected patients was conducted in the emergency department, internal medical ward and in the intensive care unit of Lausanne University Hospital (CHUV), a tertiary care center in Switzerland. Adult patients were included in this study if COVID-19 or Influenza were confirmed by real time polymerase chain reaction from a nasopharyngeal swab. COVID-19 patients were included between February 6^th^ 2020 and April 3^rd^ 2020. Patients with Influenza were included between January 21^st^ 2015 and March 17th 2020. Healthy volunteers had a negative COVID-19 serology and were included in June 2020. Patients and healthy volunteers included in this study signed an informed consent form for genetic and functional testing, according to protocols approved by the Cantonal Ethics Committee of the state of Vaud (CER-VD 479/13, CER-VD 2019-02283, CER-VD 2020-01108). Samples were stored within a dedicated biobank fulfilling quality standards according to the Swiss Biobanking Platform criteria (“Vita label”, certificate CHUV_2004_3).

Patients demographics, comorbidities, symptoms, vital signs and laboratory results performed during routine care were recorded using a standardized electronic case report form in REDCap (Research Electronic Data Capture) or Secutrial. Bedside clinical scores to identify patients at risk of poor outcome were calculated at first assessment in the emergency department: (i) Quick Sequential Organ Failure Assessment (qSOFA): one point each for systolic hypotension (≤100 mmHg), tachypnea (≥22/min) or altered mentation [Glasgow coma score ≤14 (13)], CRB-65: one point each for Confusion (Glasgow coma score ≤14), elevated Respiratory rate (≥30/min), low blood pressure (systolic <90 mmHg or diastolic ≤60 mmHg), age ≥65 years (14). Clinical outcomes were assessed by checking the electronic health record and by calling patients. COVID-19 patients were classified into three groups according to disease severity: (i) outpatients and/or inpatients (e.g. admitted for a motif other than COVID-19) without oxygen requirement (OXY0); (ii) inpatient with oxygen requirement without intubation (OXY1) and (iii) intubation and/or respiratory-related death (TUBE).

### RNA Extraction and Library Preparation

Total RNA from whole blood was isolated using Blood RNA tubes (BD) for blood collection. RNA integrity was assessed with a Bioanalyzer (Agilent Technologies). The TruSeq mRNA stranded kit from Illumina was used for the library preparation with 400 ng of total RNA as input. Library molarity and quality were assessed with the Qubit and Tapestation using a DNA High sensitivity chip (Agilent Technologies). Libraries were pooled at 2 nM and loaded for clustering on several lanes of a Single-read Illumina Flow cell to reach an average of 30 Million of reads per library. Reads of 100 bases were generated using the TruSeq SBS chemistry on an Illumina HiSeq 4000 sequencer.

### Gene Quantification

Transcript abundance quantification was performed with Salmon v1.3.0 (15) in quasi-mapping-based mode using the human reference transcriptome obtained from GENCODE (16) (release 34 corresponding to human genome assembly GRCh38). Default parameters were used plus the –validateMappings –numBootstraps 0 –numGibbsSamples 0 –fldMean 306 –fldSD 30 –seqBias –gcBias parameters. Gene abundances were collected directly with Salmon using –g parameter. Hemoglobin genes ENSG00000206172, ENSG00000188536, ENSG00000244734, ENSG00000229988, ENSG00000223609, ENSG00000213931, ENSG00000213934, ENSG00000196565, ENSG00000206177, ENSG00000086506, ENSG00000130656 and ENSG00000206178 were removed and the remaining genes used to compute the usable library sizes and filter out genes without at least 1 cpm in at least 15 samples (e.g. the size of the smallest group, TUBE early).

### Differential Expression Analyses

Differential gene expression analyses were performed in R [using DESeq2_1.26.0 built-in functions (17)]. Overall similarity between samples was assessed by first applying a variance stabilizing transformation (VST) to the gene-level count matrices using the *vst* function taking into account the experimental design (blind=FALSE), and then performing a principal components analysis (PCA) on the regularized matrix using the *prcomp* function on the 10% most varying genes. To identify differentially expressed genes (DEGs), separate paired analyses between groups were computed using the function *DESeq* with default parameters. Log2 fold changes were moderated with the *lfcShrink* function using the *apeglm* (18) shrinkage estimator and differentially expressed genes were selected based on their multiple testing corrected adjusted P value (<0.01).

### KEGG Pathway Enrichment

DOSE version 3.4.0 (19) and clusterProfiler version 3.6.0 (20) were used to identify pathways with a significant enrichment of DEGs. Analyses were performed separately for each comparison using the *gseKEGG* function with genes ranked by sign (log2FoldChange) * -log10 (pval). Pathways with an adjusted P-value <= 0.05 were considered significantly enriched.

### Gene Set Enrichment Analyses of Functional Groups

Gene set enrichment analyses (GSEA) were performed in R using fgsea version 1.12.0 built in functions (21). Normalized enrichment scores and adjusted P-values were computed separately for each comparison using the *fgsea* function with genes ranked by sign(log2FoldChange) * -log10(pval), the manually curated gene sets reported in **Supplementary Tables 2** **-** **3** and the number of permutations equal to the number of genes in the ranked list (16,670). Gene sets with an adjusted P-value <= 0.05 were considered significantly enriched.

## Results

### Characteristics of Patients Infected With SARS-CoV-2 and Influenza Viruses

Blood gene-expression was measured in 103 patients infected with SARS-CoV-2 (collectively named “COVID-19”) and compared to that of 22 patients infected with Influenza A or B (INFL) as well as 27 non-infected, healthy individuals (HLTY) COVID-19 patients were stratified according to the level of respiratory failure; 23 did not require oxygen support (“OXY0”), 40 received oxygen but no mechanical ventilation (“OXY1”) and 40 required mechanical ventilation (“TUBE”). In the latter group, 15 patients were sampled within the first 7 days in hospital (“TUBE early”) while 25 were sampled later (“TUBE late”).

Patients and controls characteristics are shown in **Supplementary Table 1**. The duration of symptoms before admission was similar in all disease groups. Baseline characteristics and comorbidities were also similar among all COVID-19 patients, except for an over-representation of males among the TUBE late group. As expected, baseline symptoms (fever, cough and dyspnea), signs (temperature, systolic blood pressure and respiratory rate) and severity scores (qSOFA and CURB65) significantly worsened from OXY0 to OXY1 and TUBE patients. INFL patients were significantly older than COVID-19 patients (mean age 73 versus mean ages ranging from 54 in OXY0 to 64 in TUBE early), but had a similar pattern of comorbidities, except for hypertension, dyslipidemia and gastrointestinal diseases, which were significantly less frequent in INFL than OXY1. Their qSOFA score was comparable to that of OXY0 and OXY1 while the CURB65 score and hospitalization rate was comparable to those of TUBE patients. INFL were significantly more likely to have fever, cough, fatigue, leukocytosis and thrombocytopenia at baseline than OXY0. In contrast, they were less likely to have hypotension, dyspnea, tachypnea, leukocytosis and radiological infiltrates at baseline; and death at 30 days than OXY1 and TUBE early.

### Overall Transcriptomic Profile of Patients Infected With SARS-CoV-2 and Influenza Viruses

Total RNA libraries produced on average 34.1 million reads (standard deviation, SD=4.7), among which an average of 30.6 million (SD=4.6) were mapped against the 228,048 transcripts of Gencode v34 and aggregated at the gene level using Salmon v1.3.0. After removal of hemoglobin genes, all samples had produced at least 10 millions of usable reads, except samples from subjects TUBE early.01 and TUBE late.05, which had 9.9 and 9.8 million reads, respectively (**Supplementary Figure 1**). The mean number of usable reads for HLTY, patients infected with SARS-CoV-2 (OXY0, OXY1, TUBE-early and TUBE-late) and INFL was 17.3, 24.0, 24.4, 21.6, 16.4 and 22.1 millions, respectively (**Supplementary Figure 1**). Sex was confirmed by counting the number of reads mapping on the male and female specific genes RPS4Y1 (ENSG00000129824.16) and XIST (ENSG00000229807.12, **Supplementary Figure 1**). The 16,670 genes with at least 1 cpm in at least 15 samples (e.g. the size of the smallest group) were kept for further analysis.

**Figure 1:**
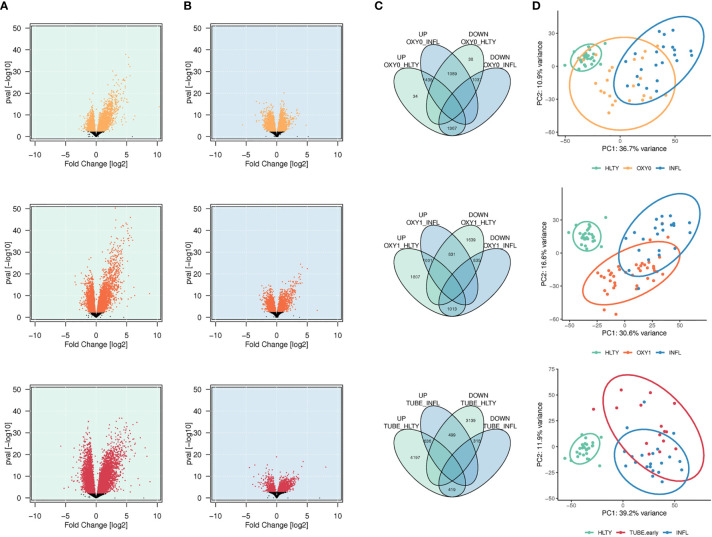
Differential genes expression analysis. **(A, B)** Volcano plots showing the distribution of log2 fold changes and P-values of differentially expressed genes (DEGs) among SARS-Cov-2 infected patients represented in yellow (no oxygen support, OXY0), orange (oxygen support, OXY1) and red (intubated with sampling within 7 days in hospital, TUBE EARLY), respectively, using non-infected controls (HEALTHY, green shadow, **A**) and Influenza-infected patients (INFLUENZA, blue shadow, **B**), as a reference group. Genes with a -log10 (P-value) not significant once adjusted for multiple testing (adjusted P > 0.01) are represented in black **(C)**. Venn diagram showing the number of up or down regulated DEGs in each comparison, and number of common DEGs between comparisons. **(D)** Principal component analysis of gene expression in Influenza, severe and non-severe SARS-CoV-2 infected patients. The figure presents the two major principal components using the 10% of most significant DEGs. Ellipses were drawn using the stat-ellipse function of the R ggplot2 package, using default parameters.

A total of 2,839, 3,821, 5,252 genes had higher expression (adjusted P<= 0.01), and a total of 2,164, 3,305 and 3,956 genes had lower expression, among OXY0, OXY1 and TUBE early, respectively, compared to HLTY (**Figure 1A**). A total of 2,527, 1,832, and 1,135 genes had higher expression, and a total of 2,404, 1,848 and 737 genes had lower expression, among OXY0, OXY1 and TUBE, respectively, compared to INFL (**Figure 1B**). The number of upregulated genes compared to both reference groups (HLTY and INFL) are 1,438, 1,001 and 636 in OXY0, OXY1 and TUBE early, respectively (**Figure 1C** **),** whereas the number of down-regulated genes compared to both reference groups are 1,037, 835 and 318 (**Figure 1C**). The two first principal components of the 10% most varying gene expression across all samples explained 47.6%, 46.1% and 51.1% of variance, respectively, when comparing OXY0, OXY1 and TUBE early groups with HTLY and INFL (**Figure 1D**).

An unsupervised analysis was performed by using gene pathways sets from the Kyoto Encyclopedia of Genes and Genomes (22, 23) (KEGG, **Supplementary Figure 2**). Compared to HTLY, TUBE early and INFL had over-expression of genes involved in “human diseases”, including mainly infections (e.g. Influenza A [hsa05164], Epstein-Barr virus infection [hsa05169] or Legionellosis [hsa05134]), but also cancer (e.g. Transcriptional misregulation in cancer [hsa05202]) and inflammatory diseases (e.g. Systemic lupus erythematosus [hsa05322]). However, gene expression was not uniform across both viral infections, with significant under-expression in COVID-19 compared to INFL patients. Core-enriched genes from infectious diseases genes sets largely overlapped with those from immune pathways, encompassing both innate (e.g. Toll-like receptor [hsa04620], TLRs, NOD-like receptor signaling [hsa04621], NLRs, RIG-I like receptor signaling [hsa04622]) and adaptive immunity (e.g. Natural Killer cell mediated cytotoxicity [hsa04650], T [hsa04660] and B [hsa04662] cells signaling), which were also under-expressed among COVID-19 compared to INFL. Under-expression tended to decrease with increased COVID-19 severity. Conversely, a group of pathways covering “cellular processes” (e.g. cell cycle [hsa04110]) and “metabolism” (e.g. oxidative phosphorylation [hsa00190]), sharing a number of core-enriched genes, were up-regulated in COVID-19 compared to INFL, with a gradient from less to more severe COVID-19.

### Immunological Transcriptomic Features of Patients Infected With SARS-CoV-2 and Influenza Viruses

**Figure 2:**
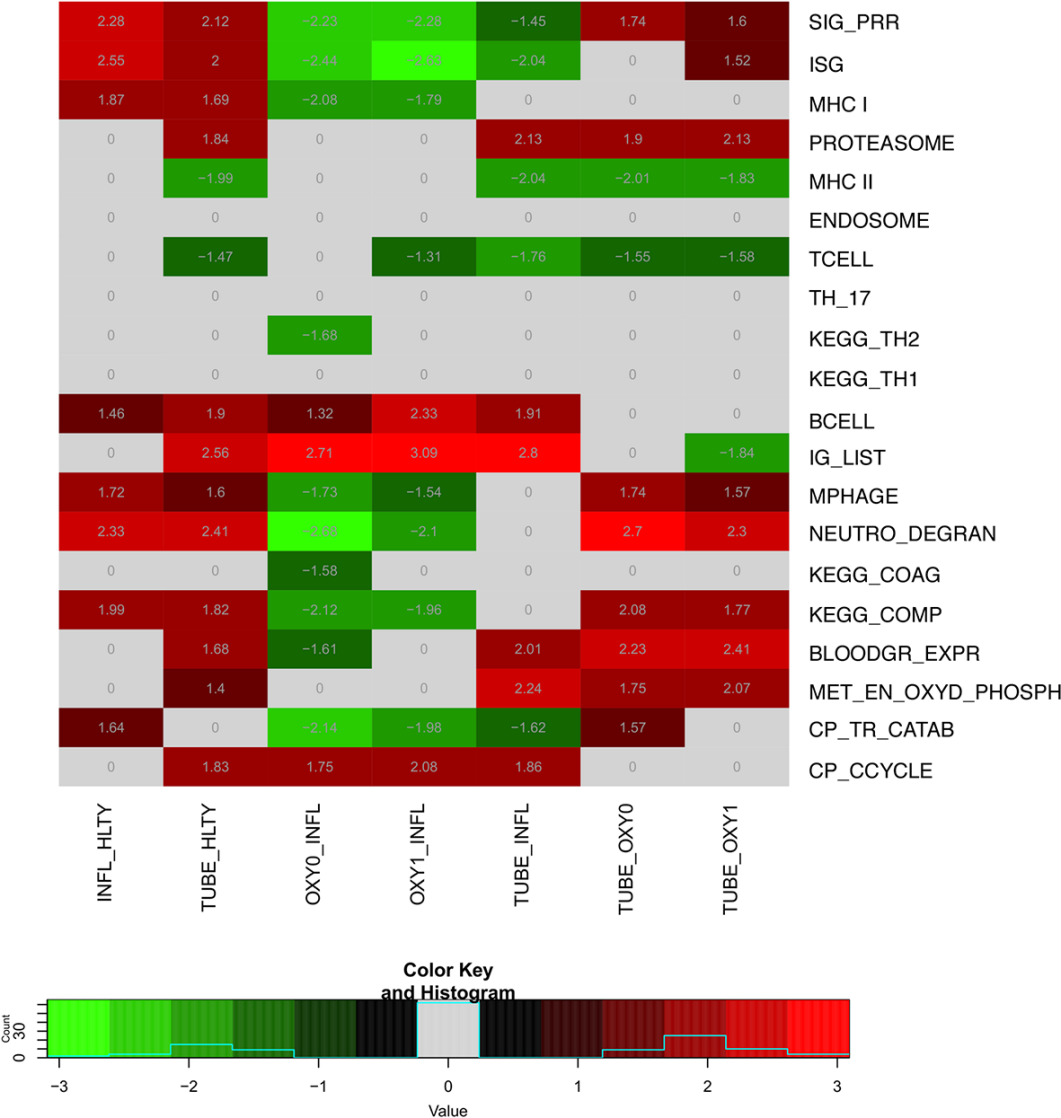
Heatmap of differentially expressed genes (DEGs) sets for indicated pairwise comparisons. Groups used for comparisons (columns) include SARS-Cov-2 infected patients with no oxygen support [OXY0], oxygen support [OXY1], intubation with sampling within 7 days (TUBE early) or >7 days (TUBE late) after hospital admission, Influenza virus (INFL) infected patients and non-infected controls (HLTY). Significantly (adj. P-Value <=0.05) enriched genes sets (rows) are selected from different GO, REACTOME and/or KEGG pathways (see **Supplementary Table 2**), and their normalized enrichment score (NES) is indicated in each cell and used for coloring.

To translate the impact of differentially expressed genes (DEGs) in terms of biological responses, we systematically analyzed sets of genes matching specific immune processes (**Figure 2**). Representative sets available from KEGG (22), Gene Ontology [GO (24, 25)], Reactome (26) or Human Genome Organization (HUGO) Gene Nomenclature Committee [HGNC (27)] were selected (**Supplementary Table 2**) and shown by heatmaps and/or volcano plots, with specific genes data detailed on box-plots. For the sake of simplicity, expression patterns across the different groups were regarded as “ascending” or “descending”, when expression levels progressively increased, or decreased, respectively, from HLTY to OXY0, OXY1, TUBE early, and IFNL (**Supplementary Figure 3** first and second rows). Patterns were qualified to have a “hill” or “valley” shape, when highest and lowest expression levels were seen in COVID-19 (OXY1 or TUBE early), and the lowest and highest in the other (HLTY and INFL), respectively (**Supplementary Figure 3** third and fourth rows). Because the TUBE late group often represented a convalescent status, their gene expression levels were ignored in this pattern description.

**Figure 3:**
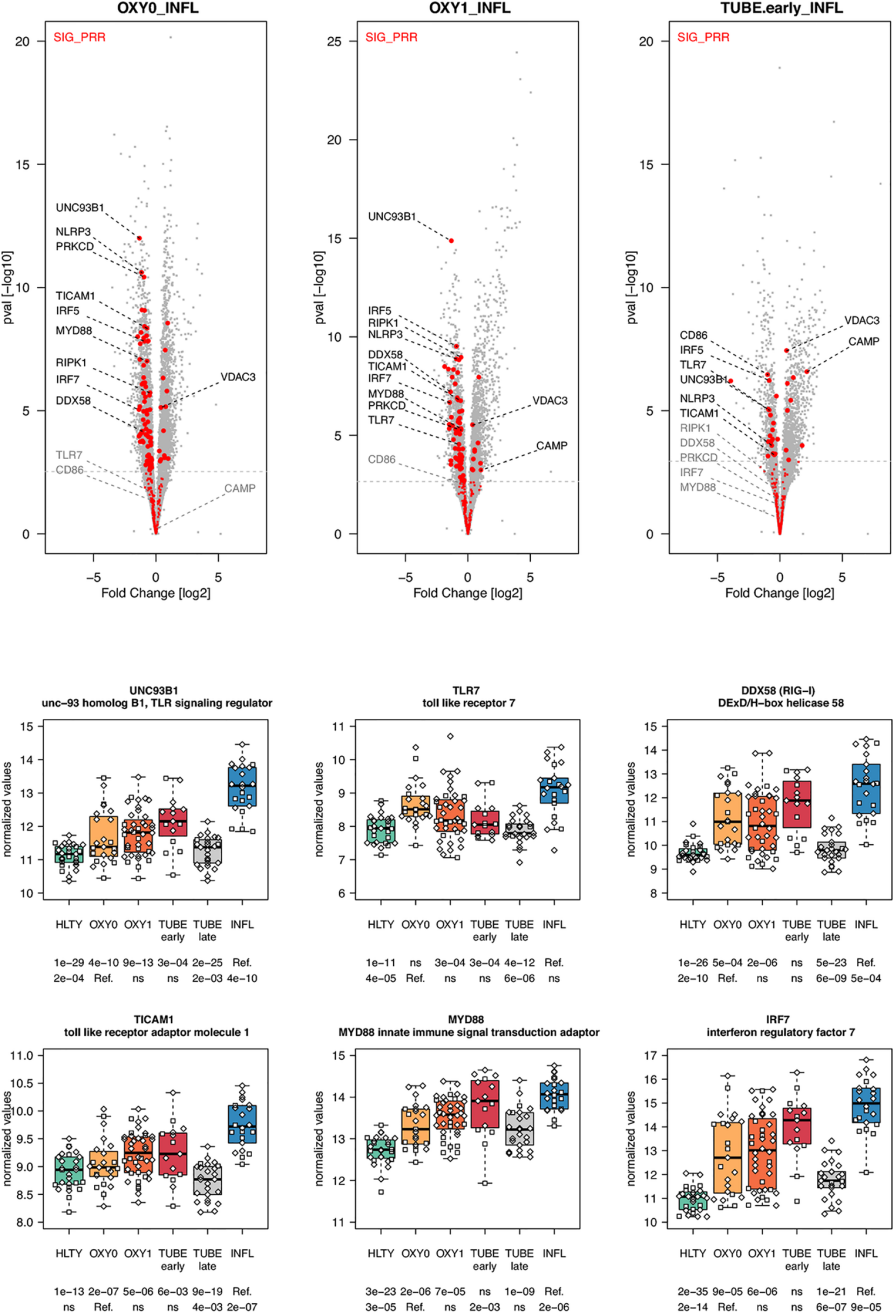
Differentially expressed genes (DEGs) involved in innate immune detection of viruses. Upper panel: for each comparison indicated in the volcano plot header, the distribution of log2 fold changes and P-values of all detected genes is shown as gray dots. Red dots indicate genes included in the SIG_PRR gene set (innate immune detection of viruses and downstream signal transduction). Genes’ sets are selected from different GO, REACTOME and/or KEGG pathways (see **Supplementary Table 2**). The dotted horizontal gray line indicates the limit under which –log10(P-value) becomes non significant once adjusted for multiple testing (adjusted P > 0.01). Lower panel shows detailed expression levels for selected genes. Groups used for comparisons include SARS-Cov-2 infected patients (no oxygen support [OXY0], oxygen support [OXY1], intubation with sampling within 7 days [TUBE early] or >7 days [TUBE late] after hospital admission), Influenza virus [INFL] infected patients and non-infected controls [HLTY]. Adjusted P-values of pairwise comparisons between a reference group (Ref.) and another group are indicated below the x axis. Ref. is taken as [INFL] and [OXY0] on the first and second line, respectively.

**Figure 4:**
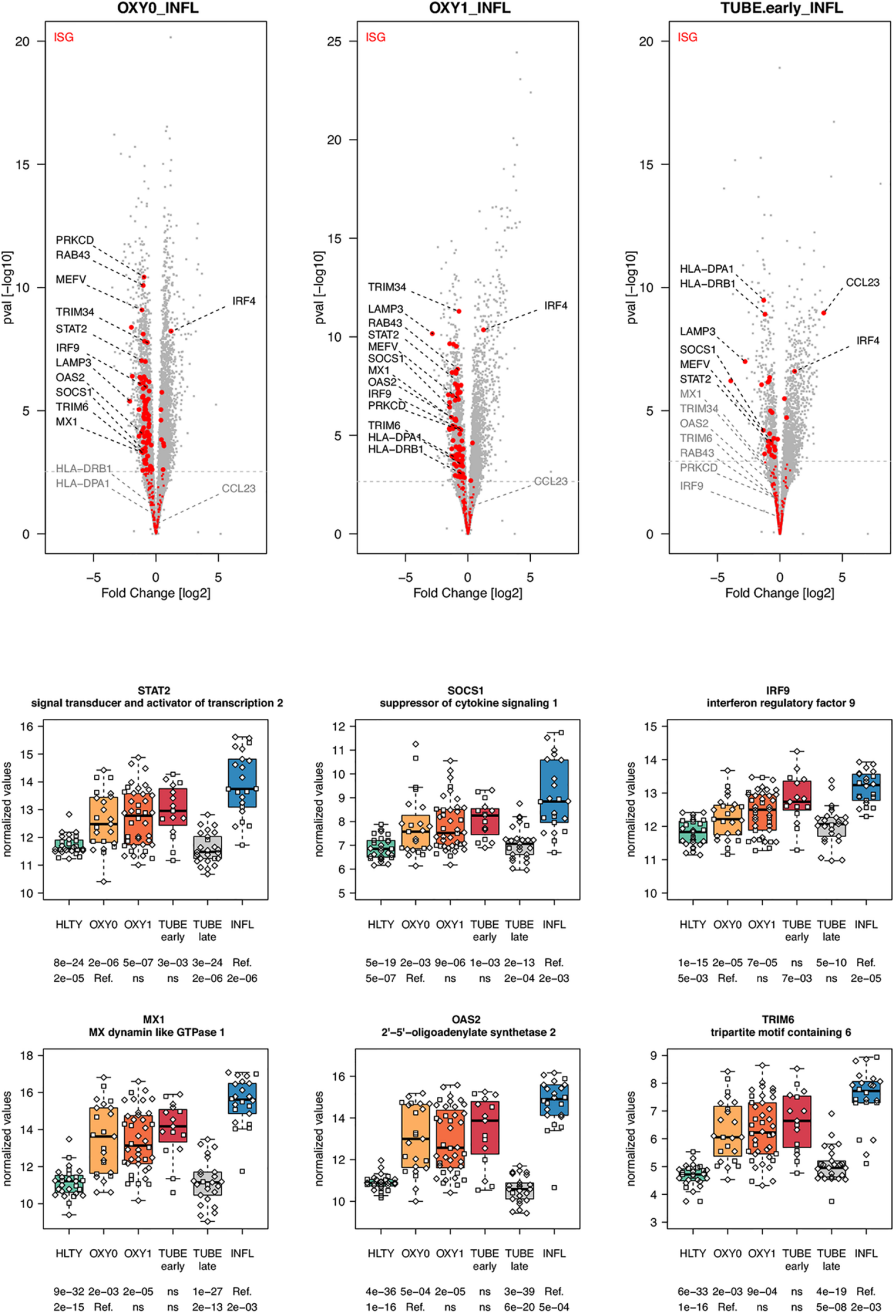
Differentially expressed interferon-stimulated genes. Upper panel: for each comparison indicated in the volcano plot header, the distribution of log2 fold changes and P-values of all detected genes is shown as gray dots. Red dots indicate genes included in the ISGs (interferon-stimulated genes) gene set. Genes’ sets are selected from different GO, REACTOME and/or KEGG pathways (see **Supplementary Table 2**). The dotted horizontal gray line indicates the limit under which –log10(P-value) becomes non significant once adjusted for multiple testing (adjusted P > 0.01). Lower panel shows detailed expression levels of selected genes involved in IFN signal transduction and IFN stimulated genes in the different groups of patients. Groups used for comparisons include SARS-Cov-2 infected patients (no oxygen support [OXY0], oxygen support [OXY1], intubation with sampling within 7 days [TUBE early] or >7 days [TUBE late] after hospital admission), Influenza virus [INFL] infected patients and non-infected controls [HLTY]. Adjusted P-values of pairwise comparisons between a reference group (Ref.) and another group are indicated below the x axis. Ref. is taken as [INFL] and [OXY0] on the first and second line, respectively.

When considering innate immune pathways, we first analyzed sets of genes encoding PRR and their signaling effectors, ISGs as well as cytokines/chemokines. Most genes involved in antiviral signaling were over-expressed both in TUBE early and INFL patients compared to HLTY (**Supplementary Figure 4**; pathways involved in the detection of Influenza A and other RNA viruses), but gene expression was significantly lower among COVID-19 than among INFL **(** **Figure 3** **)**. Most striking was the under-expression of genes involved in viral recognition (e.g. *TLR7, UNC93B, RIG-I/DDX58*, **Figure 3**), as well as genes encoding downstream signaling molecules and transcription factors (e.g. *TRIF/TICAM1, MYD88, IRF7*, **Figure 3**), with the notable exception of *IRF4*, in COVID-19 compared to INFL (**Figure 4**). Moreover, compared to INFL there was also a significant under-expression of genes involved in interferons signaling and ISGs (e.g. *STAT2, IRF9, SOCS1, MX1, OAS2, TRIM6*, **Figure 4**). Most of these genes had expression levels increasing from less to more severe COVID-19 (“ascending” pattern). Gene expression levels for cytokines and chemokines could not always be assessed (**Supplementary Figure 5**); when measurable, their expression levels and that of their receptors tended to be lower among COVID-19 compared to INFL, although the pattern was not uniform and depended on structural groups. In particular, genes coding for cytokines from the IL-4-like and IL-1-like subfamilies had lower expression levels among OXY0 and/or OXY1, whereas those belonging to the TGF-B family tended to have higher expression levels among OXY0, compared to INFL.

**Figure 5:**
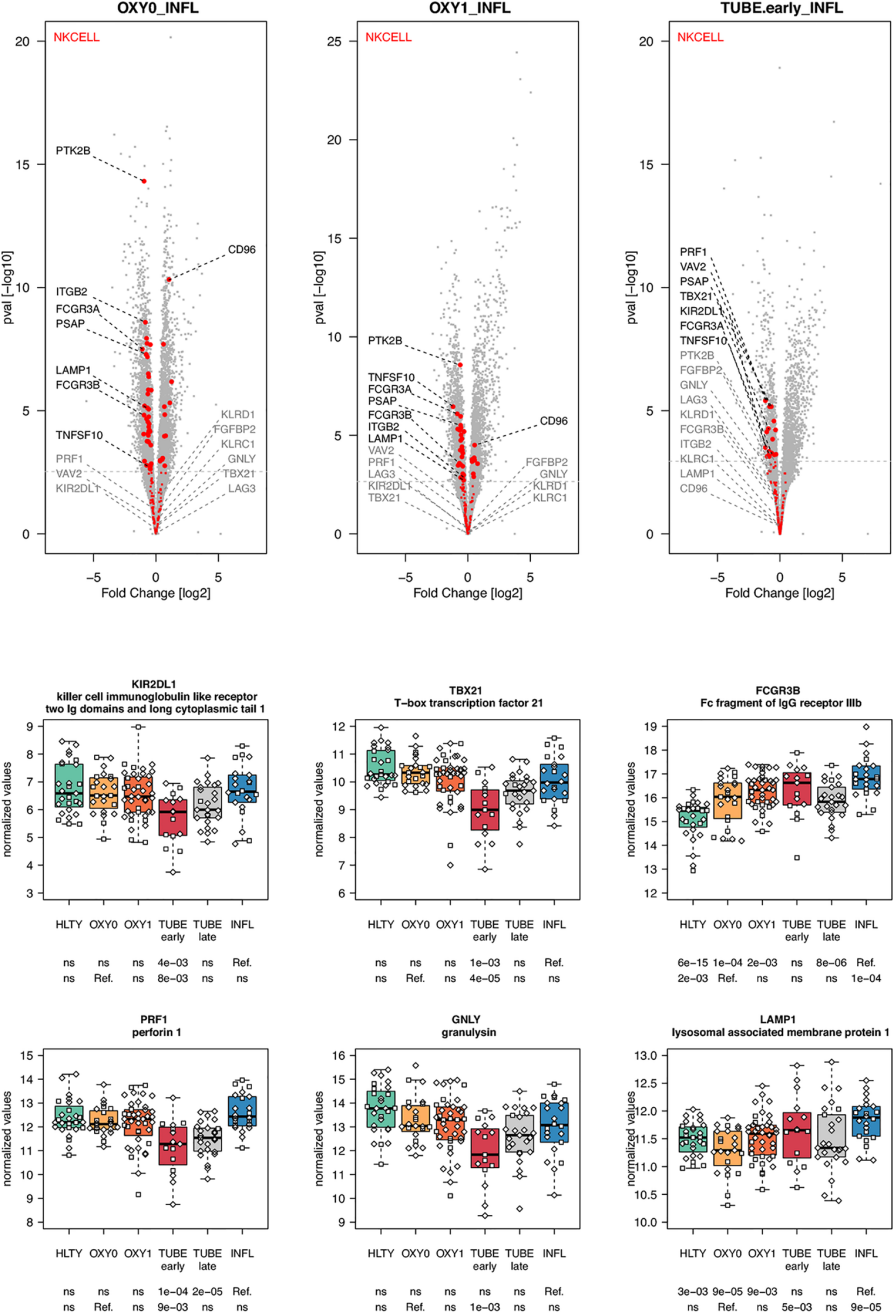
Differentially expressed genes (DEGs) involved in NK cells function and regulation. Upper panel: for each comparison indicated in the volcano plot header, the distribution of log2 fold changes and P-values of all detected genes is shown as gray dots. Red dots indicate genes involved in NK cells function and regulation. Genes’ sets are selected from different GO, REACTOME and/or KEGG pathways (see **Supplementary Table 2**). The dotted horizontal gray line indicates the limit under which –log10(P-value) becomes non significant once adjusted for multiple testing (adjusted P > 0.01). Lower panel shows detailed expression levels of selected genes involved in NK cells function and regulation in the different groups of patients. Groups used for comparisons include SARS-Cov-2 infected patients (no oxygen support [OXY0], oxygen support [OXY1], intubation with sampling within 7 days [TUBE early] or >7 days [TUBE late] after hospital admission), Influenza virus [INFL] infected patients and non-infected controls [HLTY]. Adjusted P-values of pairwise comparisons between a reference group (Ref.) and another group are indicated below the x axis. Ref. is taken as [INFL] and [OXY0] on the first and second line, respectively.

**Figure 6:**
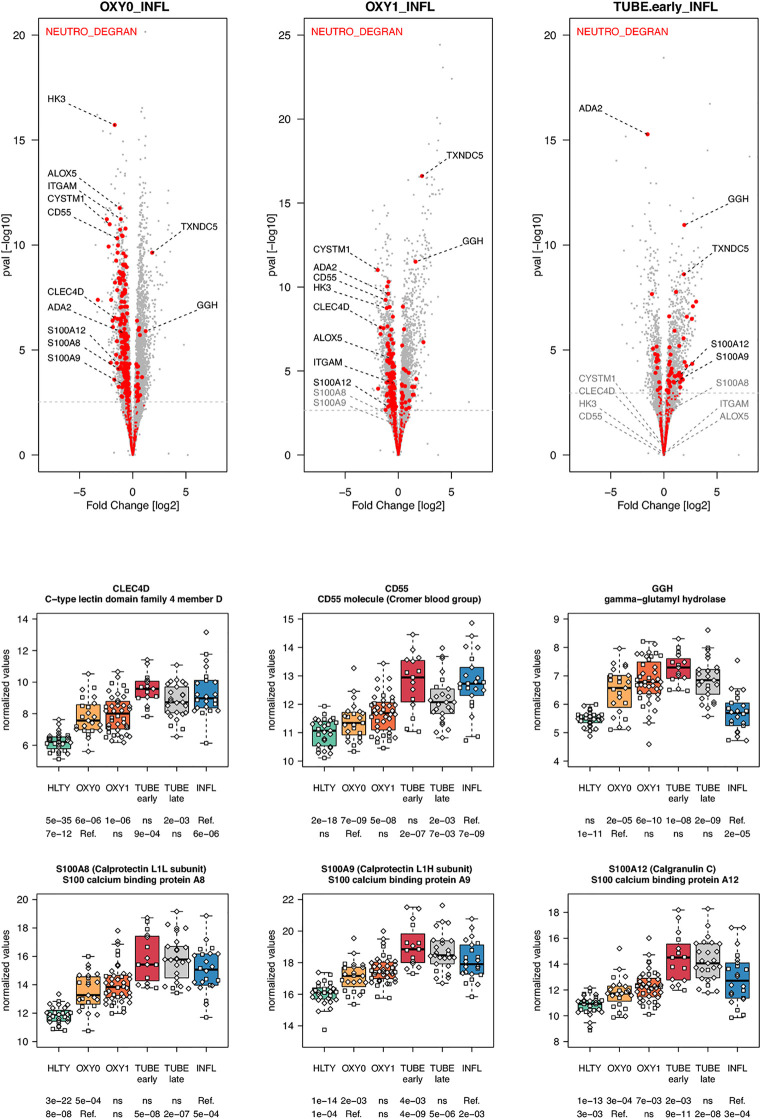
Differentially expressed genes (DEGs) involved in macrophages and neutrophils functions. Upper panel: for each comparison indicated in the volcano plot header, the distribution of log2 fold changes and P-values of all detected genes is shown as gray dots. Red dots indicate genes involved in neutrophils degranulation. Genes’ sets are selected from different GO, REACTOME and/or KEGG pathways (see **Supplementary Table 2**). The dotted horizontal gray line indicates the limit under which –log10(P-value) becomes non significant once adjusted for multiple testing (adjusted P > 0.01). Lower panel shows detailed expression levels of selected genes involved in macrophages function and neutrophils degranulation in the different groups of patients. Groups used for comparisons include SARS-Cov-2 infected patients (no oxygen support [OXY0], oxygen support [OXY1], intubation with sampling within 7 days [TUBE early] or >7 days [TUBE late] after hospital admission), Influenza virus [INFL] infected patients and non-infected controls [HLTY]. Adjusted P-values of pairwise comparisons between a reference group (Ref.) and another group are indicated below the x axis. Ref. is taken as [INFL] and [OXY0] on the first and second line, respectively.

When considering innate immune cells, we first observed that genes involved in NK cell functions had an overall lower expression levels in TUBE early compared to HLTY (**Supplementary Figure 6** right panel) and in each COVID-19 group compared to INFL (**Figure 5** and **Supplementary Figure 6** left panel). Some genes important for NK cell maturation were significantly under-expressed, with a “valley” pattern (e.g. Killer-cell inhibitory-receptors such as *KIR2DL1* or *KIR3DL2*, as well as *TBX21*) or an “ascending” pattern (*FCGR3B*, **Figure 5**). Genes important for NK cell cytotoxicity also harbored a “valley” (perforin/*PRF1*, granulysin/*GNLY*, *KSP37/FGFBP2*) or “ascending” pattern (*CD107a/LAMP1*). In contrast, *NKG2A/KLRC1* and *LAG3* were not overexpressed and *CD94/KLRD1* was even under-expressed among TUBE early compared to both HTLY (**Supplementary Figure 6** left panel) and INFL (**Figure 5**). Genes involved in macrophage and neutrophils were over-expressed in patients infected with viruses (COVID-19 and INFL) compared to HLTY (“ascending” pattern, e.g. *CLEC4D* and *CD55*, **Figure 6**). Genes involved in neutrophil degranulation (e.g. GGH) and/or other neutrophil functions (*S100A8/9* and *S100A12*) had a peak of expression in the TUBE early group (“hill” pattern, **Figure 6**).

**Figure 7:**
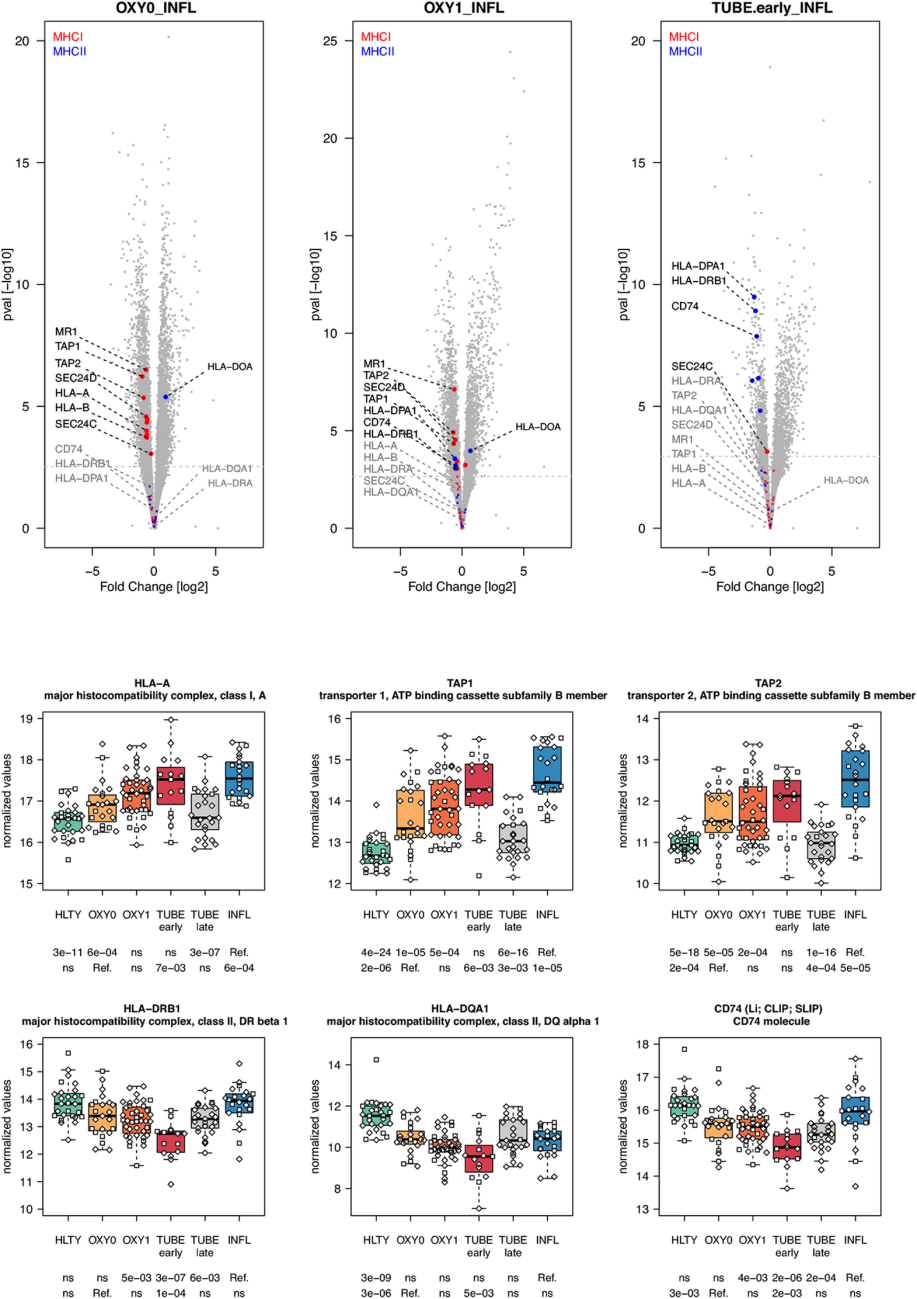
Differentially expressed genes (DEGs) involved in antigen presentation. Upper panel: for each comparison indicated in the volcano plot header, the distribution of log2 fold changes and P-values of all detected genes is shown as gray dots. Red and blue dots indicate genes involved in antigen presentation through the MHC I and MHCII respectively. Genes’ sets are selected from different GO, REACTOME and/or KEGG pathways (see **Supplementary Table 2**). The dotted horizontal gray line indicates the limit under which –log10(P-value) becomes non significant once adjusted for multiple testing (adjusted P > 0.01). Lower panel shows detailed expression levels of selected genes involved in antigen presentation through the MHCI and MHC II in the different groups of patients. Groups used for comparisons include SARS-Cov-2 infected patients (no oxygen support [OXY0], oxygen support [OXY1], intubation with sampling within 7 days [TUBE early] or >7 days [TUBE late] after hospital admission), Influenza virus [INFL] infected patients and non-infected controls [HLTY]. Adjusted P-values of pairwise comparisons between a reference group (Ref.) and another group are indicated below the x axis. Ref. is taken as [INFL] and [OXY0] on the first and second line, respectively.

Adaptive immune pathways were notable for genes involved in antigen presentation through MHCI with proteasome, MHCII with endosome, T helper differentiation, proliferation/maturation/function of T and B cells. Genes involved in antigen presentation through the MHC I had higher expression levels among virus-infected patients (TUBE early and INFL) compared to HLTY, with an “ascending” pattern (e.g. *HLA-A, HLA-B, TAP-1, TAP-2*, **Figure 7** and **Supplementary Figure 7**). In contrast, genes involved in antigen presentation through MHC II presented a “valley” pattern, with under-expression among TUBE early compared to HLTY and COVID-19 compared to INFL, which strikingly predominated in TUBE early (e.g. *HLA-DRA, HLA-DRB1, HLA-DQA1, CD74/Ii/CLIP/SLIP*, **Figure 7**).

**Figure 8:**
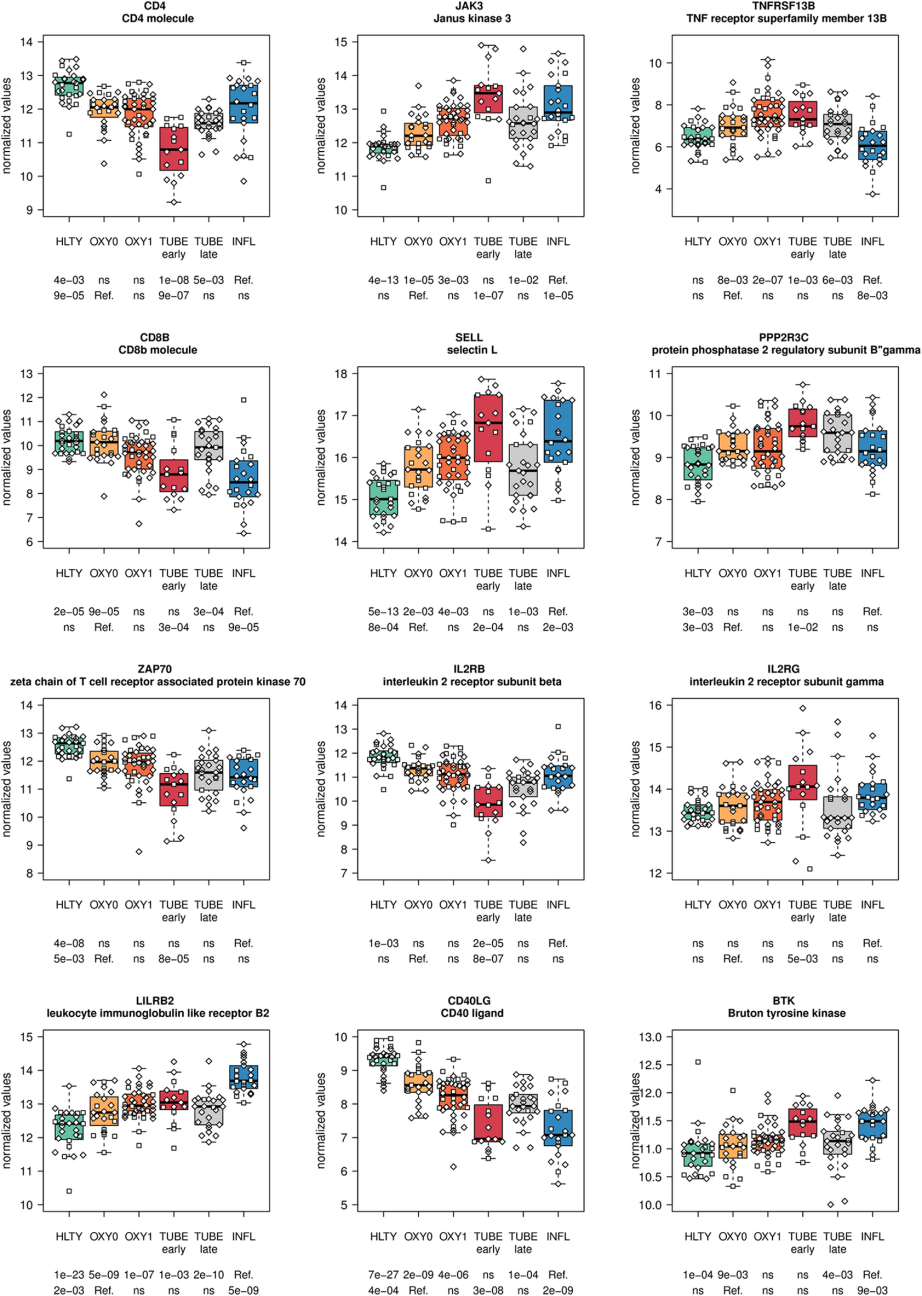
Differentially expressed genes (DEGs) involved in T cells and B cells functions. Expression levels of selected genes involved in T and B cells function and regulation. Groups used for comparisons include SARS-Cov-2 infected patients (no oxygen support [OXY0], oxygen support [OXY1], intubation with sampling within 7 days [TUBE early] or >7 days [TUBE late] after hospital admission), Influenza virus [INFL] infected patients and non-infected controls [HLTY]. Adjusted P-values of pairwise comparisons between a reference group (Ref.) and another group are indicated below the x axis. Ref. is taken as [INFL] and [OXY0] on the first and second line, respectively.

Genes involved in T cell functions had contrasting expression levels (**Supplementary Figures 8** **,** **9** **)**. A majority of them had a “valley” (e.g. *CD4, ZAP70*, **Figure 8**; *ADA, GATA3*, **Supplementary Figure 8**) or “descending” pattern, with expression levels decreasing from HLTY to COVID-19 and INFL (e.g. *CD8*, **Figure 8**; *IL7R/IL7Rα, CD3D, IL2RA* **Supplementary Figure 8**). However, some genes still presented an “ascending” pattern, such as the regulatory gene *LILRB2* (**Figure 8**) or a “hill” pattern (e.g. *JAK3, IL2RG/γC* **Figure 8**). When focusing on T cell subclasses, some markers of memory were over-expressed (e.g. *SELL/CD62L* **Figure 8**, *CD103/ITGAE* **Supplementary Figure 8**), while others were under-expressed or unchanged (e.g. *CCR7, CD27, CD28* **Supplementary Figure 8**) in COVID-19 compared to HLTY and INFL, without matching a specific type of memory T cell. Similarly, some markers of T cell activation were over-expressed (e.g. *CD38, KI-67/MKI67* **Supplementary Figure 8**) while other were under-expressed (e.g. *IL2RB/CD122*, **Figure 8**, *CD69*, **Supplementary Figure 8**) among COVID-19 compared to INFL or HTLY. There were no distinct expression patterns in genes involved in T helper differentiation, except for a decreased expression in TH2-related genes among OXY0 compared to INFL (**Supplementary Figures 10** **-** **11**). Finally, exhaustion genes such as *PDCD1*/*PD-1* or regulatory genes such as *FOXP3* were under-expressed among COVID-19 compared to INFL or HTLY, mainly among TUBE early patients (**Supplementary Figure 8** **)**.

**Figure 9:**
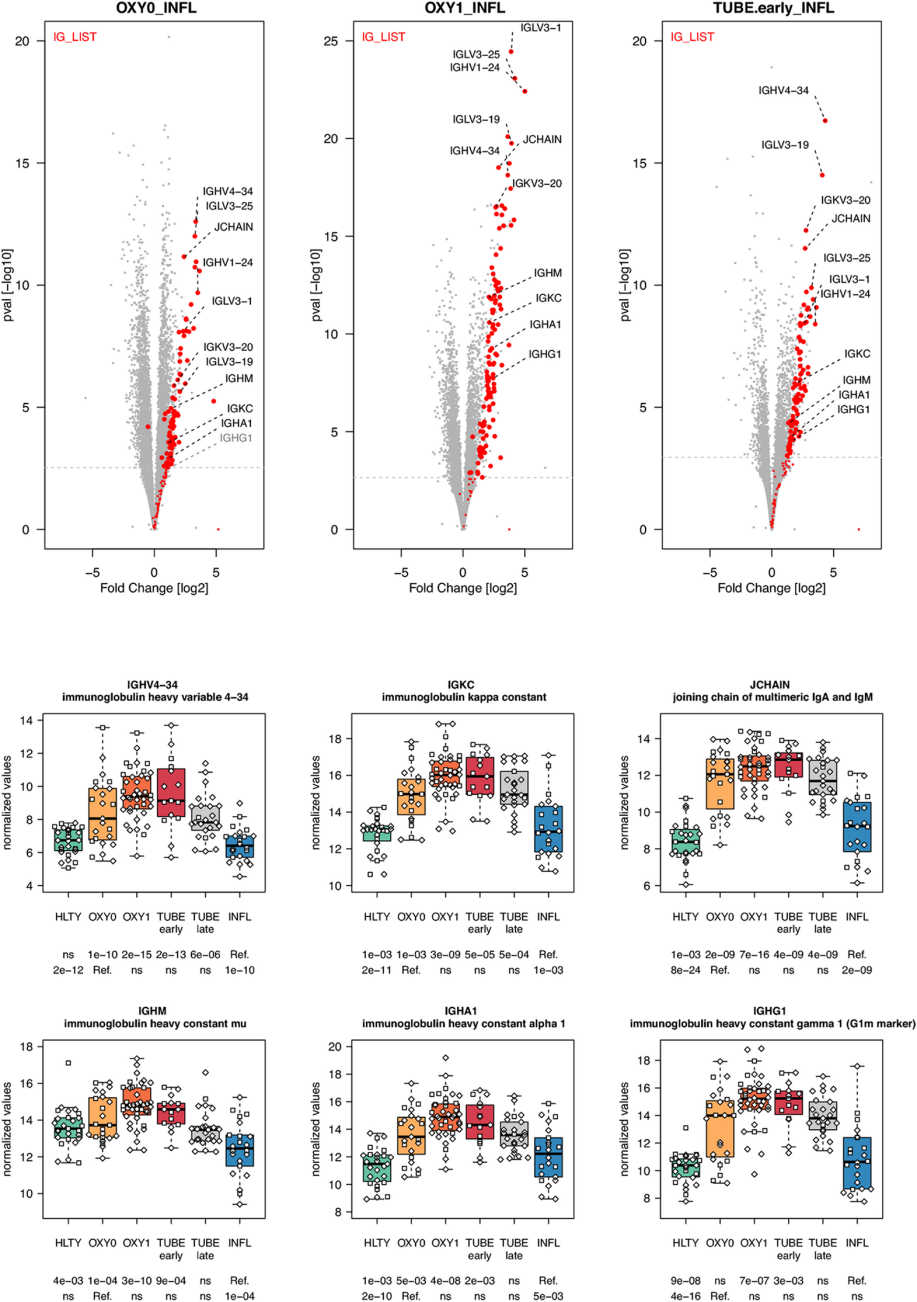
Differentially expressed immunoglobulin encoding genes. Upper panel: for each comparison indicated in the volcano plot header, the distribution of log2 fold changes and P-values of all detected genes is shown as gray dots. Red dots indicate immunoglobulin encoding genes. Genes’ sets are selected from different GO, REACTOME and/or KEGG pathways (see **Supplementary Table 2**). The dotted horizontal gray line indicates the limit under which –log10(P-value) becomes non significant once adjusted for multiple testing (adjusted P > 0.01). Lower panel shows detailed expression levels of selected immunoglobulin encoding genes in the different groups of patients. Groups used for comparisons include SARS-Cov-2 infected patients (no oxygen support [OXY0], oxygen support [OXY1], intubation with sampling within 7 days [TUBE early] or >7 days [TUBE late] after hospital admission), Influenza virus [INFL] infected patients and non-infected controls [HLTY]. Adjusted P-values of pairwise comparisons between a reference group (Ref.) and another group are indicated below the x axis. Ref. is taken as [INFL] and [OXY0] on the first and second line, respectively.

In general, genes involved in B cell functions had higher expression levels among TUBE early than HLTY (**Supplementary Figure 12** lower panel). However, the patterns were inconsistent; some genes showed a “hill” pattern (*ILR2G, TNFRSF13B/TACI, PPP2R3C*, **Figure 8**), while other showed a “descending” pattern (*CD40LG*, **Figure 8**, *CD19, UNG*, **Supplementary Figure 13**), as opposed to the “ascending” pattern observed for the Bruton’s tyrosine kinase (*BTK*, **Figure 8**). Immunoglobulin-encoding genes were markedly over-expressed in COVID-19 compared to HLTY and INFL, and peaked in OXY1/TUBE EARLY (“hill” pattern, e.g. *IGHM, IGHA1, IGHG1, JCHAIN*, **Figure 9**).

**Figure 10:**
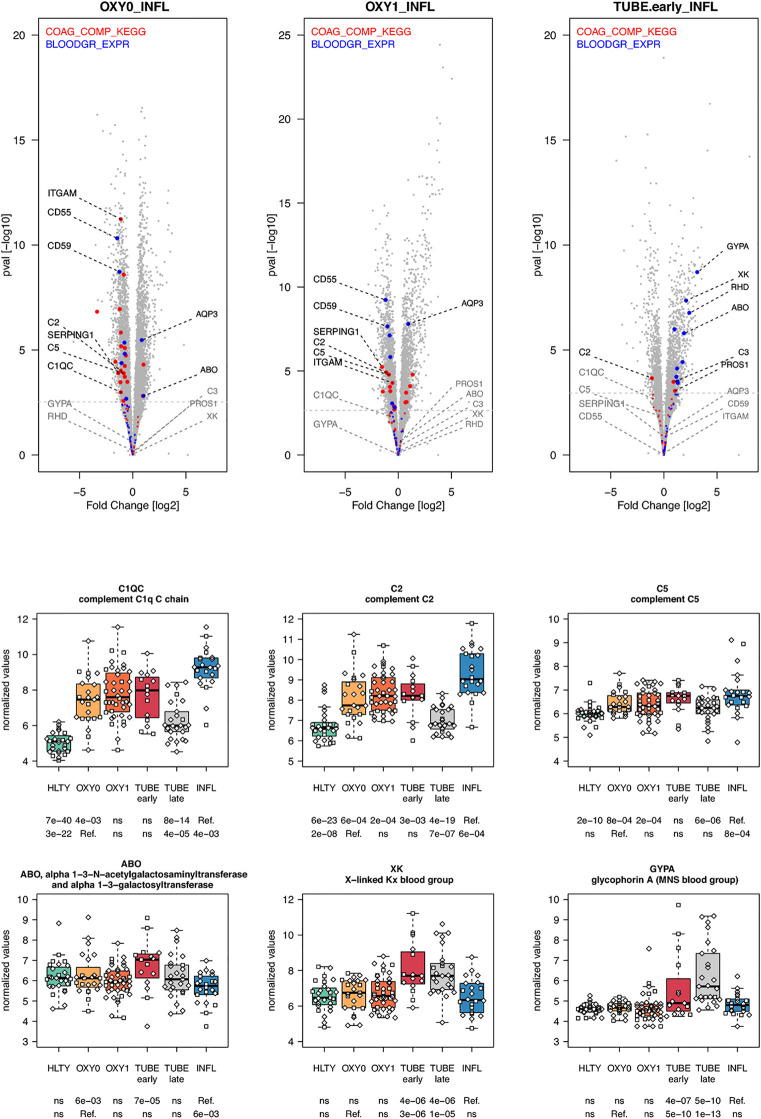
Differentially expressed genes (DEGs) involved in complement system and blood group. Upper panel: for each comparison indicated in the volcano plot header, the distribution of log2 fold changes and P-values of all detected genes is shown as gray dots. Red and blue dots indicate genes involved in complement system and blood group respectively. Genes’ sets are selected from different GO, REACTOME and/or KEGG pathways (see **Supplementary Table 2**). The dotted horizontal gray line indicates the limit under which –log10(P-value) becomes non significant once adjusted for multiple testing (adjusted P > 0.01). Lower panel shows detailed expression levels of selected genes involved in complement system and blood groups in the different groups of patients. Groups used for comparisons include SARS-Cov-2 infected patients (no oxygen support [OXY0], oxygen support [OXY1], intubation with sampling within 7 days [TUBE early] or >7 days [TUBE late] after hospital admission), Influenza virus [INFL] infected patients and non-infected controls [HLTY]. Adjusted P-values of pairwise comparisons between a reference group (Ref.) and another group are indicated below the x axis. Ref. is taken as [INFL] and [OXY0] on the first and second line, respectively.

Most genes involved in the complement cascade were over-expressed in TUBE early and INFL compared to HTLY (**Supplementary Figure 14** right panel) but their expression was usually lower among COVID-19 compared to INFL (“ascending” pattern, e.g. *C1QC, C2, C5*), with the exception of *C3*, that, was over-expressed in TUBE early compared to INFL patients (**Figure 10** and **Supplementary Figure 14** left panel). Genes encoding blood groups, some of which are part of the complement cascade (e.g. *C4A, C4B*) or are involved in complement activation (e.g. *CR1, CD55, ABO*) or regulation (e.g.*CD59, GYPA*, *GYPB*) were overexpressed solely among TUBE patients (e.g. *ABO, GYPA, XK*, **Figure 10** and **Supplementary Figure 15**). Overall, there was no differential expression for genes involved in the coagulation cascade, except for a decreased expression among OXY0 compared to INFL.

**Figure 11:**
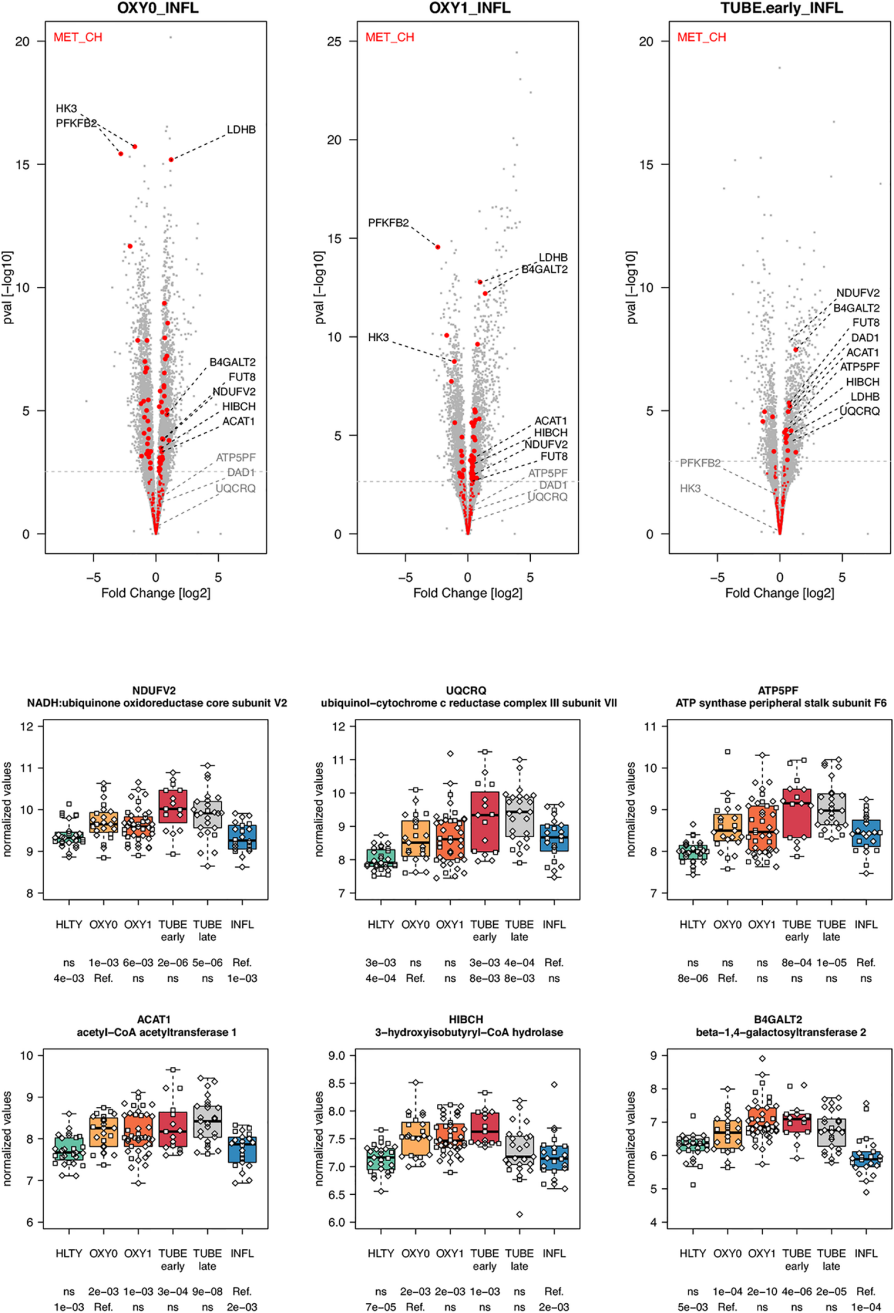
Differentially expressed genes (DEGs) involved in cell metabolism. Upper panel: for each comparison indicated in the volcano plot header, the distribution of log2 fold changes and P-values of all detected genes is shown as gray dots. Red dots indicate genes involved in cell metabolism. Genes’ sets are selected from different GO, REACTOME and/or KEGG pathways (see **Supplementary Table 2**). The dotted horizontal gray line indicates the limit under which –log10(P-value) becomes non significant once adjusted for multiple testing (adjusted P > 0.01). Lower panel shows detailed expression levels of selected genes involved in cell metabolism in the different groups of patients. Groups used for comparisons include SARS-Cov-2 infected patients (no oxygen support [OXY0], oxygen support [OXY1], intubation with sampling within 7 days [TUBE early] or >7 days [TUBE late] after hospital admission), Influenza virus [INFL] infected patients and non-infected controls [HLTY]. Adjusted P-values of pairwise comparisons between a reference group (Ref.) and another group are indicated below the x axis. Ref. is taken as [INFL] and [OXY0] on the first and second line, respectively.

**Figure 12:**
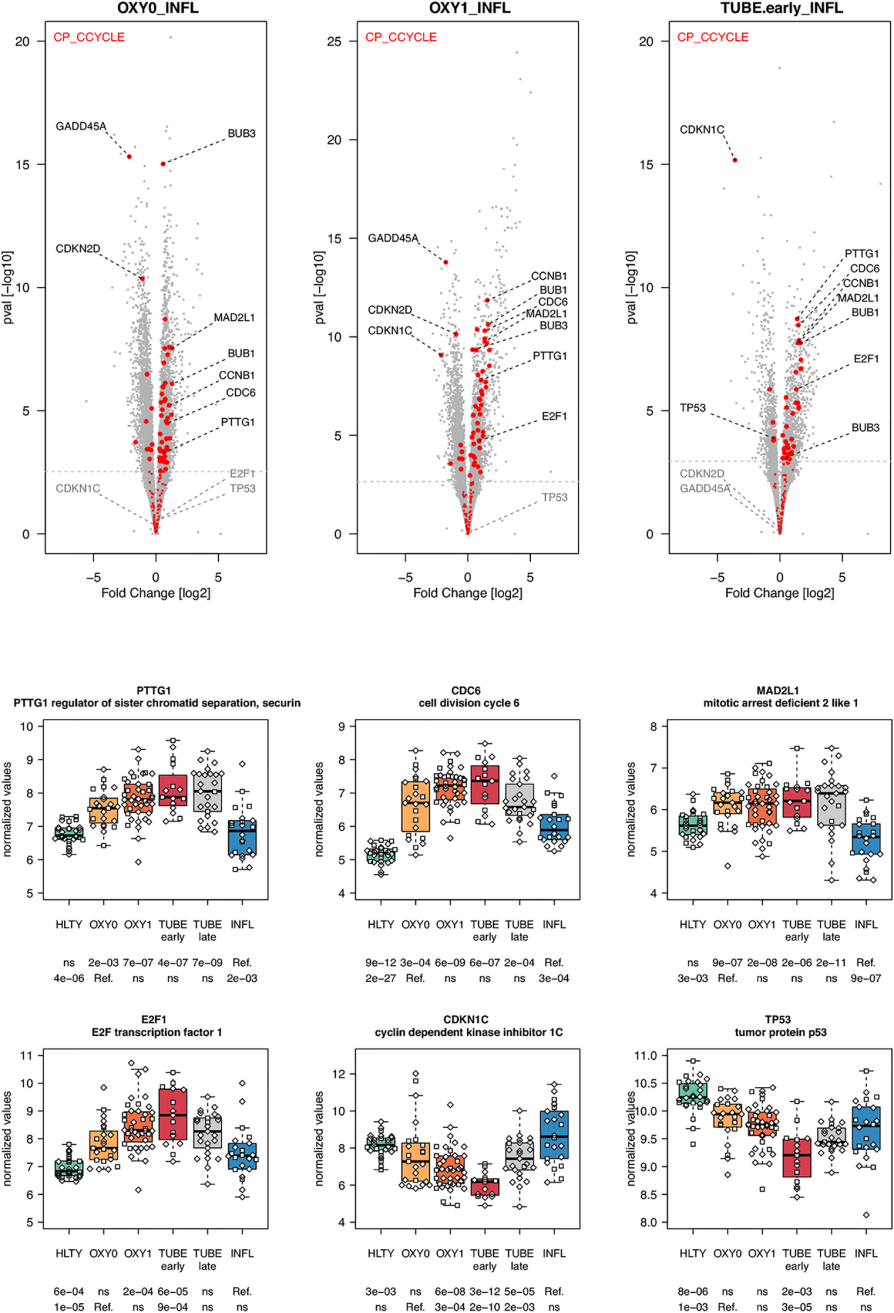
Differentially expressed genes (DEGs) involved in cell cycle. Upper panel: for each comparison indicated in the volcano plot header, the distribution of log2 fold changes and P-values of all detected genes is shown as gray dots. Red dots indicate genes involved in cell cycle. Genes’ sets are selected from different GO, REACTOME and/or KEGG pathways (see **Supplementary Table 2**). The dotted horizontal gray line indicates the limit under which –log10(P-value) becomes non significant once adjusted for multiple testing (adjusted P > 0.01). Lower panel shows detailed expression levels of selected genes involved in cell cycle in the different groups of patients. Groups used for comparisons include SARS-Cov-2 infected patients (no oxygen support [OXY0], oxygen support [OXY1], intubation with sampling within 7 days [TUBE early] or >7 days [TUBE late] after hospital admission), Influenza virus [INFL] infected patients and non-infected controls [HLTY]. Adjusted P-values of pairwise comparisons between a reference group (Ref.) and another group are indicated below the x axis. Ref. is taken as [INFL] and [OXY0] on the first and second line, respectively.

Genes involved in metabolism, such as oxidative phosphorylation, were over-expressed mainly in TUBE patients compared to all others (“hill” pattern, e.g. *NDUFV2, UQCRQ, ATP5PF, ACAT1, HIBCH and B4GALT2*, **Figure 11**). Genes involved in cell cycle, were over-expressed among COVID-19 compared to both HLTY controls and INFL, with a “hill” pattern (e.g. *PTTG1, CDC6, MAD2L1, E2F1*, **Figure 12**). Accordingly, regulator genes (e.g. *CDKN1C and TP53*, **Figure 12**) presented a “valley” pattern, with under expression in SARS-CoV-2 infected patients (mainly in TUBE early) compared to both HLTY and INFL patients. Interestingly, genes involved in cell cycle were largely over-expressed among COVID-19 patients compared to both healthy controls and patients infected with Influenza (**Data not shown**).

### Transcriptomics Signatures Associated With SARS-CoV-2 Infection and Its Severity

Finally, we investigated whether differential expressed gene signatures may be used to characterize SARS-CoV-2 infected patients (compared to the other) or severe versus non-severe presentation of COVID-19 (**Table 1**). We first identified genes significantly differentially expressed in all acute COVID-19 groups (OXY0, OXY1 and TUBE early) compared to INFL, and, to be even more stringent, to HLTY as well (**Supplementary Table 4**). A total of 209 and 6 genes were significantly over- and under-expressed, respectively, in all comparisons (adjusted P <=0.01). Among the most significantly over-expressed genes, a large number encoded immunoglobulins (N=67, e.g. JCHAIN, adj P=3.5E-09 for TUBE versus INFL) or were related to cell cycle (N=44, e.g. CDC6, adj P=6.3E-07 for the same comparison). The most significantly down-regulated gene was HDAC6 (adj P=9.7E-06 for TUBE versus INFL), an important regulator of transcription. We then identified markers of severe presentation of COVID-19 by selecting genes differentially expressed in TUBE-early compared to OXY0, OXY1, INFL and HLTY; to have an even more stringent selection, genes were selected if they were not differentially expressed in OXY0 compared to HLTY (**Supplementary Table 5**). A total of 163 and 112 genes were over- and under-expressed, respectively (adjusted P <=0.01). Among significantly over-expressed genes in all indicated comparisons, some encode blood-groups (e.g. GYPA, adj P=1.7E-10 in TUBE early *vs* OXY0, see above); among significantly under-expressed genes in all indicated comparisons, several were immune genes (e.g. IL2RB, adj P=8.2E-07 and IL10RA, adj P=1.6E-05, for TUBE early versus OXY0).

**Table 1:** Most significant genes signatures for SARS-CoV-2 infection (upper panel) and severe COVID-19 presentation (lower panel).

COVID-19 *vs* INFL and HLTY	OXY0 *vs* INFL		OXY1 *vs* INFL		TUBE early *vs* INFL		OXY0 *vs* HLTY		OXY1 *vs* HLTY		TUBE early *vs* HLTY	
	log2 FC	padj	log2 FC	padj	log2 FC	padj	log2 FC	padj	log2 FC	padj	log2 FC	padj
**Over-expressed (top-10)**												
CDC6	0.92	2.5E-04	1.37	5.8E-09	1.50	6.3E-07	2.71	1.8E-27	3.14	4.0E-47	3.28	1.9E-32
IGLV3-25	3.28	4.1E-10	4.19	7.1E-20	3.22	7.0E-08	4.71	1.6E-23	5.60	5.1E-43	4.66	2.9E-19
JCHAIN	2.43	1.6E-09	2.87	7.3E-16	2.72	3.5E-09	3.67	8.1E-24	4.10	5.2E-39	3.97	8.9E-23
RRM2	1.08	1.4E-04	1.57	4.1E-09	1.55	5.3E-06	2.77	8.1E-24	3.22	6.3E-42	3.23	3.4E-26
IGLV3-19	2.09	3.0E-05	3.93	6.1E-17	4.10	1.1E-11	3.44	1.0E-12	5.22	2.6E-36	5.40	4.4E-24
IGLV3-1	2.35	3.2E-07	3.91	6.2E-21	2.65	6.5E-07	3.38	2.9E-15	4.91	1.3E-40	3.68	2.9E-15
SDC1	2.78	1.5E-07	3.51	2.2E-13	2.83	3.0E-06	4.78	3.5E-21	5.48	2.6E-35	4.85	1.5E-18
IGLV3-10	2.99	4.9E-08	3.86	6.9E-15	3.36	1.5E-07	4.50	9.6E-19	5.33	3.6E-34	4.86	3.3E-18
IGHV1-24	3.52	1.9E-08	5.03	2.2E-19	3.53	6.9E-07	4.49	8.1E-15	5.97	2.3E-33	4.52	4.5E-13
BHLHA15	2.04	2.1E-06	2.81	1.5E-12	2.38	1.9E-06	3.71	6.8E-18	4.44	5.2E-32	4.05	2.4E-18
**Under-expressed (N=6)**												
HDAC6	-0.36	2.6E-05	-0.30	1.2E-04	-0.46	9.7E-06	-0.45	4.0E-08	-0.39	3.0E-08	-0.56	1.1E-09
UNK	-0.26	4.9E-03	-0.28	1.2E-03	-0.59	8.0E-08	-0.26	3.1E-03	-0.27	2.3E-04	-0.60	3.3E-10
ZNF384	-0.40	7.6E-08	-0.22	1.2E-03	-0.30	4.6E-04	-0.34	5.2E-07	-0.16	5.8E-03	-0.26	6.0E-04
TGFBI	-0.63	7.2E-03	-0.67	2.2E-03	-1.47	6.3E-07	-0.63	5.1E-03	-0.69	5.3E-04	-1.49	7.5E-09
UBAP2L	-0.31	1.7E-03	-0.25	6.3E-03	-0.42	2.2E-04	-0.28	3.2E-03	-0.23	7.0E-03	-0.41	5.1E-05
DIAPH1	-0.41	1.2E-03	-0.33	4.7E-03	-0.43	4.1E-03	-0.40	7.8E-04	-0.33	1.5E-03	-0.46	6.4E-04
***TUBE early vs***	**TUBE early *vs* OXY0**		**TUBE early *vs* OXY1**		**TUBE early *vs* INFL**		**TUBE early *vs* HLTY**				**Control : OXY0 *vs* HLTY**	
***OXY0, OXY1, INFL and HLTY***	**log2 FC**	**padj**	**log2 FC**	**padj**	**log2 FC**	**padj**	**log2 FC**	**padj**			**log2 FC**	**padj**
**Over-expressed (top-10)**												
CATIP-AS1	2.99	2.1E-12	2.68	1.1E-11	1.82	1.3E-05	2.95	2.5E-15			-0.03	9.3E-01
MTCO1P12	3.77	2.2E-11	0.13	2.9E-06	8.05	1.7E-11	0.26	5.4E-11			-0.28	3.0E-01
GYPA	3.89	5.2E-10	3.25	1.9E-08	3.16	4.4E-07	4.22	3.0E-14			0.12	6.4E-01
CDK5RAP2	2.35	1.2E-09	2.07	1.5E-08	1.15	2.1E-03	2.74	1.5E-15			0.25	3.2E-01
MUC1	1.63	2.2E-08	1.52	3.5E-08	1.25	1.9E-05	1.98	3.1E-14			0.26	2.4E-01
NOL3	1.24	1.2E-09	0.54	2.7E-03	0.55	6.1E-03	1.67	1.0E-19			0.37	2.5E-02
CA1	4.82	2.1E-12	2.76	5.5E-06	2.47	1.3E-04	3.97	1.4E-11			-0.35	2.0E-01
EXTL3-AS1	1.76	5.9E-10	1.46	4.5E-08	0.83	2.4E-03	1.82	4.6E-13			0.04	8.7E-01
SMARCD3	1.50	2.0E-08	0.79	8.9E-04	0.94	4.1E-04	2.07	4.6E-18			0.46	2.2E-02
SERINC2	2.30	6.8E-12	1.55	6.2E-07	1.02	1.5E-03	2.00	8.8E-12			-0.21	3.5E-01
**Under-expressed (top-10)**												
ENSG00000186076.5	-0.04	1.7E-10	-0.02	1.5E-11	-0.02	1.2E-19	-0.16	2.7E-18			-0.06	8.0E-02
EVL	-0.98	1.3E-07	-0.74	2.3E-05	-0.80	4.7E-07	-1.39	1.6E-16			-0.35	2.3E-02
DCTD	-0.62	2.9E-08	-0.42	7.4E-05	-0.43	4.5E-06	-0.82	7.5E-17			-0.19	4.1E-02
IL2RB	-1.22	8.2E-07	-1.06	6.6E-06	-1.10	2.1E-07	-1.81	7.4E-16			-0.49	1.2E-02
SPN	-1.02	1.4E-07	-0.76	2.5E-05	-0.94	3.2E-08	-1.33	1.7E-14			-0.26	9.8E-02
CDKN1C	-3.26	1.7E-10	-1.15	4.2E-03	-3.59	6.8E-16	-2.30	1.4E-07			0.66	4.1E-02
RUNX3	-1.03	9.7E-07	-0.92	4.5E-06	-1.01	3.0E-08	-1.44	7.2E-14			-0.32	5.5E-02
SCART1	-1.54	7.7E-08	-1.17	1.7E-05	-0.78	4.9E-04	-1.99	1.1E-14			-0.35	8.9E-02
IL10RA	-0.61	1.6E-05	-0.52	1.3E-04	-0.79	2.4E-10	-0.93	3.8E-13			-0.29	1.5E-02
ZNF703	-2.03	1.7E-07	-1.26	3.1E-04	-2.22	6.0E-11	-2.22	1.2E-10			-0.11	6.7E-01

To identify expression signature for SARS-CoV-2 infection and severe COVID-19, genes whose expression was significantly different (Padj <0.01) in all of the indicated comparisons were selected; ranking was obtained by multiplication of adjusted P values for all relevant comparisons. For severity of COVID-19, selected genes also had to be not significantly differentially expressed in OXY0 compared to HLTY (negative control).

## Discussion

In this study, we describe major differences in the blood transcriptomic profiles of COVID-19 patients compared to subjects affected with influenza and between COVID-19 of different level of severity. One of the most striking features was the low expression of ISGs in COVID-19 compared to INFL patients. ISGs represent a group of genes transcriptionally activated by IFN signaling, which is essential for both innate and adaptive antiviral immunity against a wide range of pathogens (28, 29). ISGs restrict viral replication and spread by inhibiting key steps of viral life cycle (30, 31). The down-regulation of ISGs is consistent with numerous immune escape mechanisms developed by CoVs to enhance their replication and/or survival in the host (32, 33). Specifically, CoVs have developed mechanisms to evade the detection by PPRs including MDA5 [NSP15 encoded by several CoVs (34, 35)], RIG-I/RLRs[(SARS-CoV-1 N-protein (36)], and PKR [MERS-CoV NS4a (37, 38)], their signaling molecules, such as MAVS/TOM70 [SARS-CoV-2 ORF9b (39, 40)] and TBK1 [MERS-CoV ORF4b (41)], as well as the transcription factors IRF3 [SARS-CoV-1 ORF8b (42)] and NF-kB [MERS-CoV ORF4b (43)]. Similarly, CoVs inhibit signaling molecules downstream of IFN production, including IFNAR1 [SARS-CoV-1 3a protein (44)] and STAT1 [SARS-CoV-1 ORF6 (45)]. The under-expression of genes involved in viral detection (such as TLRS or RLRs) observed in this study may be due to additional mechanisms, including genetic variation in the host (46) or virus-dependent transcriptional regulation. Furthermore, conditions associated with a severe presentation of SARS-CoV-2, such as advanced age, diabetes and cancer (47) are characterized by an impairment in type I and III interferon responses (48).

While ISGs expression levels were lower in COVID-19 compared to INFL patients, expression levels of IFNs themselves, were not detectable in the peripheral blood, similarly to other transcriptomic studies (49). In a cellular model of SARS-CoV-2 infection, the production of type I and III IFNs was relatively low at low multiplicity of infection (MOI), but not at higher MOI, suggesting that IFN induction is initially limited, but can increase once a high level of viral replication is achieved (50). Conversely, in an animal model of SARS-CoV, robust viral replication was associated with delayed IFN type I responses, which subsequently leads to increased inflammation and lung damage (51). In cellular models, MERS-CoV induced a delayed and attenuated IFN type I response compared to those induced by human coronavirus 229E (HCoV-229E) (52). Similarly, SARS-CoV-2 patients requiring invasive mechanical ventilation presented the highest amount of IFNα at days 8 to 10 of symptom onset (53). Recent studies have also suggested that antibodies directed to IFN may be responsible for delayed antiviral immunity (54). Single-cell RNA sequencing revealed a suppression of IFN signaling among COVID-19 patients compared to Influenza patients (55). In addition, the same technique highlighted the key role of type I IFN in exacerbating inflammation in severe COVID-19 patient compared to what observed in healthy donors and in severe influenza patients (56). Altogether, these observations indicate that unfavorable outcomes in COVID-19 may result from the delayed or impaired production of type I and III IFNs, or their subsequent inhibition by antibodies, with compromised virus control and prolonged activation of inflammatory cytokines. This is in agreement with previously reported data from Galani et al. (57).

Natural killer (NK) cells and effector T cells both target infected cells exposing viral peptides through the MHC I. As reported for the SARS (58), the amount of both NK cells and T cells was highly decreased in SARS-CoV-2 patients (59–61). In addition, the frequencies of NK cells expressing *CD16* and/or KIRs were reduced in the blood of patients infected with SARS-CoV-2 (61). Consistent with this observation, we found reduced expression of *CD16*, KIRs and *TBX21* in SARS-CoV-2 infected patients compared to INFL patients. These data suggest that the former patients harbor immature NK cells, which may not be able to migrate towards infected tissues. Furthermore, we observed that SARS-CoV-2 infected patients had lower expression of genes involved in NK cell cytotoxicity such as perforin (*PRF1*), granulysin B (*GNLY*) and CD107a (*LAMP1*), an observation which is consistent with studies analyzing intracellular expression patterns of NK cells from COVID-19 patients (59, 62). Immune checkpoints such as NKG2A/KLRC1, a co-receptor of CD94/KLRD1 which interacts with HLA-E, as well as inhibitory receptors such as LAG3 were upregulated in SARS-CoV-2 infected patients compared to healthy individuals (59). In our study, *NKG2A/KLRC1* and *LAG3* were not overexpressed and *CD94/KLRD1* was even under-expressed among TUBE EARLY patients compared to both HTLY and INFL, suggesting that the level of inhibition may depend on the severity level of the disease. Genes involved in neutrophil degranulation and/or other neutrophil functions had increased expression levels in patients with severe manifestations of COVID-19, such as *S100A8*/*9* and *S100A12*, encoding calprotectin and calgranulin, respectively, as recently reported (63). However, such responses may be a general feature of severe lung infection rather than a COVID-19-specific feature (64, 65).

Another relevant feature of transcriptional profiles observed in this study is the low expression of MHC I and MHC II encoding genes among patients infected with SARS-CoV-2 compared to INFL patients. Down-regulation of genes involved in antigen presentation through both the MHC I and MHC II have been reported in lung epithelial cells infected with MERS-CoV, but not with SARS-CoV-1, highlighting important differences in viral escape mechanisms among different CoVs (66). Inhibition of both MHC I [reviewed in (67)] and MHC II [reviewed in (68, 69)] represents well-known mechanisms developed by viruses to escape immune response. Specifically, Herpesviridae can prevent MHC I mediated antigen presentation by interfering with the generation of antigenic peptides by the proteasome (EBV), preventing their transport across the endoplasmic reticulum towards the peptide-loading complex (HSV, CMV) and/or by avoiding adequate presentation of this complex on cell surfaces [CMV, KHSV, reviewed in (67)]. Interestingly, our study shows that both transporters associated with antigen processing (TAP) 1 and 2 had lower expression levels in SARS-CoV-2 patients compared to patients with influenza, revealing a potential immune escape mechanism for SARS-CoV-2. In addition, it was recently proposed that the protein encoded by SARS-CoV-2 open reading frame (ORF) 8 can directly interact with MHC I molecules and significantly down-regulate their surface expression on various cell types (70). Since ORF8 is among the viral sequences having the less homology among CoVs, differences in ORF8 may explain differential capability among CoVs to inhibit antigen presentation through MHC I (69). Altogether, our results suggest that SARS-CoV-2 restrains antigen presentation and T cell mediated immune responses.

Activation of CD4+ T cells by interactions with peptides bound to MHC II is a crucial step in clearance of most pathogens. Many viruses have developed ways of blocking antigen presentation, although fewer mechanisms or viral interference have been described for MHC II compared to MHC I (69). Specifically, viruses can target MHC class II transactivator (CIITA), a key molecule in the control of MHC II proteins transcription (EBV, KHSV, HIV), the invariant chain protein (Ii/CD74), which co-assembles with MHC II αβ heterodimers in the endosome (CMV, EBV), its associated peptide (CLIP; HCV), as well as MHC II proteins themselves, taken either alone (CMV, EBV) or during their interaction with the T cell receptor [EBV; reviewed in (69)]. Accordingly, we observed that genes encoding proteins involved in MHC II activation (including *CTSB, Ii/CD74* and *CLIP*) had low expression levels in patients infected with SARS-CoV-2 compared to INFL patients, revealing another potential immune escape mechanism for SARS-CoV-2.

T cells play a crucial role in antiviral immunity and were reported to be decreased in SARS-CoV infection (59–61) [reviewed in (71)] but their phenotypical and functional alterations due to COVID-19 are poorly characterized. While CD4 T helper cells contribute to B cell activation and subsequent antibody production, CD8 T cells kill infected cells and reduce viral burden. We observed reduced expression of *CD4* in all severity types of COVID-19 patients compared to HLTY and INFL patients; in contrast, *CD8* was under-expressed solely among TUBE early patients compared to HTLY, with levels similar to those of INFL patients. Altogether, these data suggest a more specific burden of SARS-CoV-2 on CD4 T cells than over CD8 T cells. Persistence of high expression levels of CD8 among OXY0 and OXY1 patients may result from the increased expression of IRF4 which is known to enhances CD8 expression (72), and that showed an expression pattern contrasting that of the other ISGs. Conversely, reduced expression of CD8 T cells in TUBE early patients may result from CD8 T cell infiltration of the lungs, as reported by others (73).

When analyzing the differential expression of memory markers in our dataset, we were not able to characterize specific T cell subsets, a finding that may be a consequence of the experimental design of the study (as a whole blood *vs.* single cells were considered) or of the lack of a well-characterized T cell memory pattern in the patients themselves. When considering the differential expression of T cell markers of activation, we noted a significantly enhanced expression of *CD38, Ki-67/MK67* and *CD44* [which is consistent with the observation by Braun et al. (7)], but not of *HLA-DR* (7, 74), *CD69* (75) and *CD25* (76). Furthermore, we noted an over-expression of *CSF1/GM-CSF* correlated with COVID-19 severity, a finding that may lead to increased inflammation in severe patients, as suggested by Zhou et al. (77). Interestingly, GM-CSF+ CD4 T cells have been associated with inflammation in autoimmune diseases (78). We also observed a decreased expression of *IL2RB/AKA CD122*, which correlated with severity and may result in T cell deregulation (79). In contrast to previous studies (80), the exhaustion marker PD-1 was not over-expressed, but rather under-expressed, among TUBE early patients, which may reflect a defect in TCR signaling and/or in effector T cells activation. Altogether, the expression of T cell markers of memory and activation was contrasted, suggesting an overall dysregulation of T cell activity (81).

Humoral immune responses also play an important role in the clearance of SARS-CoV-2 and the establishment of an immunological memory (82). SARS-CoV-2 elicits a strong B cell response with previously described kinetics (83, 84). Our data reveal a dramatic increase in the expression of immunoglobulin encoding genes of most classes (*IGHM, IGHA, IGHG*) in COVID-19 compared to HLTY and INFL subjects, with a peak among OXY1 and/or TUBE-early patients. There was also an increased expression of J-CHAIN, which contribute to polymer formation of secreted IgM and IgA, thereby enhancing antigen avidity and viral binding (85). These findings are consistent with the over-expression of genes involved in B cell differentiation and antibody class switching (*CD40L, UNG, ICOS, BAFFR, CD19 and TACI*) in OXY0 and OXY1 compared to INFL patients, with the exception of the Bruton’s tyrosine kinase (*BTK*). Paradoxically, antibodies can play a deleterious role in SARS-CoV-2 pathogenesis. Opsonization of anti-spike antibodies allow SARS-CoV entering non-ACE2-expressing cells, which harbor Fc-g-RIIA, a receptor that happened to be over-expressed in TUBE early patients (86, 87). Excessive antibody production together with presentation of host proteins resulting from prolonged tissue destruction may enhance auto-reactive responses contributing to disease severity (88, 89). In addition, antibodies targeting SARS-CoV epitopes were shown to cross-react with cytokines, such as IL-11 (90, 91); recent studies also revealed presence of antibodies targeting type-I IFN in COVID-19 patients (54). Single cells transcriptome of peripheral blood mononuclear cells in both COVID-19 and Influenza-infected patients revealed an enrichment of plasma cells in COVID-19 patients which has been correlated with the production of multiple protective neutralizing antibodies (92). Altogether, these data suggest an important activation of B cells with increased antibody production in COVID-19 compared to INFL patients. Further studies are needed to determine whether this strong response results from specific features of the virus, from the absence of previous exposure to its antigens, and to which extent they contribute to an adverse outcome.

The complement and coagulation systems are increasingly reported to play a relevant role in the pathogenesis of COVID-19 (93). Several studies have revealed that SARS-CoV-2 induced the activation of several complement pathways (94, 95). Complement system is based on the activation and cleavage of proteins that cannot be addressed with transcriptomic data. Interestingly, we observed an increased expression of complement component-encoding genes in both COVID-19 and INFL compared to HLTY, with patterns not strikingly different in patients infected with both viruses. Some genes were slightly under-expressed among OXY0 compared to INFL (*C1qrs, C2, C5*); in contrast, the complement component *C3* was over-expressed in TUBE early patients compared to INFL patients. Activated C3 can exacerbate SARS-CoV-associated acute respiratory distress syndrome (ARDS) (95). This suggests that, while the activation of the complement exists in both COVID-19 and in INFL, its persistence over a longer period of time in COVID-19 may contribute to inflammation, tissue damage, coagulation and neutrophil activation, leading to organ injury. This is consistent with the significant increase in the expression of neutrophil degranulation-associated genes observed among TUBE early compared to INFL patients. There was no definite profile in the expression of genes involved in the coagulation cascade among COVID-19 patients compared to INFL. In contrast, we observed a strong over-expression of blood group encoding genes in TUBE early/late patients compared to the other groups. Blood groups have been shown to change the immune response to infections (96). Some of these genes encode proteins involved in the pathogenesis of viral infections, such as *GYPA*, which codes for glycophorin A, a protein considered as the major receptor for different viruses on the red blood cell surface (97–99). Others, such as those of the Knops blood group system, play a role in the activation and/or modulation of the complement cascade (100, 101). However, it remains unclear whether the overexpression of blood-group genes is related to the pathogenesis of COVID-19 or reflects a general activation of inflammation and/or hematopoiesis.

Increasing evidence reveals important interactions between metabolic and immune functions (102). For instance, aerobic glycolysis is induced during the activation of numerous immune cells, such as M1 macrophages (103), dendritic cells (104), T cells (105), B cell (106, 107) and NK cells (108). In addition, oxidative phosphorylation plays an important role in the activation of M2 macrophages (109, 110) and in the expression of transcription factors that are essential for T and B cells. This is consistent with the over-expression of genes involved in carbohydrate metabolic pathways in OXY1 compared to INFL patients. Differential expression of genes involved in the metabolism may also contribute to disease conditions (111).

Viruses influence host cells to create a favorable environment for their replication (112, 113). SARS-CoV non-structural proteins 3b and 7a were shown to induce a G0/G1 phase arrest in infected cells (114, 115). This phenomenon is probably limited to the site of infection, i.e. respiratory epithelial cells, which are infected in COVID-19. In contrast, the analysis of whole blood transcriptome reveals significant overexpression of genes involved in cell cycle, which probably reflects the activation of immune cells distant from the site of infection. This over-expression was present in all types of COVID-19 patients (OXY0, OXY1 and TUBE) compared to both HTLY and INFL. Further studies are needed to understand whether cell-cycle activation is a specific feature of SARS-CoV-2 infection or a component of the resulting inflammatory response.

To date, biomarkers of COVID-19 and/or COVID-19 severity mostly included clinical and inflammatory characteristics (116). To our knowledge, no study allowed for a large scale comparison of gene expression between COVID-19 and another viral infection. By using blood from patients with different severity levels of COVID-19 as well as INFL and HLTY, we identified and/or confirmed a number of very significant associations. In particular, genes encoding immunoglobulins and those related to cell cycle appear as a hallmark of COVID-19, as they were significantly independently upregulated in each COVID-19 severity group (OXY0, OXY1 and TUBE early) compared to both INFL and HLTY. Furthermore, a number of genes were significantly over (e.g. genes encoding PRR, ISG, or related to macrophages functions, degranulation of neutrophils, blood groups, complement, metabolism and/or oxidative phosphorylation) or under-expressed (e.g. genes related to MHC II, T-cell function or encoding immunoglobulins) among TUBE early patients compared to the other severity group (OXY0 and OXY1), thereby providing a potential basis to predict COVID-19 outcome. However, new studies including samples taken at different time-points of COVID-19 as well as patients with infections due to other pathogens will be needed to confirm these findings and/or establish reliable diagnostic tools to predict COVID-19 outcome.

Like other transcriptomic studies, this work has several limitations. The number of patients included in the different groups was limited, a factor that may have restricted the number of DEG reported. Samples were taken from whole blood and do not necessarily reflect gene expression patterns in clinically affected organs and/or individual cells. The sequencing depth may have restricted differential detection of less abundantly expressed genes. Finally, the samples were issued from a single cohort of patients, and thus validation from other cohorts would be useful. This study provides a comprehensive overview of the immune response among patients with different severity levels of COVID-19. These include a dramatic decrease in IFN responses, a reduced cytotoxicity activation in NK cells, an increased degranulation of neutrophils, a dysregulation of T cells, a dramatic increase in B cell function and immunoglobulin production, as well as an important over-expression of genes involved in metabolism and cell cycle. This study opens the way to further investigations aimed at elucidating the molecular mechanisms that underlay these observations. This study also suggests that it may be possible to identify a signature which could be useful to identify early patients at risk of adverse outcome.

## Data Availability Statement

Dataset containing, raw and normalized gene counts and samples description is available on http://dx.doi.org/10.17632/8wxhhykfnh.2. Unprocessed raw data (fastq files) cannot be shared due to ethical and legal limitations. An interactive website interface is made available to the readers, in which global and detailed gene profiles can be visualized (https://bix.unil.ch/covid/).

## Ethics Statement

The studies involving human participants were reviewed and approved by Cantonal Ethics Committee of the state of Vaud (Swissethics 2020-01108). The patients/participants provided their written informed consent to participate in this study.

## Author Contributions

Conceptualization: SB, NB, PYB. Methodology: SB, NG, JL, MD, NB, PYB. Bioinformatic analysis: NG, JL, RL, LG, MD. Acquisition, Analysis or interpretation of data: SB, TB, FL, MP-O, LD, OM, JT, PV, JLP, MO, OH, VE, CV, FC, NB, PYB. Investigation: SB, NB, MQ, CR, PYB. Writing original draft: PYB, NB, SB, NG, JL. Writing-Review & Editing: ALL. Obtained Funding: PYB, CR, Administrative, technical, or material support: SB, TB, MP-O, LD. Supervision: PYB. All authors contributed to the article and approved the submitted version.

## Funding

P-YB is supported by the Swiss National Science Foundation (31CA30_196036, 33IC30_179636 and 314730_192616), the Leenaards Foundation, the Santos-Suarez Foundation as well as grants allocated by Carigest. NB is supported by the Leenaards Foundation. CR is supported by the Swiss National Science Foundation (31CA30_196036 and 31003A_176097).

## Conflict of Interest

The authors declare that the research was conducted in the absence of any commercial or financial relationships that could be construed as a potential conflict of interest.

## Supplementary Material

Supplementary Table 1 Characteristics of patients infected with SARS-CoV-2 or Influenza virus and heathy controls.

Supplementary Table 2 Composition of genes sets for main figures.

Supplementary Table 3 Composition of genes sets for supplementary figures.

Supplementary Table 4 Differentially expressed genes as markers of COVID-19.

Supplementary Table 5 Differentially expressed genes as markers of COVID-19 severity.

Supplementary Figure 1 Quality control.

Supplementary Figure 2 Unsupervised analysis: differentially expressed genes in KEGG pathways. MET stands for metabolism, GIP stands for Genetic Information Processing, EIP stands for Environmental Information Processing, CP stands for Cellular Processes, OS stands for Organismal Systems and HD stands for Human Diseases.

Supplementary Figure 3 Interpretation of boxplots (“ascending”, “descending”, “hill” and “valley” pattern). Expression patterns across the different groups were regarded as “**ascending**” or “**descending**” when median expression levels progressively increased, or decreased, respectively, from HLTY to OXY0, OXY1, TUBE-early, and INFL. Conversely, patterns were qualified to have a “**hill**” or “**valley**” pattern, when peak and lowest median expression levels were seen in COVID-19 (OXY1 or TUBE-early), and the lowest and higher in both HLTY and INFL, respectively. Because TUBE-late often represented a convalescent status, their gene expression levels were ignored in this pattern qualification.

Supplementary Figure 4 KEGG map colored with pathview (117) “Influenza A” pathway (hsa05164). Pathways involved in the detection of Influenza A overlap with those involved in the detection of other RNA viruses including SARS-CoV-2. Significantly DEGs (adj. P-Value < 0.01) are colored based on their log2 fold change. Left panel: boxes are separated into 3 slots, for OXY0 versus INFL, OXY1 versus INFL and TUBE-early versus INFL. Right panel: boxes are separated into 2 slots, for INFL versus HTLY and TUBE_early versus HLTY.

Supplementary Figure 5 KEGG map colored with pathview (117) **“**Cytokines-cytokine receptor interaction” (hsa04060). Significantly DEGs (adj. P-Value < 0.01) are colored based on their log2 fold change. Left panel: boxes are separated into 3 slots, for OXY0 versus INFL, OXY1 versus INFL and TUBE-early versus INFL. Right panel: boxes are separated into 2 slots, for INFL versus HTLY and TUBE_early versus HLTY.

Supplementary Figure 6 KEGG map colored with pathview (117) “Natural killer cell mediated toxicity” (hsa04650). Significantly DEGs (adj. P-Value < 0.01) are colored based on their log2 fold change. Left panel: boxes are separated into 3 slots, for OXY0 versus INFL, OXY1 versus INFL and TUBE-early versus INFL. Right panel: boxes are separated into 2 slots, for INFL versus HTLY and TUBE_early versus HLTY.

Supplementary Figure 7 KEGG map colored with pathview (117) “Antigen processing and presentation” (hsa04612). Significantly DEGs (adj. P-Value < 0.01) are colored based on their log2 fold change. Left panel: boxes are separated into 3 slots, for OXY0 versus INFL, OXY1 versus INFL and TUBE-early versus INFL. Right panel: boxes are separated into 2 slots, for INFL versus HTLY and TUBE_early versus HLTY.

Supplementary Figure 8 Box plots for selected genes involved in different T cells functions.

Supplementary Figure 9 KEGG map colored with pathview (117) “T cell receptor signaling pathway” (hsa04660). Significantly DEGs (adj. P-Value < 0.01) are colored based on their log2 fold change. Left panel: boxes are separated into 3 slots, for OXY0 versus INFL, OXY1 versus INFL and TUBE-early versus INFL. Right panel: boxes are separated into 2 slots, for INFL versus HTLY and TUBE_early versus HLTY.

Supplementary Figure 10 KEGG map colored with pathview (117) “Th1 and Th2 differentiation” (hsa04658). Significantly DEGs (adj. P-Value < 0.01) are colored based on their log2 fold change. Upper panel: boxes are separated into 3 slots, for OXY0 versus INFL, OXY1 versus INFL and TUBE-early versus INFL. Lower panel: boxes are separated into 2 slots, for INFL versus HTLY and TUBE_early versus HLTY.

Supplementary Figure 11 KEGG map colored with pathview (117) “Th17 differentiation” (hsa04659). Significantly DEGs (adj. P-Value < 0.01) are colored based on their log2 fold change. Upper panel: boxes are separated into 3 slots, for OXY0 versus INFL, OXY1 versus INFL and TUBE-early versus INFL. Lower panel: boxes are separated into 2 slots, for INFL versus HTLY and TUBE_early versus HLTY.

Supplementary Figure 12 KEGG map colored with pathview (117) "B cell receptor signaling pathway" (hsa04662). Significantly DEGs (adj. P-Value < 0.01) are colored based on their log2 fold change. Upper panel: boxes are separated into 3 slots, for OXY0 versus INFL, OXY1 versus INFL and TUBE-early versus INFL. Lower panel: boxes are separated into 2 slots, for INFL versus HTLY and TUBE_early versus HLTY.

Supplementary Figure 13 Box plots for selected genes involved in B cells functions/maturation/regulation.

Supplementary Figure 14 KEGG map colored with pathview (117) "Complement and coagulation cascades" (hsa04659). Significantly DEGs (adj. P-Value < 0.01) are colored based on their log2 fold change. Left panel: boxes are separated into 3 slots, for OXY0 versus INFL, OXY1 versus INFL and TUBE-early versus INFL. Right panel: boxes are separated into 2 slots, for INFL versus HTLY and TUBE_early versus HLTY.

Supplementary Figure 15 Boxplots plots for complement, coagulation and blood groups.
