# Supplementary material for: Transcriptomic Signature Differences Between SARS-CoV-2 and Influenza Virus Infected Patients
Source: Front Immunol. 2021 May 31;12:666163. doi: 10.3389/fimmu.2021.666163 (PMC8202013; doi:10.3389/fimmu.2021.666163)
Supplement: Supplementary Table 1 — Characteristics of patients infected with SARS-CoV-2 or Influenza virus and heathy controls. [file Table_1.docx]

**Supplementary Table 1**

| **Characteristics (N, %)** | **HLTY**  **(n=27)** | **SARS-CoV-2 infection** | | | |  | **INFL**  **(N=22)^a^** |  |  |  |  |
| --- | --- | --- | --- | --- | --- | --- | --- | --- | --- | --- | --- |
|  |  | **OXY0**  **(N=23)** | **OXY1**  **(N=40)** | **Intubated (sample)** | | ***P value***  ***overall***  ***SARS-***  ***CoV2*** |  |  | ***P value INFL*** | | |
|  |  |  |  | **TUBE early**  **(N=15)** | **TUBE late**  **(N=25)** |  |  |  | ***vs***  ***OXY0*** | ***vs***  ***OXY1*** | ***vs TUBE***  ***early*** |
|  |  |  |  |  |  |  |  |  |  |  |  |
| Demographics |  |  |  |  |  |  |  |  |  |  |  |
| Mean age (years, range) | 63 (46-84) | 56 (23-92) | 60 (30-89) | 64 (23-82) | 63 (46-82) | *0.3* | 73 (31-96) |  | ***0.001*** | ***<0.001*** | ***0.05*** |
| Male sex | 17 (63%) | 14 (61%) | 27 (68%) | 8 (53%) | 23 (92%) | ***0.02*** | 13 (59%) |  | *1.0* | *0.6* | *0.7* |
|  |  |  |  |  |  |  |  |  |  |  |  |
| Comorbidities |  |  |  |  |  |  |  |  |  |  |  |
| Hypertension | 5 (19%) | 10 (43%) | 20 (50%) | 7 (47%) | 12 (48%) | *1.0* | 4 (18%) |  | *0.1* | ***0.02*** | *0.08* |
| Cardiovascular disease | 2 (7%) | 5 (22%) | 6 (15%) | 3 (20%) | 5 (20%) | *0.9* | 5 (23%) |  | *1.0* | *0.5* | *1.0* |
| Dyslipidemia | 0 (0%) | 4 (17%) | 12 (30%) | 2 (13%) | 6 (24%) | *0.6* | 1 (5%) |  | *0.3* | ***0.02*** | *0.6* |
| Neurological disorder | 1 (4%) | 0 (0%) | 1 (3%) | 2 (13%) | 2 (8%) | *0.2* | 3 (14%) |  | *0.1* | *1.0* | *1.0* |
| Respiratory disease | 1 (4%) | 4 (17%) | 5 (13%) | 2 (13%) | 3 (12%) | *1.0* | 6 (27%) |  | *0.5* | *0.2* | *0.4* |
| Gastrointestinal disease | 1 (4%) | 4 (17%) | 7 (17%) | 1 (7%) | 9 (36%) | *0.2* | 0 (0%) |  | *0.1* | ***0.04*** | *0.4* |
| Chronic kidney disease | 2 (7%) | 4 (17%) | 5 (13%) | 2 (13%) | 1 (4%) | *0.5* | 2 (9%) |  | *0.7* | *1.0* | *1.0* |
| Diabetes mellitus | 0 (0%) | 4 (17%) | 11 (28%) | 1 (7%) | 5 (20%) | *0.4* | 2 (9%) |  | *0.7* | *0.1* | *1.0* |
| Malignancy | 0 (0%) | 2 (9%) | 3 (7%) | 2 (13%) | 1 (4%) | *0.8* | 2 (9%) |  | *1.0* | *1.0* | *1.0* |
| HIV/AIDS | 0 (0%) | 0 (0%) | 1 (3%) | 0 (0%) | 2 (8%) | *0.5* | 0 (0%) |  | *1.0* | *1.0* | *NA* |
| Mean BMI (kg/m2, range) | *NA* | 27 (22-33)^c^ | 29 (22-44)^d^ | 30 (22-48) | 28 (19-39)^b^ | *0.2* | 27 (18-46)^c^ |  | *1.0* | *0.1* | *0.2* |
|  |  |  |  |  |  |  |  |  |  |  |  |
| Baseline symptoms |  |  |  |  |  |  |  |  |  |  |  |
| Fever | *NA* | 8 (35%) | 29 (72%) | 12 (80%) | 19 (76%) | ***0.006*** | 16 (73%) |  | ***0.02*** | *1.0* | *0.7* |
| Cough | *NA* | 8 (35%) | 32 (80%) | 14 (93%) | 16 (64%) | ***<0.001*** | 21 (95%) |  | ***<0.001*** | *0.1* | *1.0* |
| Dyspnea | *NA* | 7 (30%) | 32 (80%) | 12 (80%) | 19 (76%) | ***<0.001*** | 10 (45%) |  | *0.4* | ***0.01*** | ***0.05*** |
| Confusion | *NA* | 0 (0%) | 2 (5%) | 1 (7%) | 1 (4%) | *0.8* | 2 (9%) |  | *0.2* | *0.6* | *1.0* |
| Fatigue | *NA* | 9 (39%) | 28 (70%) | 9 (60%) | 11 (44%) | *0.06* | 20 (91%) |  | ***<0.001*** | *0.1* | ***0.04*** |
| Symptoms before admission  (mean days, range) | *NA* | 9.2 (2-30) | 8.9 (2-18) | 7.9 (4-14) | 7.1 (3-15) | *0.3* | 6.9 (2-20) |  | *0.2* | *0.1* | *0.5* |
|  |  |  |  |  |  |  |  |  |  |  |  |
| Baseline signs (mean, range) |  |  |  |  |  |  |  |  |  |  |  |
| Temperature (^°^C) | *NA* | 37.6 (36-40) | 38.4 (36-40) | 38.9 (38-40) | 38.4 (36-40)^e^ | ***<0.001*** | 38.0 (35-40) |  | *0.1* | *0.2* | *0.06* |
| Systolic blood pressure  (mmHg) | *NA* | 125 (103-147) | 121 (80-153) | 112 (84-162) | 107 (82-131)^c^ | ***0.001*** | 132 (93-162) |  | *0.1* | ***0.02*** | ***0.005*** |
| Heart rate | *NA* | 82 (60-105) | 97 (61-136) | 98 (59-145) | 101 (65-147)^e^ | ***0.002*** | 89 (60-130) |  | *0.1* | *0.07* | *0.2* |
| Respiratory rate | *NA* | 23 (14-36)^a^ | 29 (16-44) | 34 (22-49) | 31 (21-55)^c^ | ***<0.001*** | 22 (15-30) |  | *0.8* | *0.07* | ***<0.001*** |
| Glasgow coma scale | *NA* | 15 (15-15) | 15 (15-15) | 15 (13-15) | 14 (3-15)^c^ | *0.1* | 15 (10-15) |  | *0.3* | *0.2* | *0.9* |
|  |  |  |  |  |  |  |  |  |  |  |  |
| Baseline laboratory values  (mean, range) |  |  |  |  |  |  |  |  |  |  |  |
| White blood cells (G/l) | *NA* | 5.8 (3-10) | 6.3 (1-15) | 9.6 (5-18) | 10.1 (3-20) | ***<0.001*** | 8.1 (4-17) |  | ***0.007*** | ***0.03*** | *0.2* |
| Hemoglobin (g/l) | *NA* | 143 (103-172) | 135 (60-176) | 130 (99-161) | 135 (83-168) | *0.3* | 137 (112-158) |  | *0.2* | *0.7* | *0.2* |
| Platelets (G/l) | *NA* | 252 (147-543) | 207 (49-430) | 223 (107-490) | 243 (29-521) | *0.3* | 195 (123-324) |  | ***0.03*** | *0.5* | *0.3* |
| Creatinine (μmol/l) | *NA* | 94 (57-185) | 103 (59-352) | 117 (41-343) | 106 (51-230) | *0.6* | 97 (54-175) |  | *0.7* | *0.6* | *0.3* |
|  |  |  |  |  |  |  |  |  |  |  |  |
| Lung infiltrates at baseline^e,f^ | *NA* | 8 (35%) | 36 (90%) | 14 (93%) | 23 (92%) | ***<0.001*** | 6 (27%) |  | *0.8* | ***<0.001*** | ***<0.001*** |
|  |  |  |  |  |  |  |  |  |  |  |  |
| Severity scores |  |  |  |  |  |  |  |  |  |  |  |
| qSOFA ≥2 | *NA* | 0 (0%) | 1 (3%) | 7 (47%) | 9 (39%)^c^ | ***<0.001*** | *0 (0%)* |  | *NA* | *1.0* | ***<0.001*** |
| CURB65 ≥2 | *NA* | 4 (18%)^b^ | 6 (15%) | 10 (67%) | 12 (57%)^e^ | ***<0.001*** | 9 (41%) |  | ***0.2*** | *0.03* | ***0.2*** |
|  |  |  |  |  |  |  |  |  |  |  |  |
| Days to sample (mean, range) |  |  |  |  |  |  |  |  |  |  |  |
| Symptoms to RNA sample | *NA* | 10.0 (2-30) | 11.4 (1-19) | 9.2 (1-21) | 21.4 (6-37) | *NA* | 7.3 (2-21) |  | *0.2* | ***0.003*** | *0.3* |
| Admission to RNA sample | *NA* | 0.8 (0-4) | 2.4 (0-7) | 1.9 (0-7) | 16.4 (8-30) | *NA* | 0.4 (0-4) |  | *0.2* | ***<0.001*** | ***0.02*** |
| Intubation to RNA sample | *NA* | *NA* | *NA* | -0.2 (-4-7) | 13.5 (3-28) | *NA* | *NA* |  | *NA* | *NA* | *NA* |
|  |  |  |  |  |  |  |  |  |  |  |  |
| Outcome |  |  |  |  |  |  |  |  |  |  |  |
| Hospitalization | *NA* | 10 (43%) | 40 (100%) | 15 (100%) | 25 (100%) | *NA* | 18 (82%) |  | ***0.01*** | ***0.01*** | *0.1* |
| ICU admission | *NA* | 0 (0%) | 11 (28%) | 15 (100%) | 25 (100%) | *NA* | 0 (0%) |  | *NA* | *NA* | *NA* |
| Mechanical ventilation | *NA* | 0 (0%) | 0 (0%) | 15 (100%) | 25 (100%) | *NA* | 0 (0%) |  | *NA* | *NA* | *NA* |
| Death (30 days) | *NA* | 0 (0%) | 1 (3%) | 7 (47%) | 3 (12%) | ***<0.001*** | 0 (0%) |  | *NA* | *1.0* | ***<0.001*** |

^a^ 19 patients were infected by Influenza virus A and 3 by Influenza virus B, ^b^ 1 missing value, ^c^ 2 missing values, ^d^ 4 missing values, ^e^ 6 missing values, ^f^ by chest X-ray, ultrasonography or computed-tomography; abbreviations: qSOFA stands for quick sequential organ failure assessment, ICU for intensive care unit, BMI for body mass index.

P value are calculated by anova for continuous variable and by a Fisher’s exact test for categorical variables.

HLTY stand for healthy controls, OXY0 for patients who did not require oxygen support, OXY1 for patients who did request oxygen support, TUBE for patients who required intubation, with samples taken <= 7 days (TUBE early) or >7 days (TUBE late) after hospital admission.
