# Supplementary material for: Transcriptomic Signature Differences Between SARS-CoV-2 and Influenza Virus Infected Patients
Source: Front Immunol. 2021 May 31;12:666163. doi: 10.3389/fimmu.2021.666163 (PMC8202013; doi:10.3389/fimmu.2021.666163)
Supplement: Supplementary Table 2 — Composition of genes sets for main figures. [file Table_2.pdf]

Supplementary Table 2

pathname	codinc	symbol	entrez
ISG	GO:0034341<br>GO:0035455<br>GO:0035456	ACOD1 ACTG1 ACTR2 ACTR3 ADAMTS13 AIF1 AQP4 ARG1 ASS1 B2M BST2 CALCOCO2 CAMK2A CAMK2B CAMK2D CAMK2G CASP1 CCL1 CCL11 CCL13 CCL14 CCL15 CCL16 CCL17 CCL18 CCL19 CCL2 CCL20 CCL21 CCL22 CCL23 CCL24 CCL25 CCL26 CCL3 CCL3L1 CCL3L3 CCL4 CCL5 CCL7 CCL8 CD40 CD44 CD47 CD58 CDC37 CDC42 CDC42EP2 CDC42EP4 CIITA CITED1 CLDN1 CX3CL1 CXCL16 CYP27B1 DAPK1 DAPK3 EDN1 EPRS1 EVL FASLG FCGR1A FCGR1B FLNB GAPDH GBP1 GBP2 GBP3 GBP4 GBP5 GBP6 GBP7 GCH1 GSN HCK HLA-A HLA-B HLA-C HLA-DPA1 HLA-DPB1 HLA-DQA1 HLA-DQA2 HLA-DQB1 HLA-DQB2 HLA-DRA HLA-DRB1 HLA-DRB3 HLA-DRB4 HLA-DRB5 HLA-E HLA-F HLA-G HLA-H HPX HSP90AB1 ICAM1 IFI30 IFITM1 IFITM2 IFITM3 IFNG IFNGR1 IFNGR2 IL12B IL12RB1 IL23R IRF1 IRF2 IRF3 IRF4 IRF5 IRF6 IRF7 IRF8 IRF9 IRGM JAK1 JAK2 KIF16B KIF5B KYNU LGALS9 MED1 MEFV MID1 MRC1 MT2A MYO1C NCAM1 NLRC5 NMI NOS2 NR1H2 NR1H3 NUB1 OAS1 OAS2 OAS3 OASL OTOP1 PARP14 PARP9 PDE12 PIAS1 PML PPARG PRKCD PTAFR PTPN2 RAB12 RAB20 RAB43 RAB7B RPL13A SEC61A1 SHFL SIRPA SLC11A1 SLC26A6 SLC30A8 SNCA SOCS1 SOCS3 SP100 STAR STAT1 STX4 STX8 STXBP1 STXBP2 STXBP3 STXBP4 SUMO1 SYNCRIP TDGF1 TLR2 TLR3 TLR4 TP53 TRIM21 TRIM22 TRIM25 TRIM26 TRIM31 TRIM34 TRIM38 TRIM5 TRIM62 TRIM68 TRIM8 TXK UBD VAMP3 VCAM1 VIM VPS26B WAS WNT5A XCL1 XCL2 ZYX AIM2 CAPN2 CDC34 DDX41 HTRA2 IFI16 IFNAR2 IFNB1 IKBKE IRGC MNDA NDUFA13 PLSCR1 PNPT1 PYHIN1 STING1 TREX1 TRIM6 UBE2G2 UBE2K XAF1 ADAR AXL EIF2AK2 GAS6 GATA3 IFIT2 IFIT3 IFNAR1 KLHL20 LAMP3 MX2 RO60 TPR	10062 10096 10097 10241 10344 10346 10379 10410 10435 10437 10475 10492 10537 10581 11074 11093 11140 112936 115361 115362 133060 140885 149233 1594 1612 1613 163351 169026 1906 199 201475 201626 2058 2209 2210 2317 23521 23580 252983 2597 2633 2634 2635 2643 2934 29927 3055 3105 3106 3107 3113 3115 3117 3118 3119 3120 3122 3123 3125 3126 3127 3133 3134 3135 3136 3263 3326 3383 338382 339122 3394 345611 3458 3459 3460 356 3593 3594 361 3659 3660 3661 3662 3663 3664 3665 3716 3717 3799 383 388646 3965 414062 4210 4261 4281 4360 4435 445 4502 4641 4684 4843 4938 4939 4940 51466 51667 5371 53840 54625 5468 5469 55128 55223 55337 55614 55647 5580 567 5724 5771 58191 6346 6347 6348 6349 6351 6352 6354 6355 6356 6357 6358 6359 6360 6361 6362 6363 6364 6366 6367 6368 6369 6370 6375 6376 65010 6556 6622 6672 6737 6770 6772 6810 6812 6813 6814 684 6846 6997 7097 7098 7099 71 7157 7294 730249 7341 7376 7412 7431 7454 7474 7706 7726 7791 815 816 81603 817 818 834 83666 84166 8519 85363 8554 8638 8651 8942 9021 9076 9111 9341 9482 958 960 961 965 998 103 149628 2621 2625 27074 27252 3433 3437 3454 3455 4600 558 5610 6738 7175 11277 117854 27429 3093 340061 3428 3456 4332 51079 51428 5359 54739 56269 7327 824 87178 9447 9641 997
SIG_PRR	GO:0034162<br>KEGG_hsa04620<br>KEGG_hsa04621<br>KEGG_hsa04622	AIM2 ANTXR1 ANTXR2 ATG12 ATG16L1 ATG5 BCL2 BCL2L1 BIRC2 BIRC3 BRCC3 CAMP CARD16 CARD17 CARD18 CARD6 CARD8 CARD9 CASP1 CASP12 CASP4 CASP5 CASP8 CASR CCL2 CCL5 CHUK CTSB CXCL1 CXCL2 CXCL3 CXCL8 CYBA CYBB DEFA1 DEFA1B DEFA3 DEFA4 DEFA5 DEFA6 DEFB103A DEFB103B DEFB4A DEFB4B DHX33 DNM1L ERBIN FADD GABARAP GABARAPL1 GABARAPL2 GBP1 GBP2 GBP3 GBP4 GBP5 GBP7 GPRC6A GSDMD HSP90AA1 HSP90AB1 IFI16 IFNA1 IFNA10 IFNA13 IFNA14 IFNA16 IFNA17 IFNA2 IFNA21 IFNA4 IFNA5 IFNA6 IFNA7 IFNA8 IFNAR1 IFNAR2 IFNB1 IKBKB IKBKE IKBKG IL18 IL1B IL6 IRAK4 IRF3 IRF7 IRF9 ITPR1 ITPR2 ITPR3 JAK1 JUN MAP3K7 MAPK1 MAPK10 MAPK11 MAPK12 MAPK13 MAPK14 MAPK3 MAPK8 MAPK9 MAVS MCU MEFV MFN1 MFN2 MYD88 NAIP NAMPT NEK7 NFKB1 NFKBIA NFKBIB NLRC4 NLRP1 NLRP12 NLRP3 NLRP6 NLRP7 NLRX1 NOD1 NOD2 OAS1 OAS2 OAS3 P2RX7 PANX1 PKN1 PKN2 PLCB1 PLCB2 PLCB3 PLCB4 PRKCD PSTPIP1 PYCARD PYDC1 PYDC2 PYDC5 RBCK1 RELA RHOA RIPK1 RIPK2 RIPK3 RNASEL RNF31 SHARPIN STAT1 STAT2 STING1 SUGT1 TAB1 TAB2 TAB3 TANK TBK1 TICAM1 TLR4 TNF TNFAIP3 TP53BP1 TRAF2 TRAF3 TRAF5 TRAF6 TRIP6 TRPM2 TRPM7 TRPV2 TXN TXN2 TXNIP TYK2 VDAC1 VDAC2 VDAC3 XIAP YWHAE CCL11 CCL13 CCL7 CCL8 HSP90B1 AKT1 AKT2 AKT3 CCL3 CCL3L1 CCL3L3 CCL4 CCL4L1 CCL4L2 CD14 CD40 CD80 CD86 CTSK CXCL10 CXCL11 CXCL9 FOS IL12A IL12B IRAK1 IRF5 LBP LY96 MAP2K1 MAP2K2 MAP2K3 MAP2K4 MAP2K6 MAP2K7 MAP3K8 PIK3CA PIK3CB PIK3CD PIK3R1 PIK3R2 PIK3R3 RAC1 SPP1 TICAM2 TIRAP TLR1 TLR2 TLR3 TLR5 TLR6 TLR7 TLR8 TLR9 TOLLIP PIK3CG PIK3R5 AZI2 CASP10 CYLD DDX3X DDX3Y DDX58 DHX58 IFIH1 IFNE IFNK IFNW1 ISG15 MAP3K1 OTUD5 PIN1 RNF125 SIKE1 TBKBP1 TKFC TRADD TRIM25 EPG5 FCRL3 GRAMD4 HAVCR2 HMGB1 IRAK2 LILRA4 NR1H4 PIK3AP1 PIK3C3 PIK3R4 PTPN22 PTPRS RAB7B RSAD2 RTN4 TNIP2 UNC93B1	10000 10333 10454 114609 1147 1326 1432 148022 1513 207 208 23118 2353 23533 23643 29110 3439 3440 3441 3442 3443 3444 3445 3446 3447 3448 3449 3451 3452 3454 3455 3456 353376 3551 3553 3569 3576 3592 3593 3627 3654 3661 3663 3665 3725 3929 4283 4615 4790 4792 51135 51284 51311 5290 5291 5293 5294 5295 5296 54106 54472 5594 5595 5599 5600 5601 5602 5603 5604 5605 5606 5608 5609 5879 5970 6300 6348 6351 6352 6373 6416 6696 6772 6885 7096 7097 7098 7099 7100 7124 7187 7189 841 8503 8517 8737 8772 929 941 942 958 9641 388372 414062 6349 9560 10010 100289462 100506742 10059 10135 10379 10392 10616 10628 107181291 10910 11035 11337 11345 114548 114769 115361 115362 118429 140609 1508 152138 1535 1536 1667 1668 1669 1670 1671 1673 171389 199713 222545 22861 22900 23236 23710 24145 257397 25828 260434 2633 2634 2635 29108 2919 2920 2921 329 330 331 3320 3326 340061 3428 3606 3708 3709 3710 3716 387 388646 414325 4210 440068 4671 4793 4938 4939 4940 5027 51393 5330 5331 5332 54822 55054 55072 55669 5580 5585 5586 55894 55914 56919 57506 58484 59082 596 598 6041 6347 64127 64170 6773 7128 7158 7186 7188 7205 7226 728358 7295 7297 7416 7417 7419 7531 79184 79671 79792 81858 820 834 837 838 84168 846 84674 8767 9051 90550 9140 91662 9447 9474 9927 6354 6355 6356 6357 7184 1540 1654 23586 26007 338376 3467 4214 5300 54941 55593 56832 64135 64343 7706 79132 80143 843 8653 8717 9636 9755 115352 118788 23151 23547 26191 30849 3146 338382 3656 5289 57142 57724 5802 79155 81622 84868 91543 9971
MHCI	GO:0042612<br>REACT_75795	B2M BCAP31 CALR CANX ERAP1 ERAP2 HLA-A HLA-B HLA-C HLA-E HLA-F HLA-G HSPA5 PDIA3 SAR1B SEC13 SEC23A SEC24A SEC24B SEC24C SEC24D SEC31A TAP1 TAP2 TAPBP HLA-H MR1	10134 10427 10484 10802 22872 2923 3105 3106 3107 3133 3134 3135 3309 51128 51752 567 6396 64167 6890 6891 6892 811 821 9632 9871 3136 3140

PROTEASOME	KEGG_hsa03050	ADRM1 IFNG POMP PSMA1 PSMA2 PSMA3 PSMA4 PSMA5 PSMA6 PSMA7 PSMA8 PSMB1 PSMB10 PSMB11 PSMB2 PSMB3 PSMB4 PSMB5 PSMB6 PSMB7 PSMB8 PSMB9 PSMC1 PSMC2 PSMC3 PSMC4 PSMC5 PSMC6 PSMD1 PSMD11 PSMD12 PSMD13 PSMD14 PSMD2 PSMD3 PSMD4 PSMD6 PSMD7 PSMD8 PSMD9 PSME1 PSME2 PSME3 PSME4 PSMF1 SEM1 PSMA6P4 PSMC1P4	10197 10213 11047 122706 143471 23198 3458 51371 5682 5683 5684 5685 5686 5687 5688 5689 5690 5691 5692 5693 5694 5695 5696 5698 5699 5700 5701 5702 5704 5705 5706 5707 5708 5709 5710 5713 5714 5715 5717 5718 5719 5720 5721 7979 9491 9861 121906 345645
MHCII_GENES	GO:0042613	CD74 HLA-DMA HLA-DMB HLA-DOA HLA-DOB HLA-DPA1 HLA-DPB1 HLA-DQA1 HLA-DQA2 HLA-DQB1 HLA-DQB2 HLA-DRA HLA-DRB1 HLA-DRB3 HLA-DRB4 HLA-DRB5	3108 3109 3111 3112 3113 3115 3117 3118 3119 3120 3122 3123 3125 3126 3127 972
ENDOSOME	GO:0031904	AP4B1 AP4E1 AP4M1 AP4S1 APOB APP B2M CD63 CSH1 CTSB CTSH CTSK CTSL CTSS GH1 GH2 IL12A IL12B INS JAK2 LGMN LNPEP LRP2 LRPAP1 NAPSA NGF PDLIM4 PGA3 PGA4 PGA5 PRF1 PRL PRLR SFTPB SFTPC	10717 11154 1442 1508 1512 1513 1514 1520 23431 2688 2689 338 351 3592 3593 3630 3717 4012 4036 4043 4803 5222 5551 5617 5618 5641 567 643834 643847 6439 6440 8572 9179 9476 967
CYTOK_CHEMOK_R	KEGG_hsa04060	ACKR3 ACKR4 ACVR1 ACVR1B ACVR1C ACVR2A ACVR2B ACVRL1 AMH AMHR2 BMP10 BMP15 BMP2 BMP3 BMP4 BMP5 BMP6 BMP7 BMP8A BMP8B BMPR1A BMPR1B BMPR2 CCL1 CCL11 CCL13 CCL14 CCL15 CCL16 CCL17 CCL18 CCL19 CCL2 CCL20 CCL21 CCL22 CCL23 CCL24 CCL25 CCL26 CCL27 CCL28 CCL3 CCL3L1 CCL3L3 CCL4 CCL4L1 CCL4L2 CCL5 CCL7 CCL8 CCR1 CCR10 CCR2 CCR3 CCR4 CCR5 CCR6 CCR7 CCR8 CCR9 CD27 CD4 CD40 CD40LG CD70 CLCF1 CNTF CNTFR CRLF2 CSF1 CSF1R CSF2 CSF2RA CSF2RB CSF3 CSF3R CSH1 CSH2 CTF1 CX3CL1 CX3CR1 CXCL1 CXCL10 CXCL11 CXCL12 CXCL13 CXCL14 CXCL16 CXCL17 CXCL2 CXCL3 CXCL5 CXCL6 CXCL8 CXCL9 CXCR1 CXCR2 CXCR3 CXCR4 CXCR5 CXCR6 EDA EDA2R EDAR EPO EPOR FAS FASLG GDF1 GDF10 GDF11 GDF15 GDF2 GDF3 GDF5 GDF6 GDF7 GDF9 GH1 GH2 GHR IFNA1 IFNA10 IFNA13 IFNA14 IFNA16 IFNA17 IFNA2 IFNA21 IFNA4 IFNA5 IFNA6 IFNA7 IFNA8 IFNAR1 IFNAR2 IFNB1 IFNE IFNG IFNGR1 IFNGR2 IFNK IFNL1 IFNL2 IFNL3 IFNLR1 IFNW1 IL10 IL10RA IL10RB IL11 IL11RA IL12A IL12B IL12RB1 IL12RB2 IL13 IL13RA1 IL13RA2 IL15 IL15RA IL16 IL17A IL17B IL17C IL17D IL17F IL17RA IL17RB IL17RC IL17RE IL18 IL18R1 IL18RAP IL19 IL1A IL1B IL1F10 IL1R1 IL1R2 IL1RAP IL1RL1 IL1RL2 IL1RN IL2 IL20 IL20RA IL20RB IL21 IL21R IL22 IL22RA1 IL23A IL23R IL24 IL25 IL26 IL27 IL27RA IL2RA IL2RB IL2RG IL3 IL31 IL31RA IL32 IL33 IL34 IL36A IL36B IL36G IL36RN IL37 IL3RA IL4 IL4R IL5 IL5RA IL6 IL6R IL6ST IL7 IL7R IL9 IL9R INHA INHBA INHBB INHBC INHBE LEP LEPR LIF LIFR LTA LTB LTBR MPL MSTN NGF NGFR NODAL OSM OSMR PF4 PF4V1 PPBP PRL PRLR RELT TGFB1 TGFB2 TGFB3 TGFBR1 TGFBR2 THPO TNF TNFRSF10A TNFRSF10B TNFRSF10C TNFRSF10D TNFRSF11A TNFRSF11B TNFRSF12A TNFRSF13B TNFRSF13C TNFRSF14 TNFRSF17 TNFRSF18 TNFRSF19 TNFRSF1A TNFRSF1B TNFRSF21 TNFRSF25 TNFRSF4 TNFRSF6B TNFRSF8 TNFRSF9 TNFSF10 TNFSF11 TNFSF12 TNFSF13 TNFSF13B TNFSF14 TNFSF15 TNFSF18 TNFSF4 TNFSF8 TNFSF9 TSLP XCL1 XCL2 XCR1 EGF EGFR FLT1 FLT3 FLT3LG FLT4 HGF IL22RA2 KDR KIT KITLG MET PDGFA PDGFB PDGFC PDGFRA PDGFRB PLEKHO2 PPBPP1 TPO VEGFA VEGFB VEGFC VEGFD	10220 10344 10563 10663 10673 10803 10850 10913 11009 112744 115650 1230 1232 1233 1234 1235 1236 1237 1270 1271 130399 132014 133396 1435 1436 1437 1438 1439 1440 1441 1442 1443 146433 1489 149233 151449 1524 163702 1896 2056 2057 23495 23529 23765 246778 26525 2657 2658 2660 2661 2662 268 2688 2689 269 2690 27177 27178 27179 27189 27190 27242 27302 2826 282616 282617 282618 2829 2833 284340 2919 2920 2921 29949 338376 3439 3440 3441 3442 3443 3444 3445 3446 3447 3448 3449 3451 3452 3454 3455 3456 3458 3459 3460 3467 353500 355 3552 3553 3554 3556 3557 3558 3559 356 3560 3561 3562 3563 3565 3566 3567 3568 3569 3570 3572 3574 3575 3576 3577 3578 3579 3581 3586 3587 3588 3589 3590 3592 3593 3594 3595 3596 3597 3598 3600 3601 3603 3604 3605 3606 3623 3624 3625 3626 3627 386653 388372 392255 3952 3953 3976 3977 4049 4050 4055 414062 4283 4352 4803 4804 4838 4982 5008 50604 50615 50616 51330 51554 51561 5196 5197 53342 53832 53833 5473 55504 55540 55801 5617 5618 56300 56477 56832 57007 58191 58985 59067 60401 608 6346 6347 6348 6349 6351 6352 6354 6355 6356 6357 6358 6359 6360 6361 6362 6363 6364 6366 6367 6368 6369 6370 6372 6373 6374 6375 6376 6387 64109 643 64806 650 651 652 653 654 655 656 657 658 659 6846 7040 7042 7043 7046 7048 7066 7124 7132 7133 7292 729230 7293 7850 7852 8200 83729 84639 84818 84957 85480 8600 8718 8740 8741 8742 8743 8744 8764 8771 8784 8792 8793 8794 8795 8797 8807 8808 8809 8995 90 90865 91 9173 9180 92 920 9210 9235 93 939 94 943 944 9466 9518 9547 9560 9573 958 959 970 9966 116379 1950 1956 2277 2321 2322 2323 2324 3082 3791 3815 4233 4254 5154 5155 5156 5159 56034 7173 728045 7422 7423 7424 80301

BCELL	GO:0019724<br>GO:0030890<br>GO:0045579<br>GO:0048304<br>GO:0050861<br>KEGG_hsa04662	ADA ATAD5 BCL2 BCL6 BST1 CARD11 CD320 CD38 CD40 CD74 CD81 CDKN1A CHRNB2 CLCF1 FCRL3 GPR183 IL13 IL2 IL21 IL4 IL5 IL7 IRS2 MEF2C MIF NCKAP1L NFATC2 PELI1 PTPRC SASH3 SLC39A10 TFRC TICAM1 TIRAP TLR4 TLR9 TNFRSF13C TNFRSF4 TNFSF13B VAV3 WNT3A AICDA APCS APLF BATF BCL10 BCL3 BTK C17orf99 C1QA C1QB C1QBP C1QC C1R C1RL C1S C2 C3 C4A C4B C4BPA C4BPB C5 C6 C7 C8A C8B C8G C9 CCR6 CD19 CD226 CD27 CD28 CD40LG CD46 CD55 CD70 CFI CLU CR1 CR2 CRP ERCC1 EXO1 EXOSC3 EXOSC6 FCER1G FCER2 FCGR2B FGL2 FOXJ1 FOXP3 GAPT GCNT3 HLA-DQB1 HLA-DRB1 HLA-E HLA-G HMCES HPX HSPD1 IGHA1 IGHA2 IGHD IGHE IGHG1 IGHG2 IGHG3 IGHG4 IGHM IGHV1-18 IGHV1-24 IGHV1-3 IGHV1-45 IGHV1-58 IGHV1-69 IGHV1-69-2 IGHV1-69D IGHV1OR15-1 IGHV2-26 IGHV2-5 IGHV2-70 IGHV2-70D IGHV3-11 IGHV3-13 IGHV3-15 IGHV3-16 IGHV3-20 IGHV3-21 IGHV3-23 IGHV3-30 IGHV3-33 IGHV3-35 IGHV3-38 IGHV3-43 IGHV3-48 IGHV3-49 IGHV3-53 IGHV3-64 IGHV3-64D IGHV3-66 IGHV3-7 IGHV3-72 IGHV3-73 IGHV3-74 IGHV4-28 IGHV4-31 IGHV4-34 IGHV4-39 IGHV4-4 IGHV4-59 IGHV4-61 IGHV5-10-1 IGHV5-51 IGHV6-1 IGHV7-4-1 IGHV7-81 IGKC IGKV1-12 IGKV1-16 IGKV1-17 IGKV1-39 IGKV1-5 IGKV1D-12 IGKV1D-33 IGKV1D-39 IGKV2-28 IGKV2-29 IGKV2-30 IGKV2-40 IGKV2D-28 IGKV2D-30 IGKV3-15 IGKV3-20 IGKV3D-11 IGKV3D-20 IGKV4-1 IGKV5-2 IGLC1 IGLC2 IGLC3 IGLC6 IGLC7 IGLL1 IGLL5 IGLV1-40 IGLV1-44 IGLV1-47 IGLV1-51 IGLV2-11 IGLV2-14 IGLV2-23 IGLV2-8 IGLV3-1 IGLV3-19 IGLV3-21 IGLV3-25 IGLV3-27 IGLV6-57 IGLV7-43 IL10 IL13RA2 IL27RA IL4R INPP5D IRF7 KMT5B KMT5C LIG4 LTA MAD2L2 MASP2 MBL2 MLH1 MSH2 MSH6 NBN NDFIP1 NECTIN2 NSD2 PARP3 PAXIP1 POU2F2 PRKCD PTPN6 RIF1 RNF168 RNF8 SERPING1 SHLD1 SHLD2 SHLD3 SLA2 STAT6 SUPT6H SUSD4 SWAP70 TBX21 TCIRG1 TGFB1 THOC1 TLR8 TNF TNFSF13 TNFSF4 TP53BP1 TRBC1 TRBC2 TRDC TREX1 UNG XCL1 ZP3 AKT1 AKT2 AKT3 BLNK CD22 CD72 CD79A CD79B CHP1 CHP2 CHUK DAPP1 FOS GRB2 GSK3B HRAS IFITM1 IKBKB IKBKG JUN KRAS LILRB3 LYN MALT1 MAP2K1 MAP2K2 MAPK1 MAPK3 NFAT5 NFATC1 NFATC3 NFATC4 NFKB1 NFKBIA NFKBIB NFKBIE NRAS PIK3AP1 PIK3CA PIK3CB PIK3CD PIK3CG PIK3R1 PIK3R2 PIK3R3 PIK3R5 PLCG2 PPP3CA PPP3CB PPP3CC PPP3R1 PPP3R2 PRKCB RAC1 RAC2 RAC3 RAF1 RASGRP3 RELA SOS1 SOS2 SYK VAV1 VAV2 IGH INPPL1 LILRA1 LILRA2 LILRA3 LILRA4 LILRA5 LILRA6 LILRB1 LILRB2 LILRB4 LILRB5 leukocyte immunoglobulin-like receptor subfamily B member 3 [KO:K06512] ATP11C BAD MMP14 PCID2 PPP2R3C STAT5B XBP1 CMTM3 FOXP1 MIR18A MIR19A	100 1026 10451 10673 1141 114609 115352 115650 148022 1880 23529 3071 3558 3565 3567 3574 3596 4208 4282 4773 51293 54106 54440 57162 57181 5788 59067 596 604 683 7037 7099 7293 79915 84433 8660 89780 952 958 972 975 10000 102723407 102725035 10288 10859 10892 10990 11006 11024 11025 11026 11027 1147 118788 1380 207 208 2213 2353 23547 25780 27071 2885 2932 29760 3265 353514 3551 3635 3636 3725 3845 4067 4772 4775 4790 4792 4793 4794 4893 5290 5291 5293 5295 5296 5336 5530 5532 5533 5534 5535 5579 5594 5595 5604 5605 5777 5879 5880 5881 5894 5970 6654 6655 6850 695 7409 7410 79168 8503 8517 8519 8915 930 933 971 973 974 10725 11261 23533 4776 5294 63928 100141515 10039 100423062 102723168 102723169 102723170 10312 10459 10538 10666 10747 10875 112441434 11277 118460 1191 1235 1378 1401 149840 1604 165918 200558 202309 2067 2207 2208 22976 2302 23075 28299 28378 28385 28386 28388 28391 28392 28394 28395 28396 28400 28401 28408 28409 28410 28412 28414 28420 28423 28424 28426 28429 28432 28434 28439 28442 28444 28445 28447 28448 28449 28450 28452 28454 28455 28457 28458 28461 28464 28466 28467 28468 28473 28526 28638 28639 28776 28778 28791 28793 28796 28797 28809 28813 28815 28816 28817 28820 28822 28823 28825 28834 28874 28876 28881 28883 28893 28896 28903 28907 28908 28912 28913 28916 28919 28920 28921 28930 28937 28938 28940 2956 30009 3119 3123 3133 3135 325 3263 3329 3426 3493 3494 3495 3497 3500 3501 3502 3503 3507 3514 3537 3538 3539 3542 3543 3566 3586 3598 3665 388077 3981 4049 4153 4179 4292 4436 4683 50943 51010 51111 51279 51311 5452 54537 55061 55183 5580 56941 57289 57379 5819 602 6375 6778 6830 7040 708 710 712 7124 713 714 715 7158 716 717 718 720 721 722 725 727 729 7292 730 731 732 733 735 7374 7468 7784 80762 84174 84787 8741 9025 9156 9245 939 940 9466 959 970 9984 123920 150372 26228 27086 406953 406979 5583 286410 4323 55012 55795 572 6777 7494
IG_LIST	GO:0019814	CD79A CD79B IGHA1 IGHA2 IGHD IGHD1-1 IGHE IGHG1 IGHG2 IGHG3 IGHG4 IGHJ1 IGHM IGHV1-18 IGHV1-24 IGHV1-3 IGHV1-45 IGHV1-58 IGHV1-69 IGHV1-69-2 IGHV1-69D IGHV1OR15-1 IGHV2-26 IGHV2-5 IGHV2-70 IGHV2-70D IGHV3-11 IGHV3-13 IGHV3-15 IGHV3-16 IGHV3-20 IGHV3-21 IGHV3-23 IGHV3-30 IGHV3-33 IGHV3-35 IGHV3-38 IGHV3-43 IGHV3-48 IGHV3-49 IGHV3-53 IGHV3-64 IGHV3-64D IGHV3-66 IGHV3-7 IGHV3-72 IGHV3-73 IGHV3-74 IGHV4-28 IGHV4-31 IGHV4-34 IGHV4-39 IGHV4-4 IGHV4-59 IGHV4-61 IGHV5-10-1 IGHV5-51 IGHV6-1 IGHV7-4-1 IGHV7-81 IGKC IGKJ1 IGKV1-12 IGKV1-13 IGKV1-16 IGKV1-17 IGKV1-27 IGKV1-37 IGKV1-39 IGKV1-5 IGKV1-6 IGKV1-8 IGKV1-9 IGKV1D-12 IGKV1D-13 IGKV1D-17 IGKV1D-33 IGKV1D-37 IGKV1D-39 IGKV1D-42 IGKV1D-43 IGKV1D-8 IGKV2-24 IGKV2-28 IGKV2-29 IGKV2-30 IGKV2-40 IGKV2D-24 IGKV2D-26 IGKV2D-28 IGKV2D-29 IGKV2D-30 IGKV3-15 IGKV3-20 IGKV3-7 IGKV3D-11 IGKV3D-15 IGKV3D-20 IGKV3D-7 IGKV4-1 IGKV5-2 IGKV6-21 IGKV6D-21 IGKV6D-41 IGLC1 IGLC2 IGLC3 IGLC6 IGLC7 IGLJ1 IGLL1 IGLL5 IGLV1-36 IGLV1-40 IGLV1-44 IGLV1-47 IGLV1-50 IGLV1-51 IGLV10-54 IGLV11-55 IGLV2-11 IGLV2-14 IGLV2-18 IGLV2-23 IGLV2-33 IGLV2-8 IGLV3-1 IGLV3-10 IGLV3-12 IGLV3-16 IGLV3-19 IGLV3-21 IGLV3-22 IGLV3-25 IGLV3-27 IGLV3-32 IGLV3-9 IGLV4-3 IGLV4-60 IGLV4-69 IGLV5-37 IGLV5-45 IGLV5-48 IGLV5-52 IGLV6-57 IGLV7-43 IGLV7-46 IGLV8-61 IGLV9-49 JCHAIN LIME1 SYK TRBC1 TRBC2 TRDC	100423062 102723168 102723169 102723170 28299 28378 28385 28386 28388 28391 28392 28394 28395 28396 28400 28401 28408 28409 28410 28412 28414 28420 28423 28424 28426 28429 28432 28434 28439 28442 28444 28445 28447 28448 28449 28450 28452 28454 28455 28457 28458 28461 28464 28466 28467 28468 28473 28483 28510 28526 28638 28639 28770 28772 28773 28774 28775 28776 28778 28779 28780 28781 28783 28784 28785 28786 28787 28791 28793 28795 28796 28797 28799 28802 28803 28804 28809 28811 28813 28814 28815 28816 28817 28820 28821 28822 28823 28825 28826 28833 28834 28869 28870 28874 28875 28876 28877 28881 28882 28883 28884 28885 28891 28892 28893 28894 28896 28900 28902 28903 28904 28906 28907 28908 28912 28913 28915 28916 28919 28920 28921 28923 28930 28931 28935 28937 28938 28939 28940 28941 28942 28943 28950 3493 3494 3495 3497 3500 3501 3502 3503 3507 3512 3514 3537 3538 3539 3542 3543 388077 54923 57289 6850 973 974
TH_17	GO:2000318<br>GO:2000321	IL12B IL12RB1 IL23A IL23R MALT1 MIR21 NFKBID NFKBIZ NLRP10 NLRP3 PRKCQ	10892 114548 149233 338322 3593 3594 406991 51561 5588 64332 84807

Supplementary Table 2

NKCELL	GO:0001865 GO:0001866 GO:0002370 GO:0043320 KEGG_hsa04650 MAN_NK	AP1G1 CORO1A HLA-F KLRF2 LAMP1 RAB27A UNC13D VAMP7 AP3B1 AP3D1 ITK PRDM1 PSAP TGFBR2 TOX ZBTB16 ZBTB7B ZNF683 ARAF BID BRAF CASP3 CD244 CD247 CD48 CHP1 CHP2 CSF2 FAS FASLG FCER1G FCGR3A FCGR3B FYN GRB2 GZMB HCST HLA-A HLA-B HLA-C HLA-E HLA-G HRAS ICAM1 ICAM2 IFNA1 IFNA10 IFNA13 IFNA14 IFNA16 IFNA17 IFNA2 IFNA21 IFNA4 IFNA5 IFNA6 IFNA7 IFNA8 IFNAR1 IFNAR2 IFNB1 IFNG IFNGR1 IFNGR2 ITGAL ITGB2 KIR2DL1 KIR2DL2 KIR2DL3 KIR2DL4 KIR2DL5A KIR2DS1 KIR2DS3 KIR2DS4 KIR2DS5 KIR3DL1 KIR3DL2 KLRC1 KLRC2 KLRC3 KLRD1 KLRK1 KRAS LAT LCK LCP2 MAP2K1 MAP2K2 MAPK1 MAPK3 MICA MICB NCR1 NCR2 NCR3 NFAT5 NFATC1 NFATC2 NFATC3 NFATC4 NRAS PAK1 PIK3CA PIK3CB PIK3CD PIK3CG PIK3R1 PIK3R2 PIK3R3 PIK3R5 PLCG1 PLCG2 PPP3CA PPP3CB PPP3CC PPP3R1 PPP3R2 PRF1 PRKCA PRKCB PRKCG PTK2B PTPN11 PTPN6 RAC1 RAC2 RAC3 RAET1E RAET1G RAET1L RAF1 SH2D1A SH2D1B SH3BP2 SHC1 SHC2 SHC3 SHC4 SOS1 SOS2 SYK TNF TNFRSF10A TNFRSF10B TNFRSF10C TNFRSF10D TNFSF10 TYROBP ULBP1 ULBP2 ULBP3 VAV1 VAV2 VAV3 ZAP70 IGH KIR2DS2 KIR3DL3 KLRC4-KLRK1 CD160 CD226 CD96 CLNK ELF4 IL12B IL15 IL18 IL23A RASAL3 GNLY TBX21 FGFBP2	100132285 100507436 100528032 102723407 10451 10870 115653 117157 135250 1437 154064 2185 2207 2214 2215 22914 2534 25759 259197 27040 2885 3002 3105 3106 3107 3133 3135 3265 3383 3384 3439 3440 3441 3442 3443 3444 3445 3446 3447 3448 3449 3451 3452 3454 3455 3456 3458 3459 3460 353091 355 356 3683 3689 369 3802 3803 3804 3806 3808 3809 3810 3811 3812 3821 3822 3823 3824 3845 3932 3937 399694 4068 4277 4772 4773 4893 5058 51744 5290 5291 5293 5295 5296 5335 53358 5336 5530 5532 5533 5534 5535 5551 5578 5579 5582 5594 5595 5604 5605 57292 5777 5781 5879 5880 5881 5894 637 6452 6464 6654 6655 673 6850 7124 7305 7409 7410 7535 79465 80328 80329 836 8503 8743 8795 8797 919 9436 9437 962 10725 11261 23533 3805 4775 4776 5294 63928 8793 8794 100431172 11151 164 201294 3134 3916 5873 6845 10225 10666 11126 116449 10578 30009 83888 3902 2000 3593 3600 3606 51043 51561 64926 257101 3702 5660 639 7048 7704 8546 8943 9760
MPHAGE	GO:0043032 GO:0045651	CASP8 CSF1 FADD HCLS1 ID2 IL34 LIF MIR145 PF4 PRKCA RB1 RIPK1 ROR2 TGFB1 TRIB1 CCL3 CEBPA CTSC HAVCR2 HSPD1 IL10 IL13 IL1RL1 IL33 IL4R KARS1 LBP LRRK2 MIR128-1 MIR128-2 MIR142 MMP8 PLA2G4A SPACA3 STAP1 TAFA3 THBS1 TLR4 TLR6 TNIP2 TREM2 TTBK1 WNT5A	10221 1435 146433 3059 3398 3976 406937 4920 5196 5578 5925 7040 841 8737 8772 10333 1050 1075 120892 124912 26228 284467 3329 3566 3586 3596 3735 3929 406915 406916 406934 4317 5321 54209 6348 7057 7099 7474 79155 84630 84868 90865 9173
CELL_SENESC	KEGG_hsa04218	AKT1 AKT2 AKT3 ATM ATR BTRC CACNA1D CALM1 CALM2 CALM3 CALML3 CALML4 CALML5 CALML6 CAPN1 CAPN2 CCNA1 CCNA2 CCNB1 CCNB2 CCNB3 CCND1 CCND2 CCND3 CCNE1 CCNE2 CDC25A CDK1 CDK2 CDK4 CDK6 CDKN1A CDKN2A CDKN2B CHEK1 CHEK2 CXCL8 E2F1 E2F2 E2F3 E2F4 E2F5 EIF4EBP1 ETS1 FBXW11 FOXM1 FOXO1 FOXO3 GADD45A GADD45B GADD45G GATA4 HIPK1 HIPK2 HIPK3 HIPK4 HLA-A HLA-B HLA-C HLA-E HLA-F HLA-G HRAS HUS1 IGFBP3 IL1A IL6 ITPR1 ITPR2 ITPR3 KIR2DL4 KRAS LIN37 LIN52 LIN54 LIN9 MAP2K1 MAP2K2 MAP2K3 MAP2K6 MAPK1 MAPK11 MAPK12 MAPK13 MAPK14 MAPK3 MAPKAPK2 MCU MDM2 MRAS MRE11 MTOR MYBL2 MYC NBN NFATC1 NFATC2 NFATC3 NFATC4 NFKB1 NRAS PIK3CA PIK3CB PIK3CD PIK3R1 PIK3R2 PIK3R3 PPID PPP1CA PPP1CB PPP1CC PPP3CA PPP3CB PPP3CC PPP3R1 PPP3R2 PTEN RAD1 RAD50 RAD9A RAD9B RAF1 RASSF5 RB1 RBBP4 RBL1 RBL2 RELA RHEB RRAS RRAS2 SERPINE1 SIRT1 SLC25A31 SLC25A4 SLC25A5 SLC25A6 SMAD2 SMAD3 SQSTM1 TGFB1 TGFB2 TGFB3 TGFBR1 TGFBR2 TP53 TRAF3IP2 TRPM7 TRPV4 TSC1 TSC2 VDAC1 VDAC2 VDAC3 ZFP36L1 ZFP36L2	10000 10111 10114 1017 1019 1021 1026 1029 1030 10758 10912 1111 11200 132660 1432 144715 147746 163688 1647 1869 1870 1871 1874 1875 1978 204851 207 208 2113 22800 22808 2305 2308 2309 23291 23411 2475 2626 286826 28996 291 292 293 3105 3106 3107 3133 3134 3135 3265 3364 3486 3552 3569 3576 3708 3709 3710 3805 3845 4087 4088 4193 4361 4605 4609 4616 4683 472 4772 4773 4775 4776 4790 4893 5054 51806 5290 5291 5293 5295 5296 545 5481 54822 5499 5500 5501 5530 5532 5533 5534 5535 5594 5595 55957 5600 5603 5604 5605 5606 5608 5728 5810 5883 5894 5925 5928 5933 5934 59341 595 5970 6009 6237 6300 677 678 7040 7042 7043 7046 7048 7157 7248 7249 7416 7417 7419 776 801 805 808 810 823 824 83447 83593 8503 85417 8878 890 8900 891 894 8945 896 898 90550 9133 9134 91750 91860 9261 983 993
BLOODGR_EXPR	HUGO_BLOOD_GROUPS	A4GALT ABCB6 ABCG2 ABO ACHE ACKR1 AQP1 AQP3 ART4 B3GALNT1 BCAM BSG C4A C4B CD151 CD44 CD55 CD59 CD99 CR1 ERMAP FUT1 FUT3 GBGT1 GCNT2 GYPA GYPB GYPC GYPE ICAM4 KEL RHAG RHCE RHD SEMA7A SLC14A1 SLC29A1 SLC4A1 SMIM1 XG XK	28 43 358 360 420 682 720 721 960 966 977 1378 1604 2030 2523 2525 2532 2651 2993 2994 2995 2996 3386 3792 4059 4267 6005 6006 6007 6521 6563 7499 7504 8482 8706 9429 10058 26301 53947 114625 388588
COMP	KEGG_hsa04610_COMPLEMENT_SELECTED	C1QA C1QB C1QC C1R C1S C2 C3 C3AR1 C4A C4B C4BPA C4BPB C5 C5AR1 C6 C7 C8A C8B C8G C9 CD46 CD55 CD59 CFB CFD CFH CFHR1 CFHR2 CFHR3 CFHR4 CFHR5 CFI CLU CR1 CR1L CR2 ITGAM ITGAX ITGB2 MASP1 MASP2 MBL2 SERPING1 VSIG4 VTN	10747 10877 10878 11326 1191 1378 1379 1380 1604 1675 3075 3078 3080 3426 3684 3687 3689 4153 4179 5648 629 710 712 713 714 715 716 717 718 719 720 721 722 725 727 728 729 730 731 732 733 735 7448 81494 966
COAG	KEGG_hsa04610_COAGULATION_SELECTED	A2M BDKRB1 BDKRB2 CPB2 F10 F11 F12 F13A1 F13B F2 F2R F2RL2 F2RL3 F3 F5 F7 F8 F9 FGA FGB FGG KLKB1 KNG1 PLAT PLAU PLAUR PLG PROC PROCR PROS1 SERPINA1 SERPINA5 SERPINB2 SERPINC1 SERPIND1 SERPINE1 SERPINF2 TFPI THBD VWF	10544 1361 2147 2149 2151 2152 2153 2155 2157 2158 2159 2160 2161 2162 2165 2243 2244 2266 2 3053 3818 3827 462 5054 5055 5104 5265 5327 5328 5329 5340 5345 5624 5627 623 624 7035 7056 7450 9002

MET_EN_OXYD_PHOSPH	KEGG_hsa00190	AL9294102 ATP12A ATP4A ATP4B ATP5F1A ATP5F1B ATP5F1C ATP5F1D ATP5F1E ATP5MC1 ATP5MC1P5 ATP5MC2 ATP5MC3 ATP5ME ATP5MF ATP5MG ATP5PB ATP5PD ATP5PF ATP5PO ATP6AP1 ATP6V0A1 ATP6V0A2 ATP6V0A4 ATP6V0B ATP6V0C ATP6V0D1 ATP6V0D2 ATP6V0E1 ATP6V0E2 ATP6V1A ATP6V1B1 ATP6V1B2 ATP6V1C1 ATP6V1C2 ATP6V1D ATP6V1E1 ATP6V1E2 ATP6V1F ATP6V1G1 ATP6V1G2 ATP6V1G3 ATP6V1H COX10 COX11 COX15 COX17 COX4I1 COX4I2 COX5A COX5B COX6A1 COX6A2 COX6B1 COX6B2 COX6C COX6CP3 COX7A1 COX7A2 COX7A2L COX7B COX7B2 COX7C COX8A COX8C CYC1 LHPP MT-ATP6 MT-ATP8 MT-CO1 MT-CO2 MT-CO3 MT-CYB MT-ND1 MT-ND2 MT-ND3 MT-ND4 MT-ND4L MT-ND5 MT-ND6 NDUFA1 NDUFA10 NDUFA11 NDUFA2 NDUFA3 NDUFA4 NDUFA4L2 NDUFA5 NDUFA6 NDUFA7 NDUFA8 NDUFA9 NDUFAB1 NDUFB1 NDUFB10 NDUFB2 NDUFB3 NDUFB4 NDUFB5 NDUFB6 NDUFB7 NDUFB8 NDUFB9 NDUFC1 NDUFC2 NDUFS1 NDUFS2 NDUFS3 NDUFS4 NDUFS5 NDUFS6 NDUFS7 NDUFS8 NDUFV1 NDUFV2 NDUFV3 PPA1 PPA2 SDHA SDHB SDHC SDHD TCIRG1 UQCR10 UQCR11 UQCRB UQCRC1 UQCRC2 UQCRFS1 UQCRH UQCRHL UQCRQ ATP6 ATP8 COX1 COX2 COX3 CYTB ND1 ND2 ND3 ND4 ND4L ND5 ND6 NDUFA12 NDUFA13 NDUFB11 NDUFC2-KCTD14	100130247 10063 10312 10476 10632 10975 125965 126328 127124 1327 1329 1337 1339 1340 1345 1346 1347 1349 1350 1351 1352 1353 1355 1537 155066 170712 23545 245972 245973 27068 27089 29796 341947 374291 390424 440567 4508 4509 4512 4513 4514 4519 4535 4536 4537 4538 4539 4540 4541 4694 4695 4696 4697 4698 4700 4701 4702 4704 4705 4706 4707 4708 4709 4710 4711 4712 4713 4714 4715 4716 4717 4718 4719 4720 4722 4723 4724 4725 4726 4728 4729 4731 479 495 496 498 506 50617 509 513 51382 514 515 516 51606 517 518 521 522 523 525 526 527 528 529 533 534 535 537 539 5464 56901 6389 6390 6391 6392 64077 644310 7381 7384 7385 7386 7388 84701 8992 90423 9114 9167 9296 9377 9550 9551 100532726 51079 54539 55967
CP_CCYCLE_CCYCLE	KEGG_hsa04110	ABL1 AC0235121 ANAPC1 ANAPC10 ANAPC11 ANAPC13 ANAPC2 ANAPC4 ANAPC5 ANAPC7 ATM ATR BUB1 BUB1B BUB3 CCNA1 CCNA2 CCNB1 CCNB2 CCNB3 CCND1 CCND2 CCND3 CCNE1 CCNE2 CCNH CDC14A CDC14B CDC16 CDC20 CDC23 CDC25A CDC25B CDC25C CDC26 CDC27 CDC45 CDC6 CDC7 CDK1 CDK2 CDK4 CDK6 CDK7 CDKN1A CDKN1B CDKN1C CDKN2A CDKN2B CDKN2C CDKN2D CHEK1 CHEK2 CREBBP CUL1 DBF4 E2F1 E2F2 E2F3 E2F4 E2F5 EP300 ESPL1 FZR1 GADD45A GADD45B GADD45G GSK3B HDAC1 HDAC2 MAD1L1 MAD2L1 MAD2L2 MCM2 MCM3 MCM4 MCM5 MCM6 MCM7 MDM2 MYC ORC1 ORC2 ORC3 ORC4 ORC5 ORC6 PCNA PKMYT1 PLK1 PRKDC PTTG1 PTTG2 RAD21 RB1 RBL1 RBL2 RBX1 SFN SKP1 SKP2 SMAD2 SMAD3 SMAD4 SMC1A SMC1B SMC3 STAG1 STAG2 TFDP1 TFDP2 TGFB1 TGFB2 TGFB3 TP53 TTK WEE1 WEE2 YWHAB YWHAE YWHAG YWHAH YWHAQ YWHAZ ZBTB17	1017 1019 1021 1022 1026 1027 10274 1028 1029 1030 1031 1032 10393 10459 10735 10744 10912 10926 10971 1111 11200 1387 1647 1869 1870 1871 1874 1875 2033 23594 23595 246184 25 25847 27127 2810 2932 29882 29945 3065 3066 4085 4087 4088 4089 4171 4172 4173 4174 4175 4176 4193 4609 4616 472 494551 4998 4999 5000 5001 5111 51343 51433 51434 51529 5347 545 5591 5885 5925 5933 5934 595 64682 6500 6502 699 701 7027 7029 7040 7042 7043 7157 7272 728622 7465 7529 7531 7532 7533 7534 7709 8243 8317 8318 8379 8454 85417 8555 8556 8697 8881 890 8900 891 894 896 898 902 9088 9126 9133 9134 9184 9232 9700 983 990 991 993 994 995 996 9978

NEUTRO_DEGRAN	R-HSA-6798695	A1BG ABCA13 ACAA1 ACLY ACP3 ACTR10 ACTR1B ACTR2 ADA2 ADAM10 ADAM8 ADGRE3 ADGRE5 ADGRG3 AGA AGL AGPAT2 AHSG ALAD ALDH3B1 ALDOA ALDOC ALOX5 AMPD3 ANO6 ANPEP ANXA2 AOC1 AP1M1 AP2A2 APAF1 APEH APRT ARG1 ARHGAP45 ARHGAP9 ARL8A ARMC8 ARPC5 ARSA ARSB ASAH1 ATAD3B ATG7 ATP11A ATP11B ATP6AP2 ATP6V0A1 ATP6V0C ATP6V1D ATP8A1 ATP8B4 AZU1 B2M B4GALT1 BIN2 BPI BRI3 BST1 BST2 C1orf35 C3 C3AR1 C5AR1 C6orf120 CAB39 CALML5 CAMP CAND1 CANT1 CAP1 CAPN1 CAT CCT2 CCT8 CD14 CD177 CD300A CD33 CD36 CD44 CD47 CD53 CD55 CD58 CD59 CD63 CD68 CD93 CDA CDK13 CEACAM1 CEACAM3 CEACAM6 CEACAM8 CEP290 CFD CFP CHI3L1 CHIT1 CHRNB4 CKAP4 CLEC12A CLEC4C CLEC4D CLEC5A CMTM6 CNN2 COMMD3 COMMD9 COPB1 COTL1 CPNE1 CPNE3 CPPED1 CR1 CRACR2A CREG1 CRISP3 CRISPLD2 CSNK2B CST3 CSTB CTSA CTSB CTSC CTSD CTSG CTSH CTSS CTSZ CXCL1 CXCR1 CXCR2 CYB5R3 CYBA CYBB CYFIP1 CYSTM1 DBNL DDOST DDX3X DEFA1 DEFA1B DEFA4 DEGS1 DERA DGAT1 DIAPH1 DNAJC13 DNAJC3 DNAJC5 DNASE1L1 DOCK2 DOK3 DPP7 DSC1 DSG1 DSN1 DSP DYNC1H1 DYNC1LI1 DYNLL1 DYNLT1 EEF1A1 EEF2 ELANE ENPP4 EPX ERP44 FABP5 FAF2 FCAR FCER1G FCGR2A FCGR3B FCN1 FGL2 FGR FLG2 FOLR3 FPR1 FPR2 FRK FRMPD3 FTH1 FTL FUCA1 FUCA2 GAA GALNS GCA GDI2 GGH GHDC GLA GLB1 GLIPR1 GM2A GMFG GNS GOLGA7 GPI GPR84 GRN GSDMD GSN GSTP1 GUSB GYG1 HBB HEBP2 HEXB HGSNAT HK3 HLA-A HLA-B HLA-C HMGB1 HMOX2 HP HPSE HRNR HSP90AA1 HSP90AB1 HSPA1A HSPA1B HSPA6 HSPA8 HUWE1 HVCN1 IDH1 IGF2R ILF2 IMPDH1 IMPDH2 IQGAP1 IQGAP2 IRAG2 IST1 ITGAL ITGAM ITGAV ITGAX ITGB2 JUP KCMF1 KCNAB2 KPNB1 KRT1 LAIR1 LAMP1 LAMP2 LAMTOR1 LAMTOR2 LAMTOR3 LCN2 LGALS3 LILRA3 LILRB2 LILRB3 LPCAT1 LRG1 LRRC7 LTA4H LTF LYZ MAGT1 MAN2B1 MANBA MAPK1 MAPK14 MCEMP1 METTL7A MGAM MGST1 MIF MLEC MME MMP25 MMP8 MMP9 MNDA MOSPD2 MPO MS4A3 MVP NAPRT NBEAL2 NCKAP1L NCSTN NDUFC2 NEU1 NFAM1 NFASC NFKB1 NHLRC3 NIT2 NME2 NPC2 NRAS OLFM4 OLR1 ORM1 ORM2 ORMDL3 OSCAR OSTF1 P2RX1 PA2G4 PADI2 PAFAH1B2 PDAP1 PDXK PECAM1 PFKL PGAM1 PGLYRP1 PGM1 PGM2 PGRMC1 PIGR PKM PKP1 PLAC8 PLAU PLAUR PLD1 PLEKHO2 PNP PPBP PPIA PPIE PRCP PRDX4 PRDX6 PRG2 PRG3 PRKCD PRSS2 PRSS3 PRTN3 PSAP PSEN1 PSMA2 PSMA5 PSMB1 PSMB7 PSMC2 PSMC3 PSMD1 PSMD11 PSMD12 PSMD13 PSMD14 PSMD2 PSMD3 PSMD6 PSMD7 PTAFR PTGES2 PTPN6 PTPRB PTPRC PTPRJ PTPRN2 PTX3 PYCARD	1 100049587 10043 10075 10092 10097 101 10120 10159 102 10213 10288 10312 10321 10326 10383 10394 10396 10450 10487 10493 10533 10549 10555 10562 10576 10577 10694 1075 10788 1084 10855 10857 10875 1088 10890 10970 11001 11010 11025 11026 11031 1116 1118 11240 11314 11322 11333 1143 114790 116844 12 121260 124583 126014 1265 127829 1315 1378 138050 140885 1432 1460 1471 1476 150372 1508 1509 1511 1512 1520 1522 1535 1536 154664 158747 160364 1604 161 1650 1654 1667 1669 1675 170482 1727 1729 175 1774 1778 178 1794 1823 1828 1832 1915 1938 196527 197 1991 1992 199675 201294 203068 2040 210 2171 2204 2207 221 2212 2215 2219 222487 226 2268 22875 22918 22931 230 23071 23114 23191 23197 23200 23218 23250 23317 23352 23385 23406 23412 2352 23526 2357 2358 23593 23601 240 2444 2495 2512 2517 2519 2547 2548 25797 25798 25801 258010 25840 25852 2588 26 26578 2665 2683 2717 27180 272 2720 2760 2799 2821 28956 2896 28988 290 29099 29108 2919 2934 2950 2990 2992 29952 30 302 3043 3071 3074 3101 3105 3106 3107 3146 3163 317 3240 326624 327 3303 3304 3310 3312 3320 3326 338339 340061 3417 3482 353 353189 353376 3577 3579 3608 3614 3615 3683 3684 3685 3687 3689 3728 374395 374403 383 3837 3848 387 387263 387921 388697 388698 3903 391 3916 3920 3934 3958 401258 4033 4048 4057 4069 410 411 4125 4126 4257 427 4282 4311 4317 4318 4332 4353 441864 4680 47 4718 4758 4790 4831 4860 4893 4973 5004 5005 5023 5036 5049 51071 51125 51143 51209 51316 51382 51411 51552 51646 51719 5175 51806 51816 5199 5211 5223 5236 5265 5269 527 5273 5284 5315 5317 5328 5329 5337 535 53831 53916 53917 54472 54509 5473 5476 5478 54918 55 55004 55276 55313 5547 5553 55754 5580 55832 55860 5594 5611 5645 5646 5657 566 5660 5663 567 56729 5683 5686 56888 5689 5695 56954 5701 5702 57020 5707 5708 5709 57126 5713 57153 5717 5718 5719 5724 57554 5768 5777 57826 5787 5788 5795 5799 5806 5834 5836 58485 5864 5869 5870 5873 5878 5879 5906 5908 5912 6036 6037 6093 6278 6279 6280 6282 6283 6286 6317 634 6386 6402 64114 64333 64386 6515 6518 6556 6590 6616 6709 671 6727 6793 683 6836 684 6947 6993 7077 7097 7130 7133 718 719 7226 7276 728 728358 7305 7414 7415 7520 7879 79169 79792 79888 79895 79930 79980 80142 80184
