# Supplementary material for: Transcriptomic Signature Differences Between SARS-CoV-2 and Influenza Virus Infected Patients
Source: Front Immunol. 2021 May 31;12:666163. doi: 10.3389/fimmu.2021.666163 (PMC8202013; doi:10.3389/fimmu.2021.666163)
Supplement: Supplementary Table 3 — Composition of genes sets for supplementary figures. [file Table_3.pdf]

pathname	codinc	symbol	entrez
BCELL_PROL	GO:0030890	ADA ATAD5 BCL2 BCL6 BST1 CARD11 CD320 CD38 CD40 CD74 CD81 CDKN1A CHRNB2 CLCF1 FCRL3 GPR183 IL13 IL2 IL21 IL4 IL5 IL7 IRS2 MEF2C MIF NCKAP1L NFATC2 PELI1 PTPRC SASH3 SLC39A10 TFRC TICAM1 TIRAP TLR4 TLR9 TNFRSF13C TNFRSF4 TNFSF13B VAV3 WNT3A	100 1026 10451 10673 1141 114609 115352 115650 148022 1880 23529 3071 3558 3565 3567 3574 3596 4208 4282 4773 51293 54106 54440 57162 57181 5788 59067 596 604 683 7037 7099 7293 79915 84433 8660 89780 952 958 972 975
BCELL_DIFF	GO:0045579	ATP11C BAD BTK CD27 IL7 INPP5D MMP14 NCKAP1L PCID2 PPP2R3C STAT5B SYK XBP1	286410 3071 3574 3635 4323 55012 55795 572 6777 6850 695 7494 939
BCELL_SWITCH	GO:0048304	ATAD5 CD28 CD40 IL2 IL4 MLH1 MSH2 PAXIP1 PTPRC TBX21	22976 30009 3558 3565 4292 4436 5788 79915 940 958
TCELL1_PROL	GO:0042102	AGER AIF1 ANXA1 CARD11 CCDC88B CCL19 CCL5 CCR2 CD1D CD209 CD24 CD274 CD276 CD28 CD3E CD4 CD40LG CD46 CD55 CD6 CD70 CD80 CD81 CD86 CLECL1 CORO1A DNAJA3 EBI3 EFNB1 EPO FADD FOXP3 GPAM HAVCR2 HES1 HHLA2 HLA-A HLA-DMB HLA-DPA1 HLA-DPB1 HLA-E HMGB1 ICOSLG IGF1 IGF2 IGFBP2 IL12A IL12B IL12RB1 IL15 IL18 IL1A IL1B IL2 IL21 IL23A IL23R IL27RA IL2RA IL4 IL6 IL6ST JAK3 LEP LGALS9 LILRB2 MIR21 MIR30B NCK1 NCK2 NCKAP1L PDCD1LG2 PNP PRKCQ PTPN22 PTPRC PYCARD RASAL3 RIPK2 RPS3 SASH3 SELENOK SHH SLC7A1 SPTA1 STAT5B SYK TFRC TGFBR2 TMIGD2 TNFRSF13C TNFSF13B TNFSF4 TNFSF9 TRAF6 VCAM1 VTCN1 XCL1 ZAP70 ZP3 ZP4	100133941 10148 10288 10673 11148 11151 115650 126259 149233 160365 1604 177 1947 199 2056 23308 26191 283234 29108 29126 301 3071 30835 3105 3109 3113 3115 3133 3146 3280 3479 3481 3485 3552 3553 3558 3559 3565 3569 3572 3592 3593 3594 3600 3606 3718 3952 3965 406991 407030 4179 4690 4860 50943 51561 54440 5588 57678 57829 5788 58515 59067 6188 6352 6363 6375 6469 64926 6541 6708 6777 6850 7037 7048 7189 7292 729230 7412 7535 7784 79679 80380 80381 8440 84433 84868 8744 8767 8772 9093 912 916 920 923 940 941 942 9466 959 970 975
TCELL1_DIFF	GO:0045591	BCL6 BTN2A2 CD46 DUSP10 FOXP3 HLA-G IFNG IL2 LGALS9 LILRB2 LILRB4 SOCS1 SOX12 VSIR	10288 10385 11006 11221 3135 3458 3558 3965 4179 50943 604 64115 6666 8651
TCELL1_SEL	GO:0043368	BATF BCL11B BCL2 CD3D CD3E CD3G CD74 CTSL CYLD DOCK2 FOXN1 FOXP3 IL12B IL12RB1 IL23A IL23R IL6 IRF4 ITPKB LOXL3 LY9 MTOR PTPN2 PTPRC SHH SLAMF6 SPN SRF STAT3 STAT6 STK11 TBX21 THEMIS TOX ZAP70 ZFPM1	10538 114836 149233 1514 1540 161882 1794 2475 30009 3569 3593 3594 3662 3707 387357 4063 50943 51561 5771 5788 596 6469 64919 6693 6722 6774 6778 6794 7535 8456 84695 915 916 917 972 9760
TCELL2_TOX	GO:0001916	AZGP1 B2M CD1A CD1B CD1C CD1D CD1E CYRIB FADD HLA-A HLA-B HLA-E HLA-F HLA-G HLA-H IL12A IL12B IL12RB1 IL23A IL23R MR1 NECTIN2 P2RX7 PTPRC PVR SLC22A13 STX7 XCL1	149233 3105 3106 3133 3134 3135 3136 3140 3592 3593 3594 5027 51561 51571 563 567 5788 5817 5819 6375 8417 8772 909 910 911 912 913 9390
TH1	KEGG_hsa04658_TH1_SELECTED	DLL1 DLL3 DLL4 IFNG IFNGR1 IFNGR2 IL12A IL12B IL12RB1 IL12RB2 JAK1 JAK2 MAML1 MAML2 MAML3 NFKB1 NOTCH3 RBPJ RBPJL RELA STAT1 STAT4 TYK2	10683 11317 28514 3458 3459 3460 3516 3592 3593 3594 3595 3716 3717 4790 4854 54567 55534 5970 6772 6775 7297 84441 9794
TH2	KEGG_hsa04658_TH2_SELECTED	GATA3 IL13 IL2 IL2RA IL2RB IL2RG IL4 IL4R IL5 JAG1 JAG2 JAK3 MAF NOTCH1 NOTCH2 STAT5A STAT5B STAT6	182 2625 3558 3559 3560 3561 3565 3566 3567 3596 3714 3718 4094 4851 4853 6776 6777 6778
