# Supplementary material for: Transcriptomic Signature Differences Between SARS-CoV-2 and Influenza Virus Infected Patients
Source: Front Immunol. 2021 May 31;12:666163. doi: 10.3389/fimmu.2021.666163 (PMC8202013; doi:10.3389/fimmu.2021.666163)
Supplement: Supplementary Table 4 — Differentially expressed genes as markers of COVID-19. [file Table_4.pdf]

symbol	TUBE_INFL.log2FoldChange	TUBE_INFL.padj	TUBE_HLTY.log2FoldChange	TUBE_HLTY.padj	OXY1_INFL.log2FoldChange	OXY1_INFL.padj	OXY1_HLTY.log2FoldChange	OXY1_HLTY.padj	OXY0_INFL.log2FoldChange	OXY0_INFL.padj	OXY0_HLTY.log2FoldChange	OXY0_HLTY.padj
HDAC6	-0.46	9.7E-06	-0.56	1.1E-09	-0.30	1.2E-04	-0.39	3.0E-08	-0.36	2.6E-05	-0.45	4.0E-08
UNK	-0.59	8.0E-08	-0.60	3.3E-10	-0.28	1.2E-03	-0.27	2.3E-04	-0.26	4.9E-03	-0.26	3.1E-03
ZNF384	-0.30	4.6E-04	-0.26	6.0E-04	-0.22	1.2E-03	-0.16	5.8E-03	-0.40	7.6E-08	-0.34	5.2E-07
TGFBI	-1.47	6.3E-07	-1.49	7.5E-09	-0.67	2.2E-03	-0.69	5.3E-04	-0.63	7.2E-03	-0.63	5.1E-03
UBAP2L	-0.42	2.2E-04	-0.41	5.1E-05	-0.25	6.3E-03	-0.23	7.0E-03	-0.31	1.7E-03	-0.28	3.2E-03
DIAPH1	-0.43	4.1E-03	-0.46	6.4E-04	-0.33	4.7E-03	-0.33	1.5E-03	-0.41	1.2E-03	-0.40	7.8E-04

symbol	TUBE_INFL.log2FoldChange	TUBE_INFL.padj	TUBE_HLTY.log2FoldChange	TUBE_HLTY.padj	OXY1_INFL.log2FoldChange	OXY1_INFL.padj	OXY1_HLTY.log2FoldChange	OXY1_HLTY.padj	OXY0_INFL.log2FoldChange	OXY0_INFL.padj	OXY0_HLTY.log2FoldChange	OXY0_HLTY.padj
CDC6	1.50	6.3E-07	3.28	1.9E-32	1.37	5.8E-09	3.14	4.0E-47	0.92	2.5E-04	2.71	1.8E-27
IGLV3-25	3.22	7.0E-08	4.66	2.9E-19	4.19	7.1E-20	5.60	5.1E-43	3.28	4.1E-10	4.71	1.6E-23
JCHAIN	2.72	3.5E-09	3.97	8.9E-23	2.87	7.3E-16	4.10	5.2E-39	2.43	1.6E-09	3.67	8.1E-24
RRM2	1.55	5.3E-06	3.23	3.4E-26	1.57	4.1E-09	3.22	6.3E-42	1.08	1.4E-04	2.77	8.1E-24
IGLV3-19	4.10	1.1E-11	5.40	4.4E-24	3.93	6.1E-17	5.22	2.6E-36	2.09	3.0E-05	3.44	1.0E-12
IGLV3-1	2.65	6.5E-07	3.68	2.9E-15	3.91	6.2E-21	4.91	1.3E-40	2.35	3.2E-07	3.38	2.9E-15
SDC1	2.83	3.0E-06	4.85	1.5E-18	3.51	2.2E-13	5.48	2.6E-35	2.78	1.5E-07	4.78	3.5E-21
IGLV3-10	3.36	1.5E-07	4.86	3.3E-18	3.86	6.9E-15	5.33	3.6E-34	2.99	4.9E-08	4.50	9.6E-19
IGHV1-24	3.53	6.9E-07	4.52	4.5E-13	5.03	2.2E-19	5.97	2.3E-33	3.52	1.9E-08	4.49	8.1E-15
BHLHA15	2.38	1.9E-06	4.05	2.4E-18	2.81	1.5E-12	4.44	5.2E-32	2.04	2.1E-06	3.71	6.8E-18
IGLC2	2.60	2.1E-07	4.04	3.1E-20	2.68	4.1E-12	4.10	2.9E-33	1.67	5.2E-05	3.13	3.6E-15
BUB1	1.67	2.2E-06	2.93	1.2E-20	1.63	3.2E-09	2.88	1.7E-31	1.30	1.3E-05	2.57	3.0E-19
TXNDC5	1.91	5.0E-07	2.59	6.4E-15	2.25	4.0E-14	2.92	5.9E-29	1.86	2.1E-08	2.53	1.0E-16
CDC25A	1.46	1.7E-04	3.56	1.7E-22	1.48	1.3E-06	3.55	5.9E-34	0.79	8.4E-03	2.92	2.6E-18
CAV1	2.34	2.6E-07	3.33	2.4E-16	2.53	2.0E-12	3.50	3.0E-26	2.21	2.7E-08	3.19	2.5E-17
IGHV4-34	4.32	1.6E-13	4.26	8.6E-17	3.64	1.6E-15	3.60	3.7E-19	3.35	1.4E-10	3.30	2.3E-12
HJURP	1.12	1.1E-04	2.46	1.1E-20	1.15	3.9E-07	2.47	1.5E-32	0.74	2.0E-03	2.07	8.1E-18
IGKV3-20	2.77	8.8E-10	3.42	3.8E-18	2.64	4.0E-14	3.28	1.3E-26	1.60	1.9E-05	2.27	2.3E-10
MZB1	2.29	1.6E-06	3.63	7.0E-18	2.49	3.0E-11	3.81	8.3E-31	1.37	4.3E-04	2.75	1.1E-12
SHCBP1	1.27	1.0E-05	2.38	3.2E-20	1.13	5.3E-07	2.22	9.3E-28	0.92	1.5E-04	2.03	9.6E-18
IGHV3-30	2.89	2.8E-07	3.86	3.3E-15	3.06	2.1E-12	4.01	2.0E-25	2.43	4.6E-07	3.38	4.1E-14
MCM10	1.37	5.9E-04	3.33	7.8E-19	1.57	7.6E-07	3.50	6.5E-31	0.96	2.9E-03	2.93	2.8E-17
GMNN	1.10	1.9E-07	1.88	1.9E-23	0.77	2.5E-06	1.54	2.1E-25	0.53	2.6E-03	1.30	3.9E-14
IGLV2-14	2.30	6.5E-07	3.26	1.2E-15	2.70	7.5E-14	3.63	4.9E-30	1.24	8.2E-04	2.23	2.1E-09
IGKV1-5	2.24	5.8E-06	3.88	1.2E-18	2.38	9.5E-10	3.98	6.4E-31	0.98	7.9E-03	2.69	2.1E-11
CDC45	1.29	1.6E-04	2.63	3.4E-17	1.53	2.1E-08	2.84	2.8E-30	0.89	1.6E-03	2.23	8.0E-15
IGKV1-12	2.06	4.3E-06	3.33	5.6E-17	2.21	3.1E-10	3.44	1.7E-28	1.30	4.4E-04	2.57	1.4E-12
PYCR1	2.03	6.9E-07	2.88	1.7E-15	2.26	2.0E-12	3.08	5.1E-27	1.38	5.7E-05	2.24	1.9E-11
IGLV1-47	2.33	7.8E-07	3.74	3.3E-19	2.07	1.4E-08	3.47	1.6E-26	1.26	6.8E-04	2.74	6.1E-13
IGLV1-40	1.59	7.8E-04	3.44	3.9E-15	2.06	9.3E-08	3.82	1.5E-28	1.56	1.5E-04	3.36	6.4E-17
IGKV3-15	2.19	1.9E-06	2.75	6.7E-12	2.73	4.0E-14	3.26	7.5E-25	2.02	3.3E-07	2.58	3.3E-12
CCNA2	0.94	2.2E-03	2.34	2.8E-16	1.15	2.7E-06	2.51	1.7E-28	0.87	8.2E-04	2.25	1.1E-17
IGLV2-8	2.82	8.9E-08	4.29	1.2E-20	1.97	7.7E-07	3.45	6.1E-22	1.63	1.3E-04	3.15	5.6E-14
GGH	1.94	9.9E-09	2.54	5.9E-18	1.62	5.7E-10	2.22	1.2E-21	1.22	1.9E-05	1.83	1.4E-11
NEK2	1.41	1.1E-05	2.68	1.7E-19	1.21	1.6E-06	2.47	1.3E-24	0.87	1.0E-03	2.14	6.2E-15
NCAPG	1.70	2.5E-05	3.21	1.1E-18	1.50	1.8E-06	3.00	3.2E-25	1.08	1.1E-03	2.60	7.2E-15
CDK1	1.51	2.5E-04	3.52	1.2E-19	1.22	1.4E-04	3.20	9.7E-25	0.91	5.1E-03	2.93	2.3E-16
TTK	1.71	1.5E-05	2.98	2.2E-16	1.72	3.9E-08	2.97	1.0E-23	1.26	1.3E-04	2.54	5.2E-14
IGLV2-23	1.93	3.6E-05	3.25	5.5E-15	2.32	3.4E-10	3.60	3.6E-28	1.05	3.4E-03	2.42	2.1E-10
SKA3	1.97	3.7E-06	3.26	5.8E-17	1.79	8.9E-08	3.08	3.5E-22	1.42	6.6E-05	2.73	4.7E-14
IGKV4-1	1.95	5.9E-05	3.12	4.2E-13	2.54	5.6E-11	3.66	5.6E-27	1.48	2.5E-04	2.66	2.2E-11
TYMS	1.05	1.9E-03	2.40	1.3E-14	1.41	2.7E-07	2.70	3.8E-28	0.89	1.8E-03	2.21	1.1E-14
IGLV3-16	2.83	6.1E-07	3.25	4.1E-11	3.28	2.1E-13	3.68	1.1E-20	2.60	1.3E-07	3.02	4.3E-11
IGKC	1.86	5.3E-05	3.18	6.8E-15	2.15	3.3E-09	3.43	1.2E-26	1.18	1.4E-03	2.52	1.7E-11

symbol	TUBE_INFL.log2FoldChange	TUBE_INFL.padj	TUBE_HLTY.log2FoldChange	TUBE_HLTY.padj	OXY1_INFL.log2FoldChange	OXY1_INFL.padj	OXY1_HLTY.log2FoldChange	OXY1_HLTY.padj	OXY0_INFL.log2FoldChange	OXY0_INFL.padj	OXY0_HLTY.log2FoldChange	OXY0_HLTY.padj
GLDC	1.81	3.3E-05	2.63	1.1E-11	2.25	8.6E-11	3.03	8.5E-23	1.84	1.2E-06	2.63	1.8E-13
TRIP13	1.30	3.6E-05	2.32	3.7E-16	1.31	1.2E-07	2.31	2.4E-24	0.87	8.4E-04	1.89	6.1E-13
FABP5	1.14	2.2E-06	2.01	8.7E-21	0.71	1.3E-04	1.58	8.9E-21	0.59	2.9E-03	1.46	1.0E-13
IGKV1-17	1.96	2.2E-04	2.98	1.8E-10	3.16	7.7E-14	4.14	1.9E-28	1.41	9.2E-04	2.47	1.3E-08
IGLV4-69	2.38	4.1E-07	3.17	1.1E-14	2.43	3.3E-11	3.20	3.4E-23	1.37	3.2E-04	2.19	6.0E-09
IGLV9-49	2.32	9.7E-06	2.84	4.7E-10	3.14	4.0E-14	3.63	2.5E-23	2.09	3.1E-06	2.62	7.7E-10
IGKV1D-33	1.37	4.7E-03	3.06	3.9E-11	2.44	4.1E-09	3.99	1.8E-27	1.63	1.9E-04	3.23	4.7E-14
CDCA3	1.38	2.2E-05	2.22	1.9E-14	1.53	2.5E-09	2.34	2.0E-24	0.87	9.5E-04	1.72	1.1E-10
IGKV2D-29	2.58	2.5E-06	2.69	1.2E-08	3.64	3.5E-17	3.74	7.8E-23	2.07	8.0E-06	2.19	5.8E-07
ZWINT	1.30	1.9E-05	1.96	2.8E-13	1.49	4.9E-10	2.13	1.3E-23	0.91	3.4E-04	1.57	2.5E-10
MELK	1.48	1.2E-04	2.93	1.2E-16	1.27	2.3E-05	2.71	1.9E-21	1.06	8.4E-04	2.52	1.4E-14
DTL	1.14	1.8E-03	2.53	1.2E-13	1.47	6.8E-07	2.81	6.6E-25	0.98	1.3E-03	2.36	7.2E-14
IGHV4-39	2.34	5.3E-06	2.74	8.5E-10	2.95	2.6E-13	3.33	1.1E-20	2.10	1.7E-06	2.50	1.6E-09
MYBL2	2.01	2.1E-06	2.95	2.4E-15	1.89	9.4E-09	2.81	5.8E-22	1.10	1.2E-03	2.06	1.5E-09
IGLV3-9	2.05	8.9E-04	3.53	2.4E-10	2.14	1.2E-05	3.57	1.1E-15	3.39	2.2E-09	4.78	2.5E-20
DLGAP5	1.42	5.3E-04	2.97	2.9E-15	1.42	1.1E-05	2.94	1.4E-22	1.06	1.5E-03	2.62	4.7E-14
UCHL1	2.96	1.2E-06	4.98	3.0E-18	2.19	4.2E-06	4.19	2.4E-19	1.40	3.0E-03	3.49	5.1E-11
CCNB1	1.52	1.9E-06	2.01	6.2E-13	1.57	3.1E-10	2.05	1.8E-20	1.07	6.6E-05	1.56	1.4E-09
CDCA2	1.51	1.5E-04	2.80	2.3E-14	1.58	6.7E-07	2.82	1.1E-21	1.14	5.9E-04	2.42	1.0E-12
TNFRSF17	2.49	4.0E-07	2.95	4.8E-12	2.54	4.2E-11	2.98	1.4E-18	1.89	6.3E-06	2.34	2.8E-09
PBK	1.89	1.2E-04	4.14	5.6E-17	1.53	7.1E-05	3.77	2.0E-19	1.28	1.2E-03	3.57	2.4E-14
CENPA	1.56	5.0E-05	2.83	3.4E-15	1.55	4.5E-07	2.79	2.8E-21	0.99	1.6E-03	2.27	1.4E-11
EXO1	1.66	1.6E-05	2.30	1.2E-11	1.85	1.3E-09	2.48	1.7E-19	1.46	8.2E-06	2.11	2.4E-11
IGKV3-11	1.86	9.4E-06	2.30	3.3E-10	2.34	9.7E-13	2.75	2.1E-21	1.41	5.9E-05	1.84	4.8E-08
E2F8	1.06	6.9E-03	2.85	3.8E-13	1.49	6.7E-06	3.19	1.3E-23	0.97	3.2E-03	2.74	6.9E-14
IGKV1-27	2.93	2.6E-07	3.69	9.1E-14	2.89	7.3E-11	3.62	2.0E-20	1.37	1.8E-03	2.17	1.3E-06
IGKV2D-40	2.13	7.3E-05	2.65	1.8E-08	3.17	2.1E-13	3.64	1.5E-21	2.00	1.6E-05	2.50	1.6E-08
IGKV3D-11	1.73	7.1E-05	2.36	7.5E-10	2.33	1.7E-11	2.92	1.0E-21	1.50	4.7E-05	2.12	2.5E-09
CCNB2	1.69	7.6E-06	2.65	1.6E-15	1.45	8.0E-07	2.40	7.7E-20	0.93	1.9E-03	1.93	3.2E-10
IGHA1	1.58	2.3E-03	3.23	1.8E-11	2.35	4.3E-08	3.90	1.4E-24	1.15	4.9E-03	2.82	2.3E-10
IGKV1-9	1.72	5.5E-04	2.71	1.3E-09	2.55	2.5E-10	3.47	2.3E-22	1.59	1.6E-04	2.56	7.1E-10
LINC01484	1.87	1.8E-05	3.24	2.6E-15	1.66	1.8E-06	3.00	9.3E-19	1.14	1.3E-03	2.53	5.1E-11
IGLV4-60	2.96	2.5E-05	3.97	1.4E-10	2.53	2.7E-06	3.55	5.8E-13	3.68	4.3E-09	4.64	6.5E-16
SEC11C	1.60	2.9E-09	1.62	3.2E-12	1.30	3.3E-10	1.32	6.1E-13	1.15	4.9E-07	1.17	4.4E-08
IGKV2D-28	2.36	2.3E-06	3.49	1.2E-15	1.94	4.1E-07	3.08	2.2E-19	1.00	6.8E-03	2.22	2.5E-08
IGHV3-13	1.98	1.4E-03	2.12	2.6E-05	3.75	5.3E-16	3.84	2.6E-22	4.81	6.1E-05	4.89	3.0E-06
IGLV7-43	2.31	4.0E-05	2.83	7.8E-09	3.38	4.4E-14	3.85	1.4E-22	1.09	6.6E-03	1.70	1.1E-04
SKA1	1.16	1.0E-07	1.61	5.1E-17	0.78	3.5E-06	1.22	8.5E-16	0.52	4.3E-03	0.95	7.5E-08
IGKV1-33	1.69	2.0E-03	2.62	1.4E-07	2.85	3.7E-10	3.68	9.2E-20	2.18	9.6E-06	3.03	1.0E-10
KIF11	1.47	2.1E-05	1.93	2.3E-10	1.56	1.1E-08	1.99	2.1E-16	1.36	5.5E-06	1.80	2.0E-10
IGHV3-20	2.65	6.2E-05	3.23	2.3E-08	3.89	2.1E-13	4.43	3.6E-21	1.63	1.7E-03	2.25	2.2E-05
IGHV4-28	1.50	2.6E-03	2.71	4.7E-09	2.42	5.5E-09	3.53	2.9E-21	1.50	4.3E-04	2.68	5.4E-10
GINS2	1.52	1.3E-05	2.27	1.6E-13	1.44	1.2E-07	2.18	4.1E-19	0.75	5.8E-03	1.52	7.8E-08
AURKA	0.87	3.1E-04	1.41	4.7E-11	1.01	1.1E-07	1.53	2.7E-19	0.67	8.6E-04	1.20	1.4E-09

symbol	TUBE_INFL.log2FoldChange	TUBE_INFL.padj	TUBE_HLTY.log2FoldChange	TUBE_HLTY.padj	OXY1_INFL.log2FoldChange	OXY1_INFL.padj	OXY1_HLTY.log2FoldChange	OXY1_HLTY.padj	OXY0_INFL.log2FoldChange	OXY0_INFL.padj	OXY0_HLTY.log2FoldChange	OXY0_HLTY.padj
NUSAP1	1.13	4.6E-05	1.70	6.3E-12	1.18	7.3E-08	1.72	1.3E-18	0.74	1.4E-03	1.29	1.6E-08
IGKV3D-20	1.77	2.8E-03	2.68	7.0E-07	2.28	3.1E-06	3.10	1.7E-12	3.31	3.2E-09	4.11	9.6E-16
BUB1B	1.52	2.3E-04	2.42	7.3E-11	1.72	1.6E-07	2.58	4.9E-18	1.14	8.0E-04	2.03	3.6E-09
IGHV3-43	2.11	4.8E-04	3.31	1.1E-09	2.86	4.2E-09	3.98	4.0E-20	1.37	3.4E-03	2.60	2.0E-07
IGHV3-33	1.54	3.0E-03	2.41	3.8E-07	2.80	1.3E-10	3.58	1.6E-20	1.59	4.3E-04	2.41	6.9E-08
IGHV1-46	1.63	3.4E-03	2.95	1.2E-08	2.33	4.9E-07	3.54	1.5E-17	2.05	4.4E-05	3.29	1.2E-11
IGHV3-21	1.72	7.3E-04	2.35	1.8E-07	2.68	6.5E-11	3.26	2.8E-19	1.51	3.2E-04	2.14	3.2E-07
UAP1	1.40	6.5E-08	0.98	6.6E-06	1.45	2.2E-13	1.02	3.1E-09	1.39	5.5E-10	0.96	1.7E-06
POLQ	0.97	3.5E-03	2.06	5.4E-11	1.19	9.2E-06	2.24	9.3E-19	0.81	3.5E-03	1.87	1.3E-10
PLAAT2	1.63	4.9E-04	3.16	2.7E-13	1.33	2.2E-04	2.85	3.7E-16	1.10	3.0E-03	2.66	4.0E-11
IGHV3-73	2.63	7.6E-05	3.60	9.5E-10	2.53	1.3E-06	3.46	2.1E-13	2.70	2.8E-06	3.64	3.1E-11
IGHV1-18	1.45	3.7E-03	2.32	4.3E-07	2.61	4.9E-10	3.38	9.1E-20	1.51	5.0E-04	2.32	6.9E-08
HASPIN	1.33	2.5E-04	2.11	1.2E-10	1.50	2.2E-07	2.25	1.8E-17	0.94	1.5E-03	1.72	1.7E-08
IGHV3-15	1.79	3.1E-04	2.16	6.0E-07	2.42	1.3E-09	2.75	6.5E-15	2.13	1.1E-06	2.47	1.8E-09
IGLV5-45	3.12	4.4E-07	3.37	2.7E-10	3.10	1.3E-10	3.34	4.4E-15	1.55	1.1E-03	1.84	9.3E-05
CHAC2	1.61	2.0E-06	1.77	1.7E-09	1.56	6.1E-09	1.70	6.2E-13	1.30	7.6E-06	1.44	1.2E-07
DEPDC1	1.81	5.5E-04	4.24	1.5E-14	1.17	2.8E-03	3.61	3.5E-14	1.18	4.1E-03	3.66	4.9E-12
IGHV1-69D	2.74	8.8E-05	2.27	9.8E-05	4.15	1.3E-13	3.66	1.1E-13	3.19	2.6E-07	2.68	2.2E-06
LMAN1	1.13	1.4E-05	1.09	1.7E-06	1.24	1.4E-09	1.17	1.1E-10	1.32	9.7E-09	1.23	3.9E-09
PTTG1	1.38	4.2E-07	1.72	5.4E-13	1.04	6.8E-07	1.37	2.3E-13	0.67	2.3E-03	1.01	3.7E-06
KCNN3	1.65	2.7E-05	2.01	6.1E-09	1.87	2.3E-09	2.19	2.5E-15	1.18	3.2E-04	1.52	1.8E-06
GINS1	1.17	3.5E-04	1.84	2.8E-10	1.23	1.7E-06	1.88	9.2E-16	0.86	1.5E-03	1.52	2.0E-08
CHEK1	1.05	3.5E-04	1.67	1.8E-10	1.11	1.9E-06	1.70	3.9E-16	0.71	3.1E-03	1.32	5.1E-08
DEPDC1B	1.36	7.0E-04	2.35	1.7E-10	1.29	4.5E-05	2.25	5.4E-14	1.24	2.8E-04	2.20	1.4E-10
IGKV6D-21	2.94	1.7E-05	4.03	2.6E-11	2.52	2.0E-06	3.59	1.2E-13	1.72	9.6E-04	2.90	1.5E-07
ITM2C	1.67	4.9E-07	1.14	4.8E-05	1.97	4.0E-14	1.43	3.8E-10	1.47	3.3E-07	0.91	4.3E-04
HSP90B1	0.87	1.4E-04	1.17	6.2E-09	0.90	5.1E-07	1.18	1.6E-13	0.78	7.2E-05	1.05	1.6E-08
OIP5	1.47	1.1E-04	2.33	2.0E-11	1.31	1.4E-05	2.15	4.7E-14	0.93	2.6E-03	1.79	3.9E-08
IGHV3-23	1.50	8.2E-04	2.16	6.9E-08	2.03	2.3E-08	2.63	3.2E-16	1.22	8.6E-04	1.88	4.6E-07
HMGB3	1.76	3.2E-08	1.68	5.3E-10	1.47	2.3E-09	1.38	1.2E-10	0.98	1.8E-04	0.90	2.4E-04
IGHV3-7	2.01	1.4E-04	2.43	1.4E-07	2.64	3.8E-10	3.01	6.8E-16	1.28	2.1E-03	1.71	5.3E-05
KIF15	1.02	6.6E-03	2.15	2.7E-09	1.31	2.8E-05	2.36	6.1E-16	0.98	2.2E-03	2.07	8.5E-10
IGKV5-2	2.34	2.7E-05	1.98	2.2E-05	2.84	1.6E-10	2.47	1.5E-10	2.59	1.4E-07	2.21	7.9E-07
ENSG00000216775.3	2.71	4.4E-07	2.76	1.6E-09	2.25	1.2E-07	2.30	5.9E-10	1.90	2.6E-05	1.95	3.8E-06
IGHV1-2	1.52	3.5E-03	2.22	3.4E-06	2.67	1.2E-09	3.27	5.5E-17	1.35	1.8E-03	2.02	6.1E-06
XBP1	0.75	3.4E-05	0.75	2.5E-06	0.87	7.1E-10	0.84	1.3E-11	0.70	6.9E-06	0.68	3.7E-06
IGHV3-53	1.62	1.1E-03	1.83	2.3E-05	2.47	9.5E-10	2.64	1.7E-13	1.91	1.2E-05	2.09	4.6E-07
CKAP2L	0.99	8.3E-03	2.23	1.8E-09	1.17	1.8E-04	2.33	1.1E-14	0.94	3.5E-03	2.13	7.7E-10
SPC25	1.90	3.3E-06	2.50	3.5E-12	1.33	2.5E-05	1.93	2.3E-11	0.91	4.5E-03	1.53	3.7E-06
UBE2T	1.50	1.1E-06	1.51	1.3E-08	1.33	4.7E-08	1.32	4.6E-10	1.08	3.9E-05	1.07	1.2E-05
GPRC5D	1.93	8.0E-04	2.49	9.4E-07	2.52	7.1E-08	3.03	4.3E-13	2.01	5.9E-05	2.53	1.4E-07
ASPM	1.32	4.5E-03	2.72	1.1E-09	1.34	3.5E-04	2.67	1.4E-13	1.22	1.9E-03	2.59	5.7E-10
IGHV3-69-1	2.76	1.9E-05	1.96	1.9E-04	3.80	1.5E-13	3.00	1.4E-11	2.32	2.3E-05	1.46	1.8E-03
MIXL1	1.74	1.3E-04	2.35	6.4E-09	1.78	1.0E-06	2.35	1.1E-12	1.29	6.3E-04	1.89	5.8E-07

symbol	TUBE_INFL.log2FoldChange	TUBE_INFL.padj	TUBE_HLTY.log2FoldChange	TUBE_HLTY.padj	OXY1_INFL.log2FoldChange	OXY1_INFL.padj	OXY1_HLTY.log2FoldChange	OXY1_HLTY.padj	OXY0_INFL.log2FoldChange	OXY0_INFL.padj	OXY0_HLTY.log2FoldChange	OXY0_HLTY.padj
ORC1	0.77	6.1E-03	1.34	3.2E-07	1.20	2.8E-07	1.71	4.2E-16	0.67	4.9E-03	1.20	8.7E-07
MANF	0.93	1.1E-05	1.13	1.3E-09	0.82	7.3E-07	1.00	1.1E-11	0.57	1.4E-03	0.75	1.3E-05
IGLVI-70	2.92	5.0E-05	3.55	2.1E-08	3.07	5.4E-08	3.70	5.4E-13	1.67	2.2E-03	2.38	3.7E-05
PRDX4	0.74	2.0E-03	1.41	1.8E-10	0.64	7.3E-04	1.26	3.4E-13	0.56	5.7E-03	1.19	5.6E-09
ENSG00000259772.6	1.49	1.1E-04	2.06	1.1E-09	1.36	5.9E-06	1.92	1.5E-12	0.94	2.3E-03	1.52	1.2E-06
IGHV2-26	1.99	3.7E-04	2.88	6.8E-09	2.02	4.7E-06	2.87	6.4E-13	1.44	1.3E-03	2.35	3.6E-07
NT5DC2	1.39	8.0E-05	1.61	1.7E-07	1.65	3.9E-09	1.83	1.2E-13	0.81	4.3E-03	1.01	3.0E-04
ERLEC1	0.70	9.7E-05	0.82	2.3E-07	0.65	3.3E-06	0.75	1.8E-09	0.72	4.1E-06	0.81	2.7E-08
CHPF	1.28	8.8E-05	1.30	4.9E-06	1.64	2.9E-10	1.62	1.4E-12	0.95	5.3E-04	0.94	3.2E-04
IGHV1-58	2.34	1.7E-04	1.88	2.3E-04	3.08	8.8E-10	2.61	1.5E-09	2.81	3.3E-07	2.34	2.7E-06
CIP2A	1.08	7.7E-04	1.54	9.3E-08	1.06	3.2E-05	1.48	1.5E-10	1.13	4.6E-05	1.56	6.3E-09
SPAG5	1.09	4.7E-05	1.20	2.6E-07	1.14	4.7E-08	1.24	2.6E-11	0.73	1.0E-03	0.82	1.2E-04
DERL3	1.66	1.6E-05	1.36	3.2E-05	2.01	3.3E-11	1.68	2.0E-10	1.17	2.3E-04	0.84	3.5E-03
NME1	1.34	2.0E-06	1.47	2.1E-09	1.10	5.3E-07	1.22	4.0E-10	0.61	7.2E-03	0.73	9.7E-04
HPDL	1.51	7.9E-04	2.05	3.2E-07	1.77	1.1E-06	2.27	4.7E-12	1.16	1.6E-03	1.69	6.3E-06
IGKV3-7	1.50	4.1E-03	2.50	3.6E-07	2.09	2.1E-06	3.00	1.6E-13	1.11	7.2E-03	2.10	4.6E-06
SPCS2	0.55	2.1E-07	0.45	8.3E-07	0.43	1.6E-07	0.33	7.6E-06	0.44	1.8E-06	0.33	1.1E-04
B4GALT2	1.28	3.7E-06	1.01	2.0E-05	1.38	1.7E-10	1.10	6.2E-09	0.89	1.3E-04	0.60	4.8E-03
IGLV4-3	2.55	1.9E-05	2.04	2.5E-05	3.00	6.1E-10	2.49	7.3E-10	1.89	1.6E-04	1.35	1.7E-03
SSR3	0.74	7.8E-07	0.54	3.3E-05	0.64	3.3E-08	0.44	2.5E-05	0.70	7.6E-08	0.49	4.9E-05
PSAT1	1.26	2.4E-06	1.25	6.7E-08	0.99	1.7E-06	0.97	1.1E-07	0.89	7.1E-05	0.87	4.1E-05
SLC16A14	2.41	1.0E-06	1.28	8.6E-04	2.56	1.4E-10	1.42	7.4E-06	2.30	1.2E-07	1.12	1.3E-03
DHFR	0.62	2.1E-03	1.06	4.2E-09	0.52	1.2E-03	0.93	8.9E-11	0.50	4.6E-03	0.89	8.2E-08
FAM83D	1.06	4.2E-04	1.19	6.1E-06	1.31	5.9E-08	1.40	3.7E-11	0.84	8.2E-04	0.94	9.5E-05
IGKV1D-8	1.61	1.1E-03	1.88	1.4E-05	2.11	1.7E-07	2.32	7.6E-11	1.53	2.8E-04	1.76	1.5E-05
CALU	0.49	3.3E-04	0.55	7.4E-06	0.53	1.0E-06	0.56	4.9E-09	0.50	4.0E-05	0.52	3.9E-06
FKBP11	0.84	1.4E-04	0.78	5.6E-05	1.04	1.4E-09	0.96	4.3E-10	0.64	5.5E-04	0.55	1.7E-03
ETFA	0.61	1.5E-07	0.56	3.3E-08	0.42	3.6E-06	0.37	8.6E-06	0.40	9.0E-05	0.33	5.2E-04
IGLL5	2.16	1.1E-04	1.73	2.0E-04	2.57	6.9E-09	2.10	4.2E-08	2.16	8.6E-06	1.65	1.3E-04
PARM1	1.51	5.8E-05	1.17	2.2E-04	1.68	1.6E-08	1.31	3.3E-07	1.55	2.0E-06	1.17	7.7E-05
CENPF	1.26	8.5E-05	1.49	1.1E-07	1.18	2.6E-06	1.39	5.8E-10	0.72	5.0E-03	0.94	2.5E-04
ENSG00000270933.1	1.70	4.2E-05	1.37	6.4E-05	1.97	5.1E-09	1.61	1.0E-08	1.33	1.4E-04	0.97	1.6E-03
ERCC6L	1.52	1.2E-03	2.06	9.5E-07	1.71	7.7E-06	2.20	3.0E-10	1.23	1.5E-03	1.75	9.9E-06
CCNE1	1.30	1.8E-05	1.10	2.3E-05	1.36	1.3E-08	1.13	4.3E-08	0.96	1.7E-04	0.73	1.8E-03
IRF4	1.19	1.8E-05	0.77	1.2E-03	1.27	5.3E-09	0.82	1.7E-05	1.25	2.6E-07	0.78	3.6E-04
POLE2	1.45	1.5E-04	1.65	7.4E-07	1.34	1.0E-05	1.52	2.0E-08	1.13	4.2E-04	1.31	2.2E-05
FER1L4	1.93	2.4E-05	1.69	1.3E-05	2.00	3.3E-08	1.73	3.0E-08	1.30	5.3E-04	1.01	2.5E-03
MCUR1	0.75	5.1E-08	0.44	1.9E-04	0.58	6.9E-08	0.26	6.4E-03	0.65	5.3E-08	0.33	2.9E-03
STIL	0.86	2.9E-03	1.19	6.7E-06	1.00	2.5E-05	1.27	2.2E-09	0.87	6.0E-04	1.14	3.3E-06
EIF2S1	0.39	7.2E-04	0.38	1.4E-04	0.37	2.2E-05	0.36	6.0E-06	0.49	6.2E-07	0.47	3.2E-07
FAM111B	1.74	2.8E-05	1.30	1.8E-04	1.76	9.0E-08	1.29	4.0E-06	1.64	5.3E-06	1.15	3.2E-04
POU2AF1	1.54	5.7E-06	1.12	1.1E-04	1.54	5.8E-09	1.09	2.0E-06	1.14	6.3E-05	0.68	7.1E-03
FEN1	0.67	1.8E-03	0.60	1.5E-03	1.01	3.8E-09	0.90	2.6E-09	0.70	1.8E-04	0.59	7.9E-04
IGLC1	1.88	1.9E-03	1.82	3.5E-04	2.65	7.1E-08	2.52	3.7E-09	1.94	1.8E-04	1.78	1.5E-04

symbol	TUBE_INFL.log2FoldChange	TUBE_INFL.padj	TUBE_HLTY.log2FoldChange	TUBE_HLTY.padj	OXY1_INFL.log2FoldChange	OXY1_INFL.padj	OXY1_HLTY.log2FoldChange	OXY1_HLTY.padj	OXY0_INFL.log2FoldChange	OXY0_INFL.padj	OXY0_HLTY.log2FoldChange	OXY0_HLTY.padj
MAD2L1	1.45	2.2E-06	0.88	6.7E-04	1.37	1.6E-08	0.77	1.7E-04	1.31	8.9E-07	0.69	2.9E-03
KIF18A	1.13	2.0E-03	1.71	2.9E-07	1.09	1.9E-04	1.63	1.7E-09	0.80	6.4E-03	1.36	1.1E-05
NUP37	0.60	1.7E-05	0.48	1.0E-04	0.47	1.7E-05	0.33	6.6E-04	0.65	1.1E-07	0.50	8.1E-06
NUF2	1.22	2.6E-03	1.83	8.2E-07	1.31	6.9E-05	1.87	6.5E-10	0.83	9.4E-03	1.43	3.3E-05
STMN1	1.06	3.2E-05	0.60	7.3E-03	1.21	2.0E-09	0.71	7.1E-05	1.08	1.3E-06	0.57	5.1E-03
SELENOS	0.31	8.0E-03	0.45	1.1E-05	0.36	1.3E-04	0.48	4.1E-09	0.33	1.6E-03	0.44	3.8E-06
PCNA	0.54	9.1E-04	0.38	6.2E-03	0.70	3.9E-08	0.54	3.0E-06	0.67	2.7E-06	0.50	2.3E-04
CENPU	1.22	1.6E-04	1.38	1.2E-06	1.09	1.7E-05	1.22	7.0E-08	0.75	3.9E-03	0.89	5.5E-04
TIPIN	1.01	8.9E-07	0.82	2.9E-06	0.72	7.4E-06	0.53	1.6E-04	0.69	7.5E-05	0.49	2.2E-03
PKHD1L1	1.29	1.9E-05	1.34	2.9E-07	0.93	6.1E-05	0.97	2.6E-06	0.77	1.8E-03	0.81	6.8E-04
ANKRD36BP2	1.42	3.4E-04	0.97	3.4E-03	1.49	2.3E-06	0.99	2.0E-04	1.82	2.3E-07	1.30	3.3E-05
IGHV3-66	1.50	7.5E-03	1.77	4.4E-04	2.44	5.2E-07	2.61	1.3E-09	1.32	4.2E-03	1.55	9.0E-04
OSTC	0.84	5.0E-06	0.48	3.0E-03	0.74	2.5E-07	0.37	4.8E-03	0.83	2.2E-07	0.44	2.7E-03
PLK4	1.24	6.4E-04	1.32	2.9E-05	1.27	1.2E-05	1.31	2.7E-07	1.01	8.3E-04	1.05	2.6E-04
PSMD14	0.55	2.3E-04	0.44	9.3E-04	0.57	1.2E-06	0.43	3.1E-05	0.56	1.7E-05	0.42	5.8E-04
NUCB2	1.23	6.1E-07	1.11	1.8E-07	0.64	6.4E-04	0.53	1.7E-03	0.72	4.8E-04	0.60	2.2E-03
CYCS	0.36	9.1E-03	0.52	1.5E-05	0.31	4.7E-03	0.45	2.3E-06	0.42	4.3E-04	0.55	5.3E-07
RPA3	0.84	6.0E-06	0.77	2.6E-06	0.51	4.5E-04	0.42	1.2E-03	0.63	1.0E-04	0.52	5.2E-04
SUV39H2	0.91	3.9E-04	0.83	1.8E-04	0.85	2.4E-05	0.74	2.3E-05	0.80	3.1E-04	0.66	9.1E-04
SEC61G	0.99	2.6E-06	0.82	8.0E-06	0.57	4.8E-04	0.40	7.3E-03	0.69	1.1E-04	0.51	2.8E-03
SKA2	0.59	1.6E-03	0.49	2.6E-03	0.50	5.3E-04	0.38	3.0E-03	0.79	7.2E-07	0.66	7.7E-06
HIBCH	0.51	1.1E-03	0.63	7.3E-06	0.38	2.0E-03	0.47	1.8E-05	0.43	1.6E-03	0.51	7.3E-05
TIMM17A	0.39	8.7E-04	0.36	5.2E-04	0.36	9.8E-05	0.31	1.5E-04	0.40	7.4E-05	0.35	2.6E-04
KDELR2	0.40	4.1E-04	0.43	1.3E-05	0.33	2.4E-04	0.34	1.2E-05	0.26	8.3E-03	0.27	3.3E-03
LARP1B	0.72	1.4E-03	0.53	7.0E-03	0.73	3.2E-05	0.51	9.7E-04	0.88	6.7E-06	0.64	3.3E-04
IER3IP1	0.44	8.8E-03	0.52	5.8E-04	0.39	3.5E-03	0.43	2.3E-04	0.59	6.3E-05	0.62	5.5E-06
ACAT1	0.68	3.2E-04	0.72	2.3E-05	0.48	1.1E-03	0.48	2.3E-04	0.50	2.2E-03	0.50	1.4E-03
ARHGAP42	1.04	9.5E-03	1.41	1.4E-04	1.04	1.4E-03	1.35	7.7E-06	0.90	7.2E-03	1.20	4.2E-04
TXNDC15	0.29	5.7E-03	0.23	9.3E-03	0.33	4.4E-05	0.26	2.1E-04	0.32	2.9E-04	0.26	2.3E-03
GFPT1	0.51	2.0E-03	0.45	2.6E-03	0.45	6.0E-04	0.36	2.4E-03	0.51	4.1E-04	0.41	2.7E-03
DNAJB9	0.75	1.3E-03	0.74	3.1E-04	0.48	7.0E-03	0.45	5.9E-03	0.66	7.7E-04	0.61	1.1E-03
HBS1L	0.51	7.6E-04	0.47	4.3E-04	0.35	3.2E-03	0.29	6.5E-03	0.42	1.4E-03	0.35	4.7E-03
NDUFAB1	0.45	3.1E-03	0.41	2.0E-03	0.36	2.7E-03	0.31	4.5E-03	0.39	2.7E-03	0.34	7.6E-03
