# Supplementary material for: Transcriptomic Signature Differences Between SARS-CoV-2 and Influenza Virus Infected Patients
Source: Front Immunol. 2021 May 31;12:666163. doi: 10.3389/fimmu.2021.666163 (PMC8202013; doi:10.3389/fimmu.2021.666163)
Supplement: Supplementary Figure 1 — Quality control. [file Image_1.pdf]

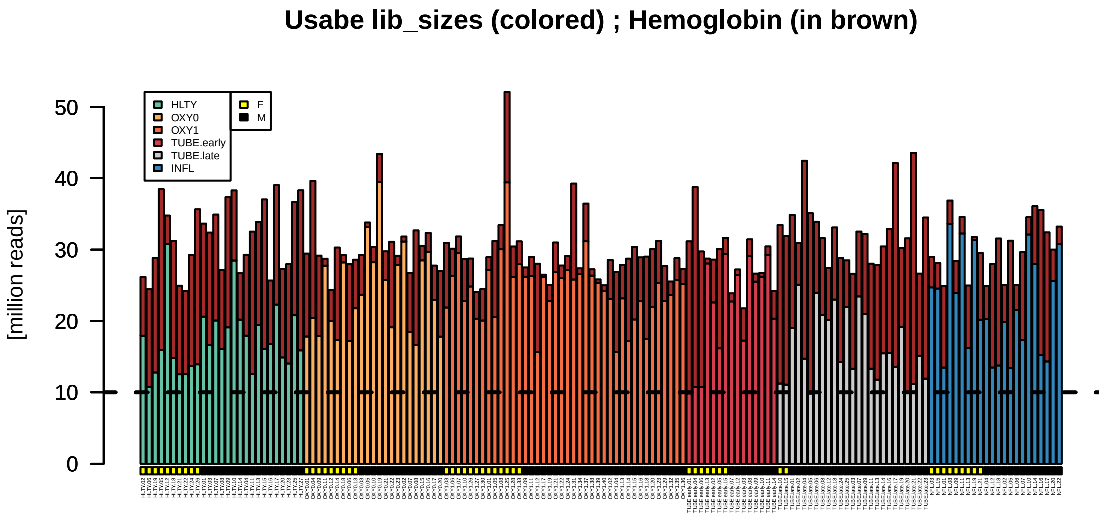
Usabe lib_sizes (colored) ; Hemoglobin (in brown)
[million reads]
0
10
20
30
40
50
HLTY
OXY0
OXY1
TUBE.early
TUBE.late
INFL
F
M

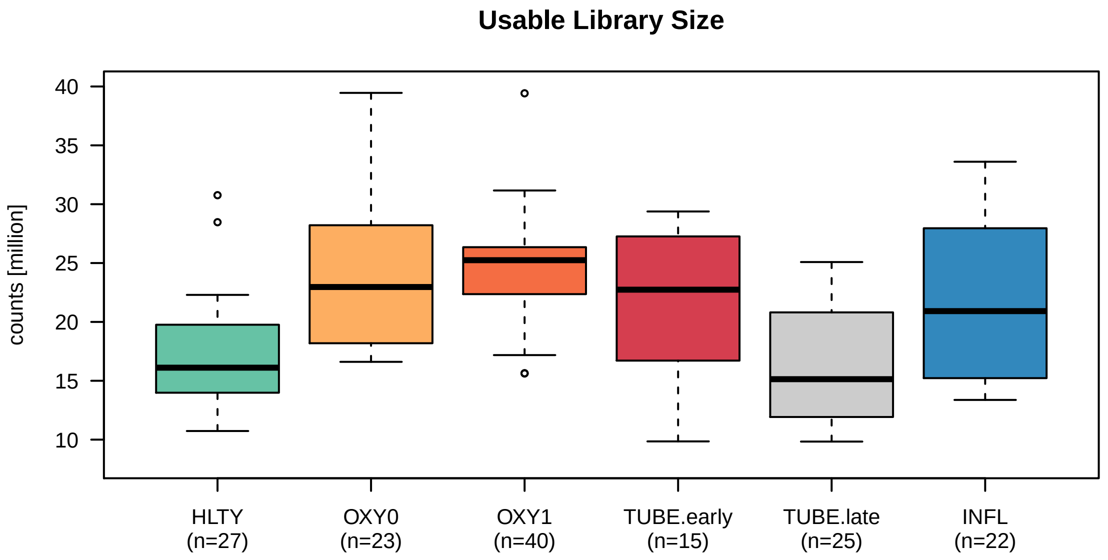
Usable Library Size
counts [million]
10
15
20
25
30
35
40
HLTY (n=27)
OXY0 (n=23)
OXY1 (n=40)
TUBE.early (n=15)
TUBE.late (n=25)
INFL (n=22)

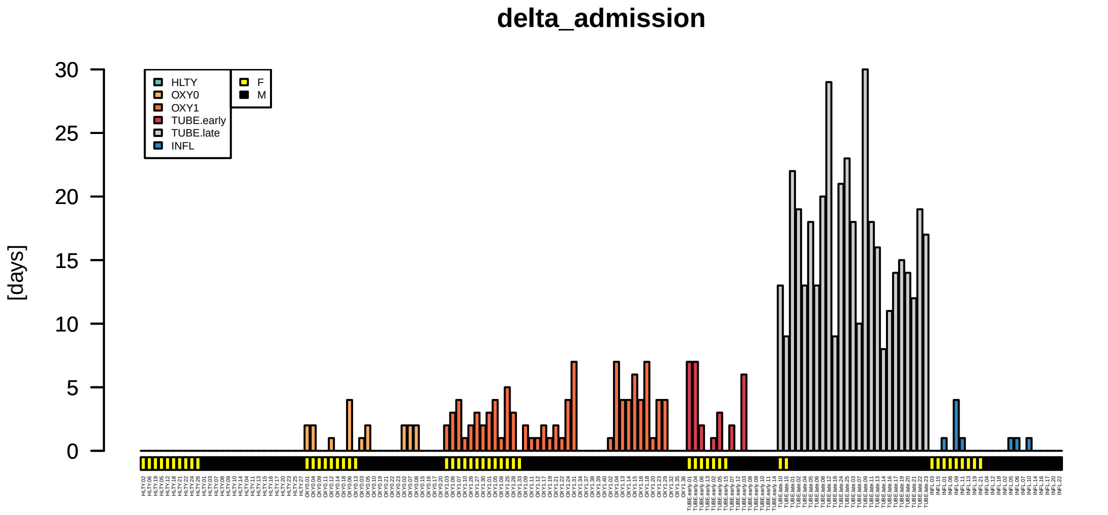
delta_admission
[days]
0
5
10
15
20
25
30
HLTY
OXY0
OXY1
TUBE.early
TUBE.late
INFL
F
M

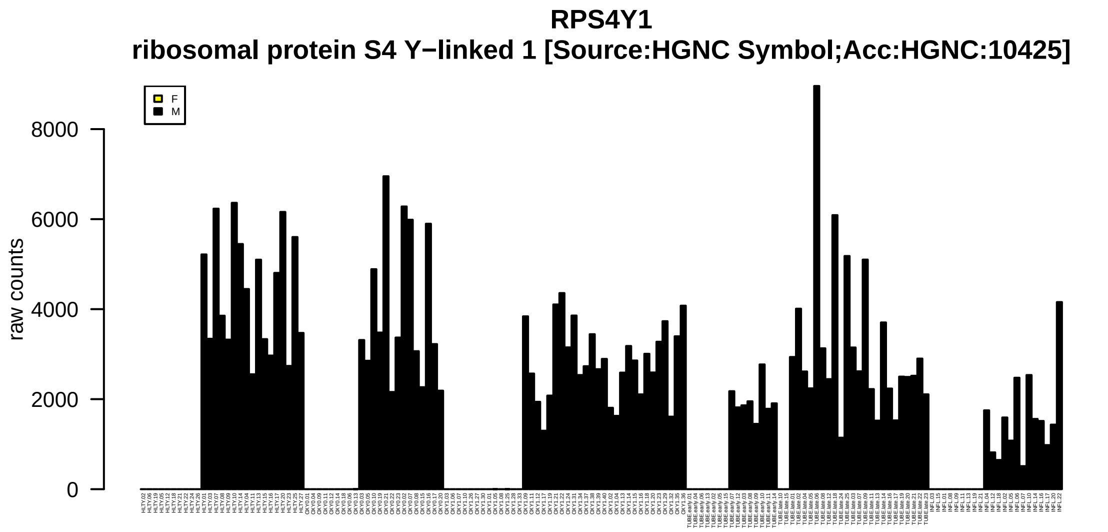
RPS4Y1
ribosomal protein S4 Y−linked 1 [Source:HGNC Symbol;Acc:HGNC:10425]
raw counts
0
2000
4000
6000
8000
F
M

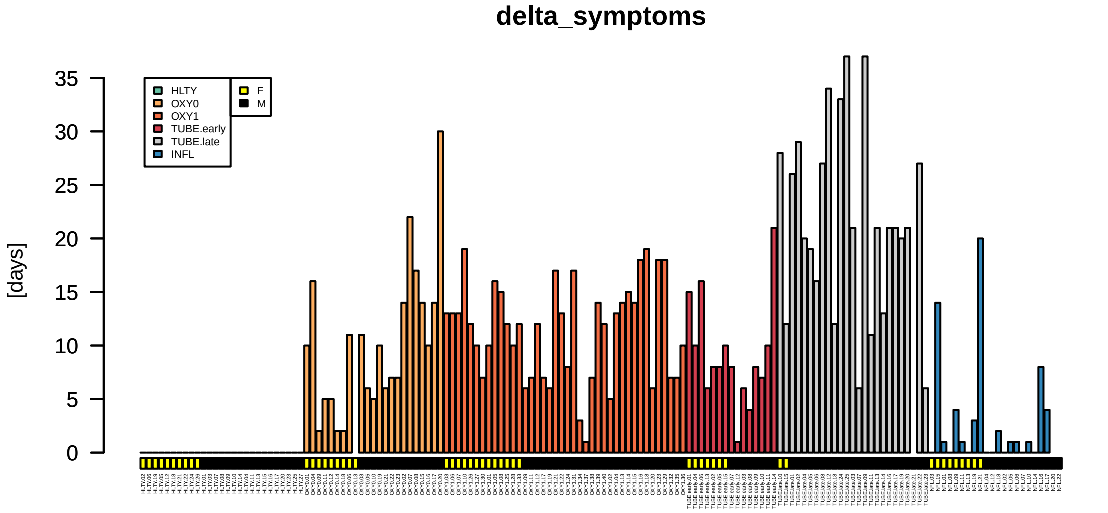
delta_symptoms
[days]
0
5
10
15
20
25
30
35
HLTY
OXY0
OXY1
TUBE.early
TUBE.late
INFL
F
M

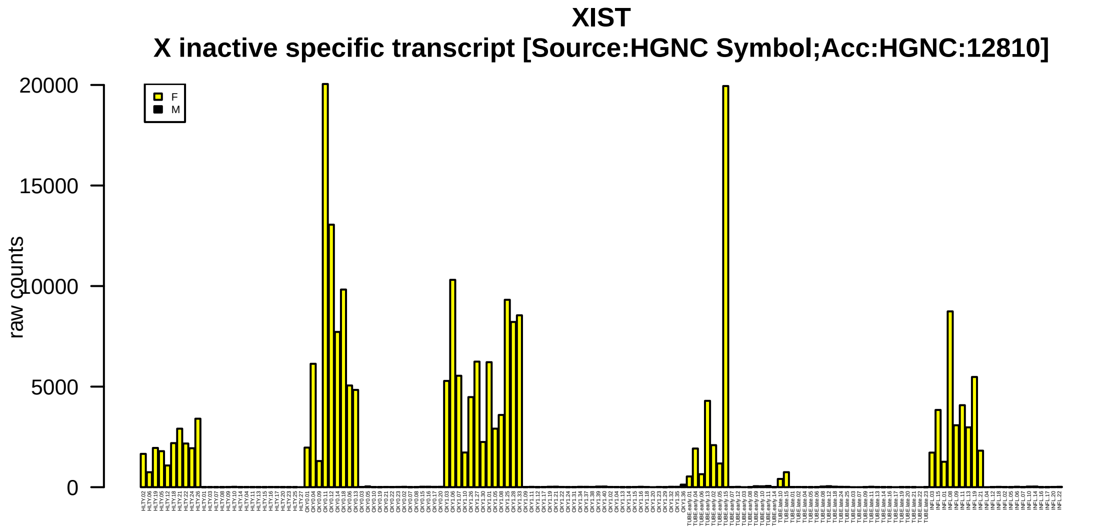
XIST
X inactive specific transcript [Source:HGNC Symbol;Acc:HGNC:12810]
raw counts
0
5000
10000
15000
20000
F
M
