# Supplementary material for: Transcriptomic Signature Differences Between SARS-CoV-2 and Influenza Virus Infected Patients
Source: Front Immunol. 2021 May 31;12:666163. doi: 10.3389/fimmu.2021.666163 (PMC8202013; doi:10.3389/fimmu.2021.666163)
Supplement: Supplementary Figure 2 — Unsupervised analysis: differentially expressed genes in KEGG pathways. MET stands for metabolism, GIP stands for Genetic Information Processing, EIP stands for Environmental Information Processing, CP stands for Cellular Processes, OS stands for Organismal Systems and HD stands for Human Diseases. [file Image_2.pdf]

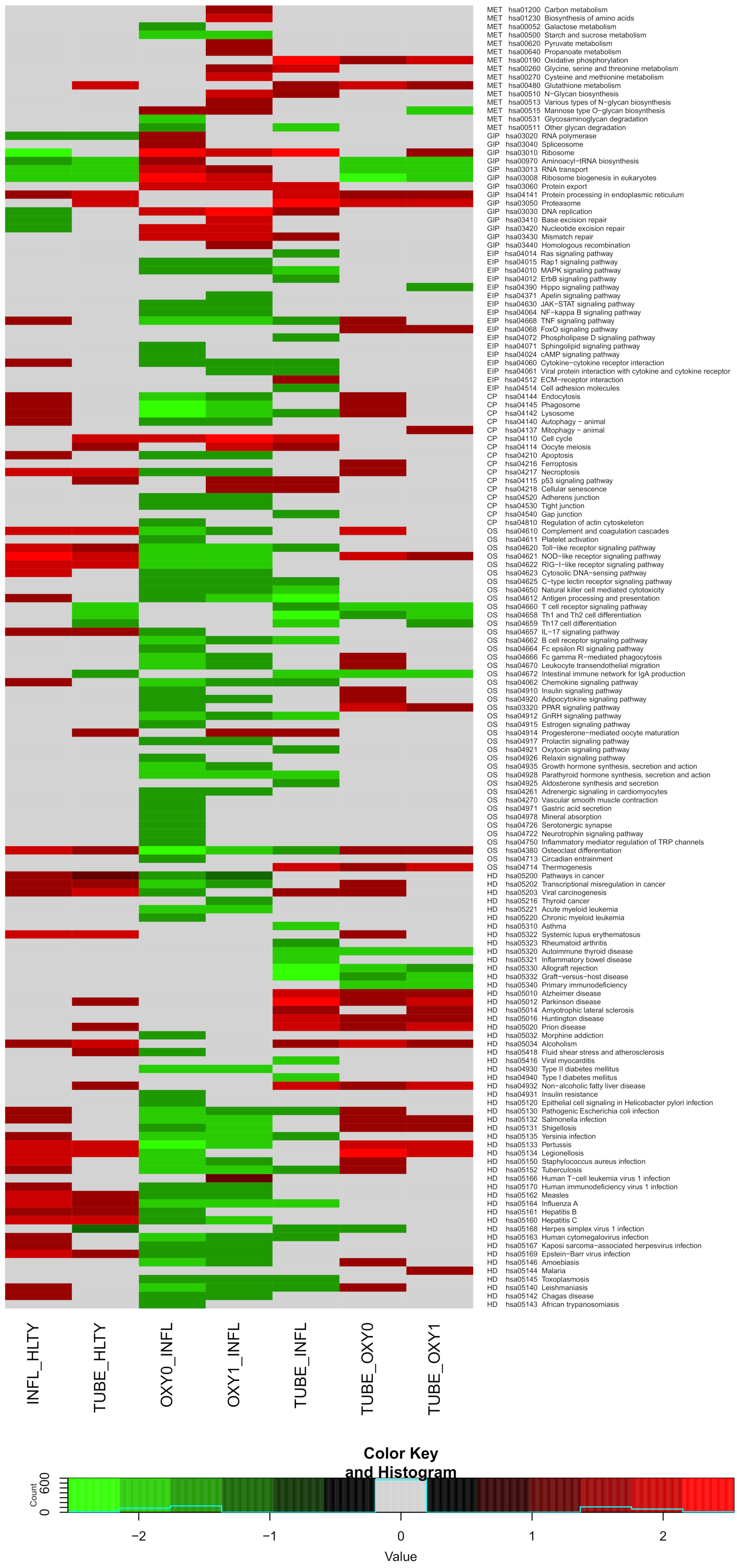
MET hsa01200 Carbon metabolism
MET hsa01230 Biosynthesis of amino acids
MET hsa00052 Galactose metabolism
MET hsa00500 Starch and sucrose metabolism
MET hsa00620 Pyruvate metabolism
MET hsa00640 Propanoate metabolism
MET hsa00190 Oxidative phosphorylation
MET hsa00260 Glycine, serine and threonine metabolism
MET hsa00270 Cysteine and methionine metabolism
MET hsa00480 Glutathione metabolism
MET hsa00510 N−Glycan biosynthesis
MET hsa00513 Various types of N−glycan biosynthesis
MET hsa00515 Mannose type O−glycan biosynthesis
MET hsa00531 Glycosaminoglycan degradation
MET hsa00511 Other glycan degradation
GIP hsa03020 RNA polymerase
GIP hsa03040 Spliceosome
GIP hsa03010 Ribosome
GIP hsa00970 Aminoacyl−tRNA biosynthesis
GIP hsa03013 RNA transport
GIP hsa03008 Ribosome biogenesis in eukaryotes
GIP hsa03060 Protein export
GIP hsa04141 Protein processing in endoplasmic reticulum
GIP hsa03050 Proteasome
GIP hsa03030 DNA replication
GIP hsa03410 Base excision repair
GIP hsa03420 Nucleotide excision repair
GIP hsa03430 Mismatch repair
GIP hsa03440 Homologous recombination
EIP hsa04014 Ras signaling pathway
EIP hsa04015 Rap1 signaling pathway
EIP hsa04010 MAPK signaling pathway
EIP hsa04012 ErbB signaling pathway
EIP hsa04390 Hippo signaling pathway
EIP hsa04371 Apelin signaling pathway
EIP hsa04630 JAK−STAT signaling pathway
EIP hsa04064 NF−kappa B signaling pathway
EIP hsa04668 TNF signaling pathway
EIP hsa04068 FoxO signaling pathway
EIP hsa04072 Phospholipase D signaling pathway
EIP hsa04071 Sphingolipid signaling pathway
EIP hsa04024 cAMP signaling pathway
EIP hsa04060 Cytokine−cytokine receptor interaction
EIP hsa04061 Viral protein interaction with cytokine and cytokine receptor
EIP hsa04512 ECM−receptor interaction
EIP hsa04514 Cell adhesion molecules
CP hsa04144 Endocytosis
CP hsa04145 Phagosome
CP hsa04142 Lysosome
CP hsa04140 Autophagy − animal
CP hsa04137 Mitophagy − animal
CP hsa04110 Cell cycle
CP hsa04114 Oocyte meiosis
CP hsa04210 Apoptosis
CP hsa04216 Ferroptosis
CP hsa04217 Necroptosis
CP hsa04115 p53 signaling pathway
CP hsa04218 Cellular senescence
CP hsa04520 Adherens junction
CP hsa04530 Tight junction
CP hsa04540 Gap junction
CP hsa04810 Regulation of actin cytoskeleton
OS hsa04610 Complement and coagulation cascades
OS hsa04611 Platelet activation
OS hsa04620 Toll−like receptor signaling pathway
OS hsa04621 NOD−like receptor signaling pathway
OS hsa04622 RIG−I−like receptor signaling pathway
OS hsa04623 Cytosolic DNA−sensing pathway
OS hsa04625 C−type lectin receptor signaling pathway
OS hsa04650 Natural killer cell mediated cytotoxicity
OS hsa04612 Antigen processing and presentation
OS hsa04660 T cell receptor signaling pathway
OS hsa04658 Th1 and Th2 cell differentiation
OS hsa04659 Th17 cell differentiation
OS hsa04657 IL−17 signaling pathway
OS hsa04662 B cell receptor signaling pathway
OS hsa04664 Fc epsilon RI signaling pathway
OS hsa04666 Fc gamma R−mediated phagocytosis
OS hsa04670 Leukocyte transendothelial migration
OS hsa04672 Intestinal immune network for IgA production
OS hsa04062 Chemokine signaling pathway
OS hsa04910 Insulin signaling pathway
OS hsa04920 Adipocytokine signaling pathway
OS hsa03320 PPAR signaling pathway
OS hsa04912 GnRH signaling pathway
OS hsa04915 Estrogen signaling pathway
OS hsa04914 Progesterone−mediated oocyte maturation
OS hsa04917 Prolactin signaling pathway
OS hsa04921 Oxytocin signaling pathway
OS hsa04926 Relaxin signaling pathway
OS hsa04935 Growth hormone synthesis, secretion and action
OS hsa04928 Parathyroid hormone synthesis, secretion and action
OS hsa04925 Aldosterone synthesis and secretion
OS hsa04261 Adrenergic signaling in cardiomyocytes
OS hsa04270 Vascular smooth muscle contraction
OS hsa04971 Gastric acid secretion
OS hsa04978 Mineral absorption
OS hsa04726 Serotonergic synapse
OS hsa04722 Neurotrophin signaling pathway
OS hsa04750 Inflammatory mediator regulation of TRP channels
OS hsa04380 Osteoclast differentiation
OS hsa04713 Circadian entrainment
OS hsa04714 Thermogenesis
HD hsa05200 Pathways in cancer
HD hsa05202 Transcriptional misregulation in cancer
HD hsa05203 Viral carcinogenesis
HD hsa05216 Thyroid cancer
HD hsa05221 Acute myeloid leukemia
HD hsa05220 Chronic myeloid leukemia
HD hsa05310 Asthma
HD hsa05322 Systemic lupus erythematosus
HD hsa05323 Rheumatoid arthritis
HD hsa05320 Autoimmune thyroid disease
HD hsa05321 Inflammatory bowel disease
HD hsa05330 Allograft rejection
HD hsa05332 Graft−versus−host disease
HD hsa05340 Primary immunodeficiency
HD hsa05010 Alzheimer disease
HD hsa05012 Parkinson disease
HD hsa05014 Amyotrophic lateral sclerosis
HD hsa05016 Huntington disease
HD hsa05020 Prion disease
HD hsa05032 Morphine addiction
HD hsa05034 Alcoholism
HD hsa05418 Fluid shear stress and atherosclerosis
HD hsa05416 Viral myocarditis
HD hsa04930 Type II diabetes mellitus
HD hsa04940 Type I diabetes mellitus
HD hsa04932 Non−alcoholic fatty liver disease
HD hsa04931 Insulin resistance
HD hsa05120 Epithelial cell signaling in Helicobacter pylori infection
HD hsa05130 Pathogenic Escherichia coli infection
HD hsa05132 Salmonella infection
HD hsa05131 Shigellosis
HD hsa05135 Yersinia infection
HD hsa05133 Pertussis
HD hsa05134 Legionellosis
HD hsa05150 Staphylococcus aureus infection
HD hsa05152 Tuberculosis
HD hsa05166 Human T−cell leukemia virus 1 infection
HD hsa05170 Human immunodeficiency virus 1 infection
HD hsa05162 Measles
HD hsa05164 Influenza A
HD hsa05161 Hepatitis B
HD hsa05160 Hepatitis C
HD hsa05168 Herpes simplex virus 1 infection
HD hsa05163 Human cytomegalovirus infection
HD hsa05167 Kaposi sarcoma−associated herpesvirus infection
HD hsa05169 Epstein−Barr virus infection
HD hsa05146 Amoebiasis
HD hsa05144 Malaria
HD hsa05145 Toxoplasmosis
HD hsa05140 Leishmaniasis
HD hsa05142 Chagas disease
HD hsa05143 African trypanosomiasis
INFL_HLTY
TUBE_HLTY
OXY0_INFL
OXY1_INFL
TUBE_INFL
TUBE_OXY0
TUBE_OXY1
Color Key
and Histogram
Count
0
600
−2
−1
0
1
2
Value
