# Supplementary figures and images for: Transcriptomic Signature Differences Between SARS-CoV-2 and Influenza Virus Infected Patients
Source: Front Immunol. 2021 May 31;12:666163. doi: 10.3389/fimmu.2021.666163 (PMC8202013; doi:10.3389/fimmu.2021.666163)

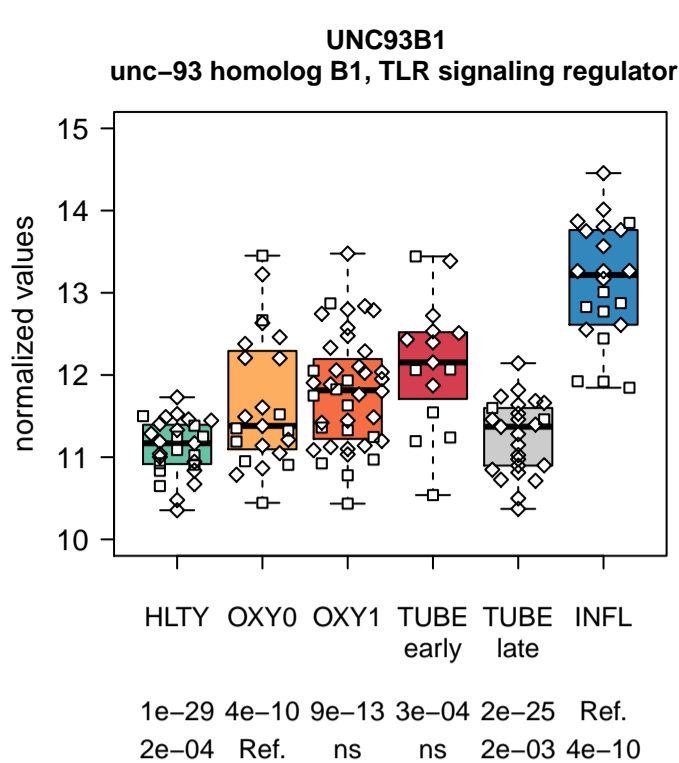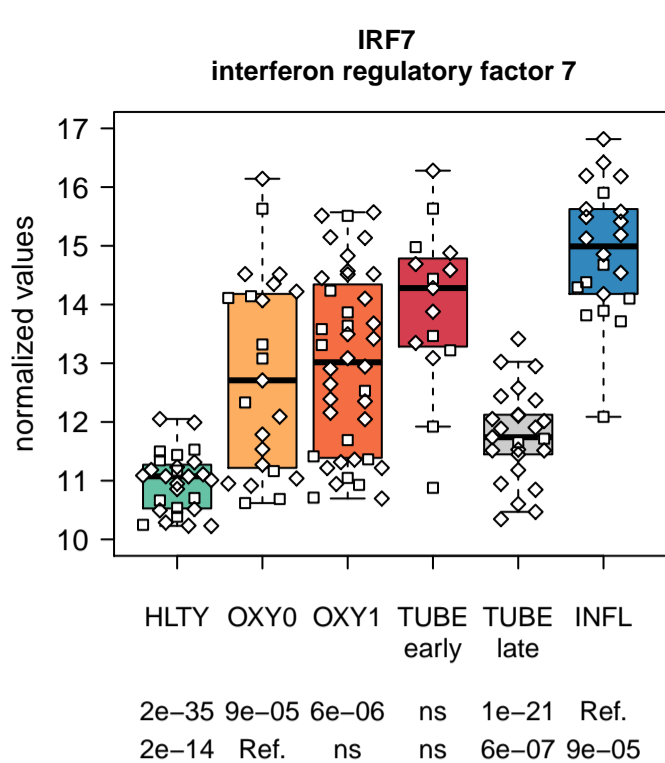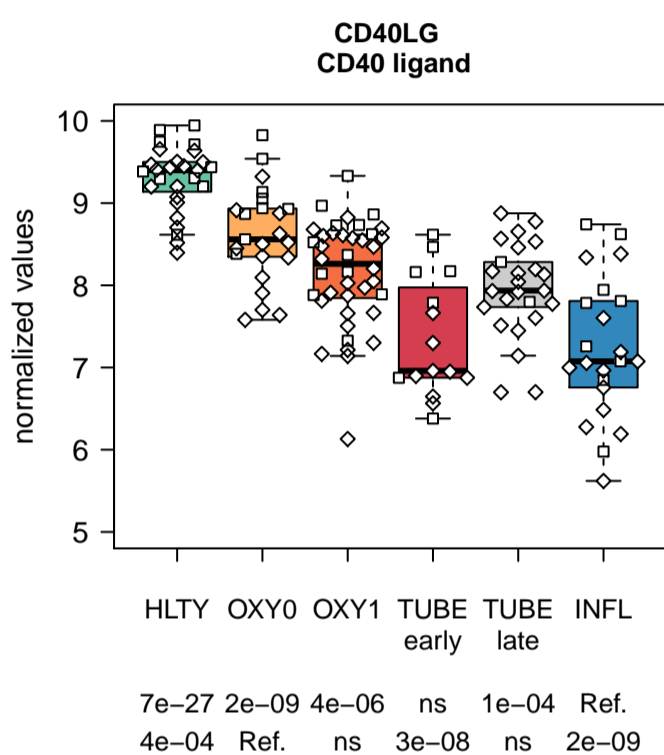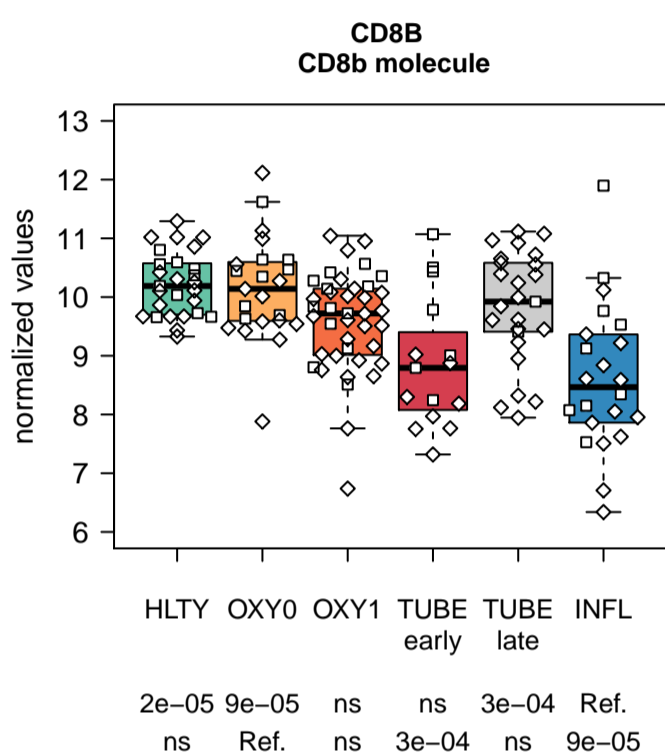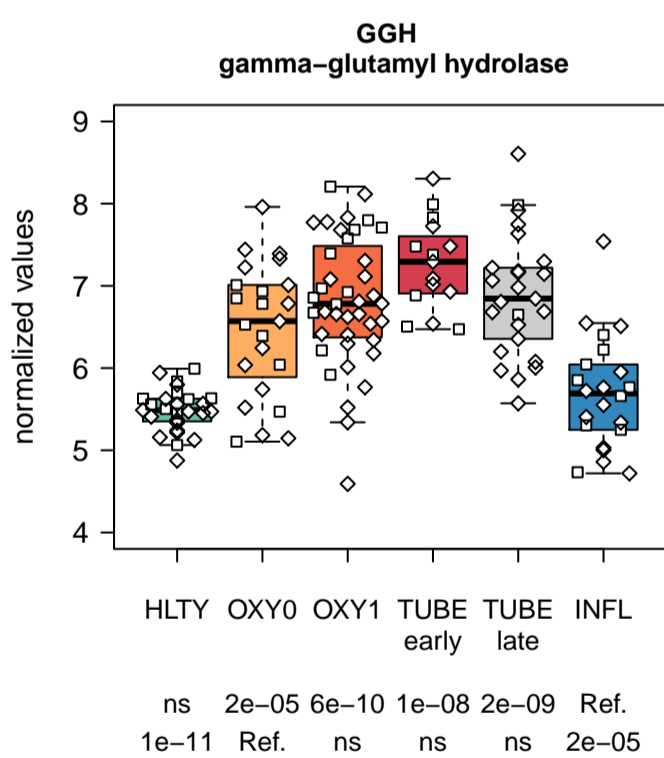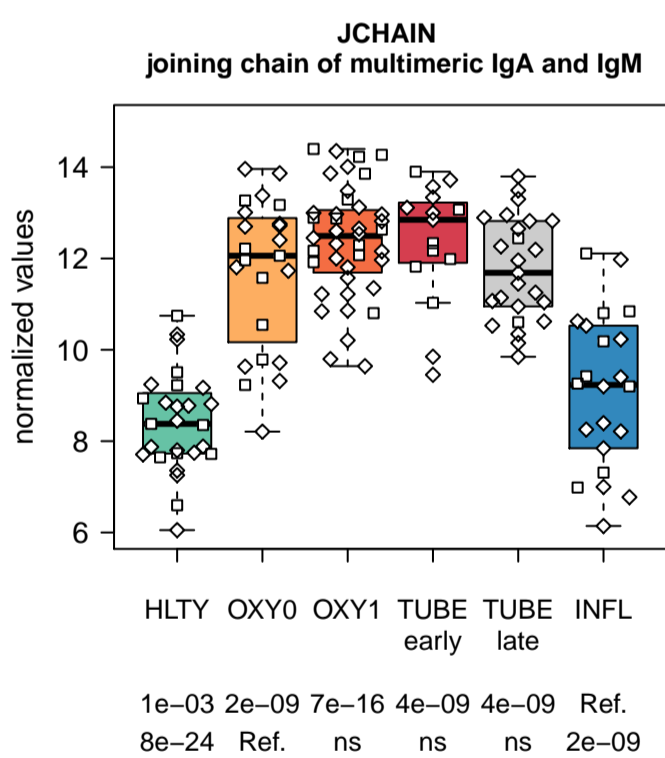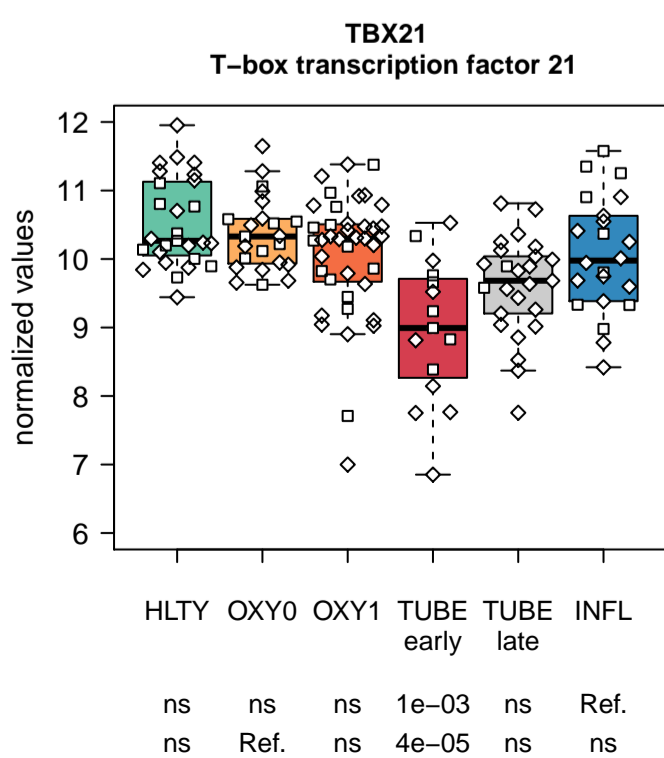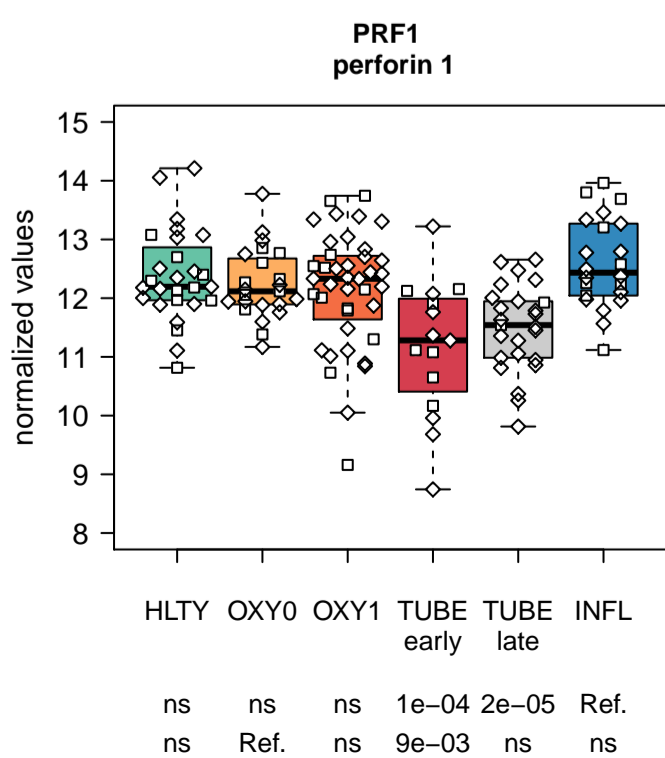

Supplement: Supplementary Figure 3 — Interpretation of boxplots (“ascending”, “descending”, “hill” and “valley” pattern). Expression patterns across the different groups were regarded as “ascending” or “descending” when median expression levels progressively increased, or decreased, respectively, from HLTY to OXY0, OXY1, TUBE-early, and INFL. Conversely, patterns were qualified to have a “hill” or “valley” pattern, when peak and lowest median expression levels were seen in COVID-19 (OXY1 or TUBE-early), and the lowest and higher in both HLTY and INFL, respectively. Because TUBE-late often represented a convalescent status, their gene expression levels were ignored in this pattern qualification. [file Image_3.pdf]

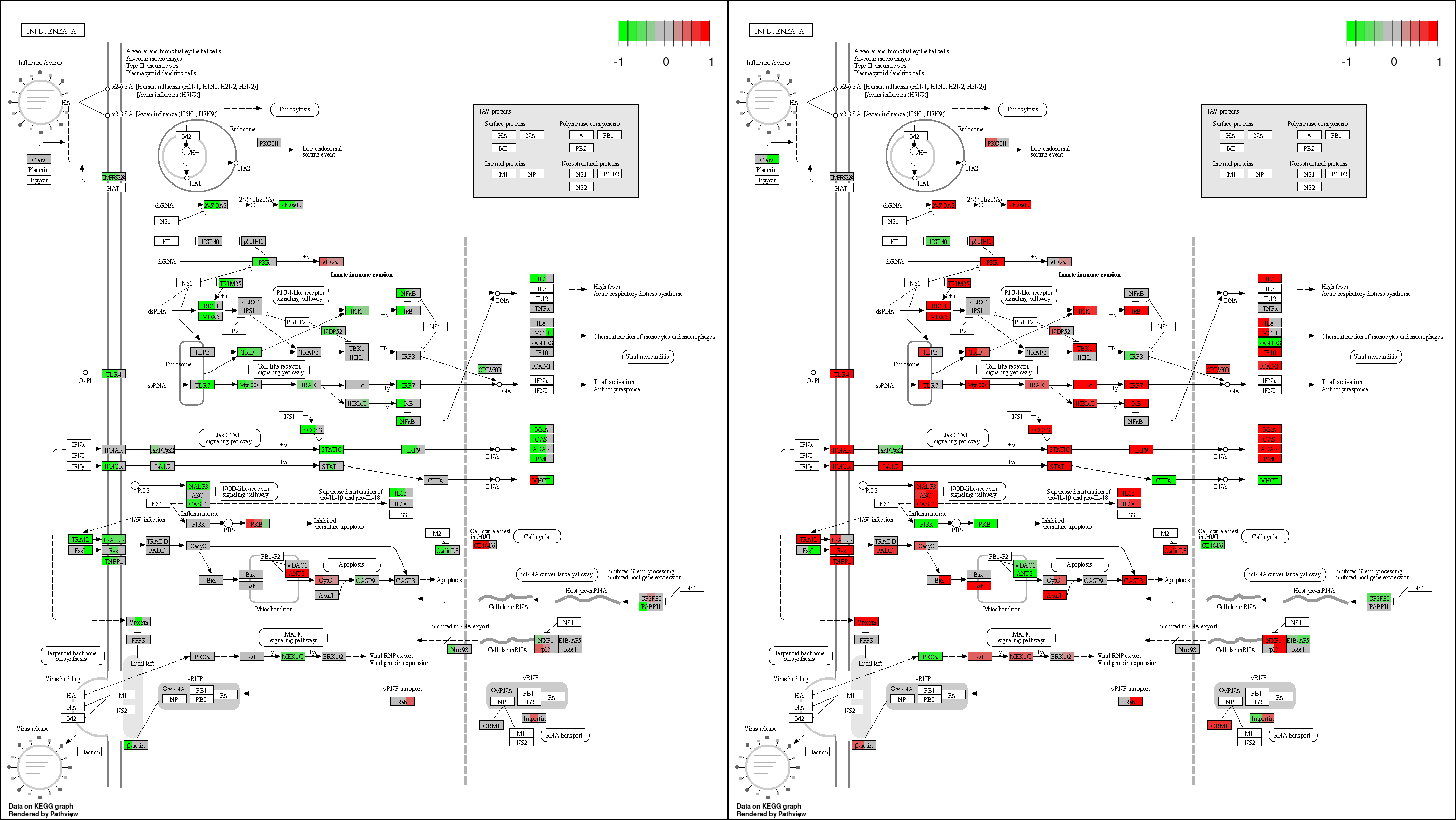

Supplement: Supplementary Figure 4 — KEGG map colored with pathview (117) “Influenza A” pathway (hsa05164). Pathways involved in the detection of Influenza A overlap with those involved in the detection of other RNA viruses including SARS-CoV-2. Significantly DEGs (adj. P-Value < 0.01) are colored based on their log2 fold change. Left panel: boxes are separated into 3 slots, for OXY0 versus INFL, OXY1 versus INFL and TUBE-early versus INFL. Right panel: boxes are separated into 2 slots, for INFL versus HTLY and TUBE_early versus HLTY. [file Image_4.png]

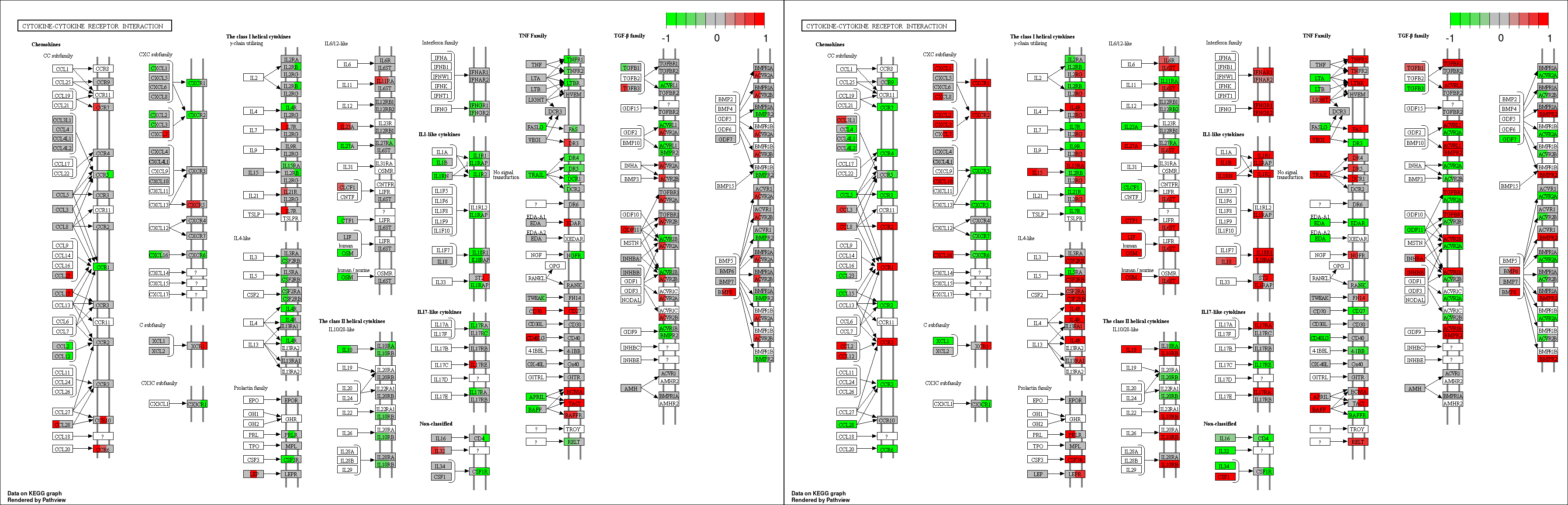

Supplement: Supplementary Figure 5 — KEGG map colored with pathview (117) “Cytokines-cytokine receptor interaction” (hsa04060). Significantly DEGs (adj. P-Value < 0.01) are colored based on their log2 fold change. Left panel: boxes are separated into 3 slots, for OXY0 versus INFL, OXY1 versus INFL and TUBE-early versus INFL. Right panel: boxes are separated into 2 slots, for INFL versus HTLY and TUBE_early versus HLTY. [file Image_5.png]

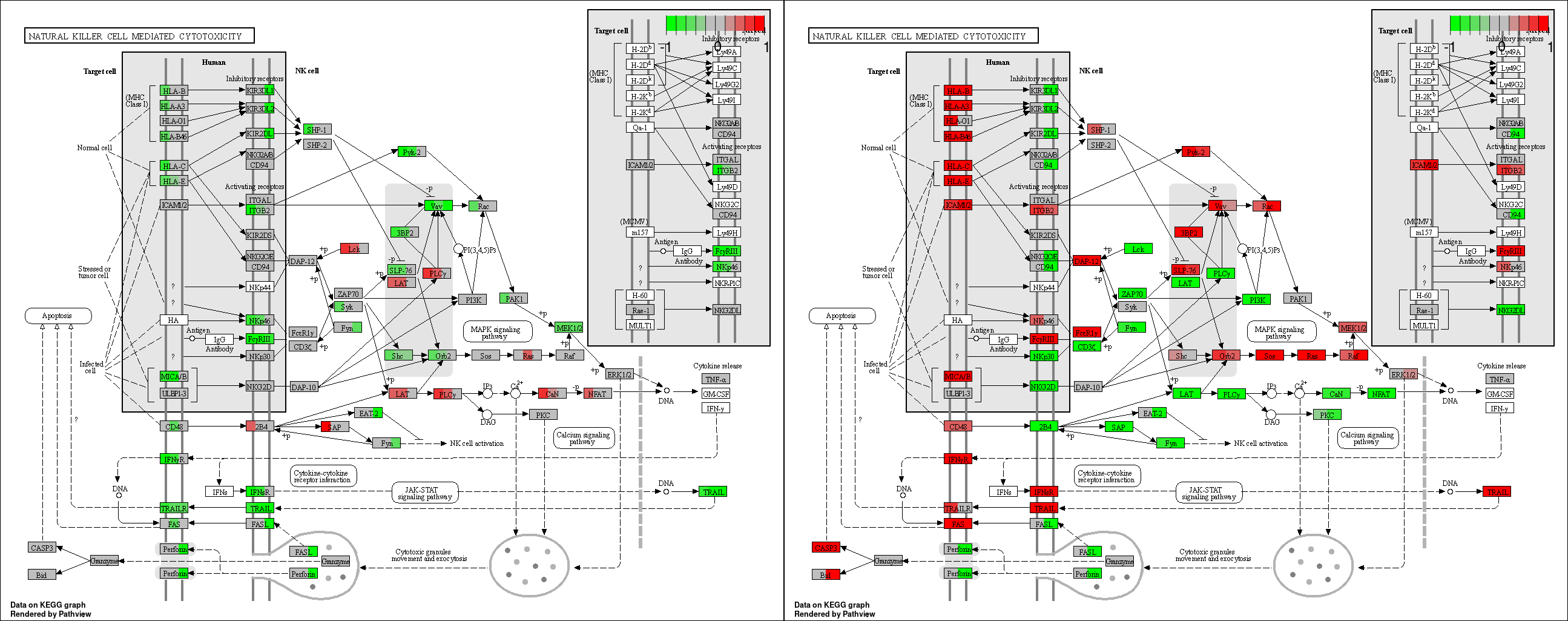

Supplement: Supplementary Figure 6 — KEGG map colored with pathview (117) “Natural killer cell mediated toxicity” (hsa04650). Significantly DEGs (adj. P-Value < 0.01) are colored based on their log2 fold change. Left panel: boxes are separated into 3 slots, for OXY0 versus INFL, OXY1 versus INFL and TUBE-early versus INFL. Right panel: boxes are separated into 2 slots, for INFL versus HTLY and TUBE_early versus HLTY. [file Image_6.png]

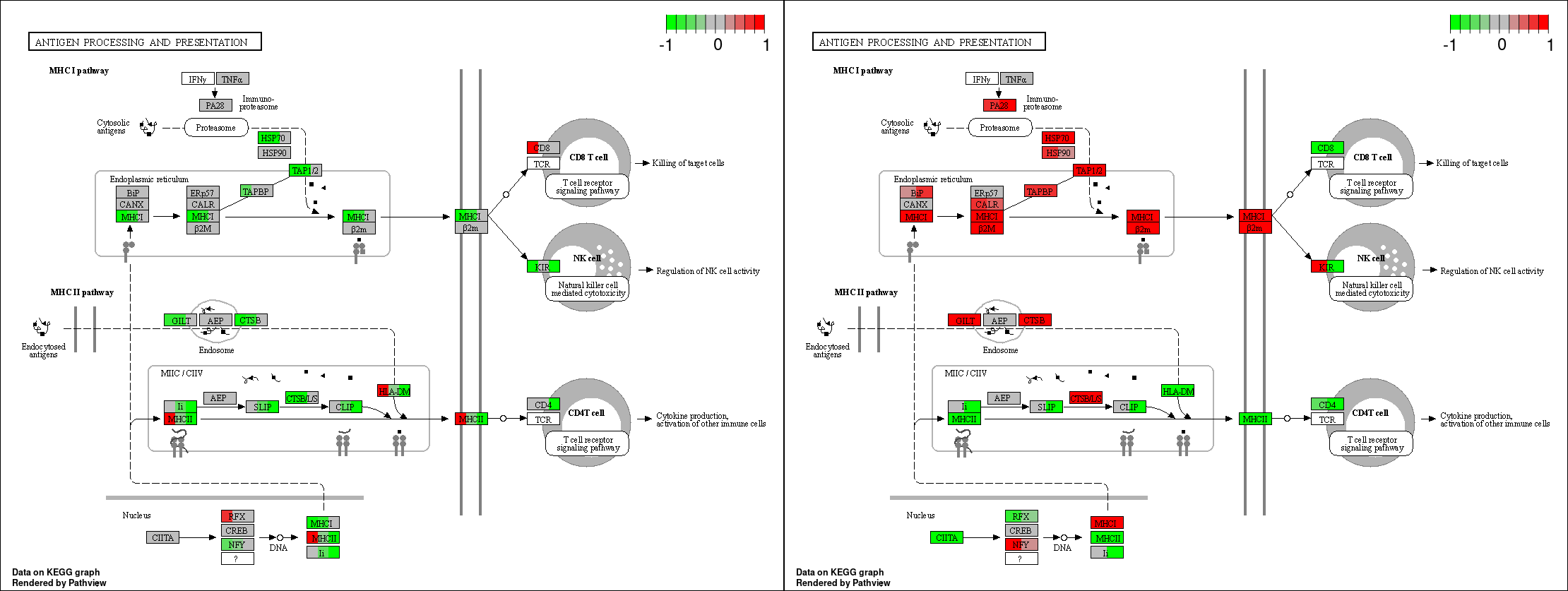

Supplement: Supplementary Figure 8 — Box plots for selected genes involved in different T cells functions. [file Image_8.png]

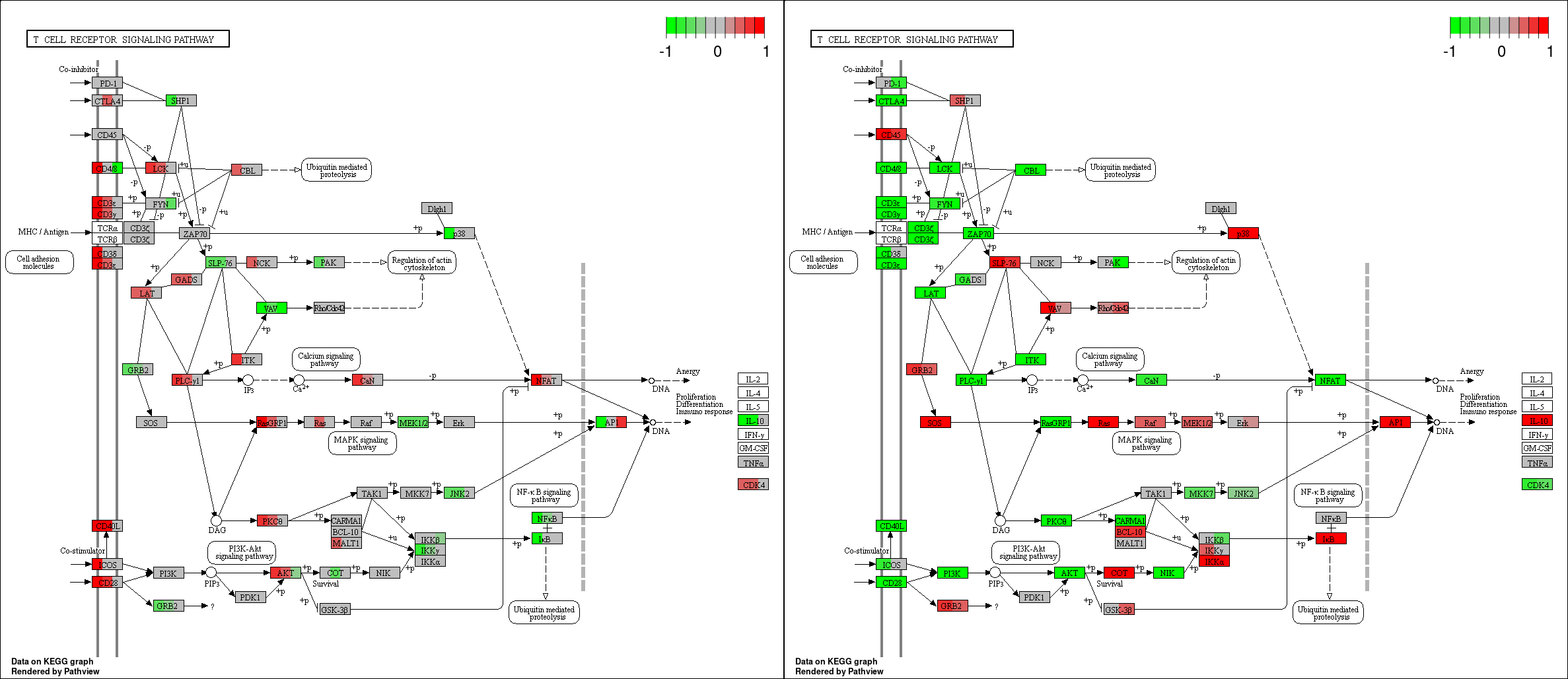

Supplement: Supplementary Figure 9 — KEGG map colored with pathview (117) “T cell receptor signaling pathway” (hsa04660). Significantly DEGs (adj. P-Value < 0.01) are colored based on their log2 fold change. Left panel: boxes are separated into 3 slots, for OXY0 versus INFL, OXY1 versus INFL and TUBE-early versus INFL. Right panel: boxes are separated into 2 slots, for INFL versus HTLY and TUBE_early versus HLTY. [file Image_9.png]

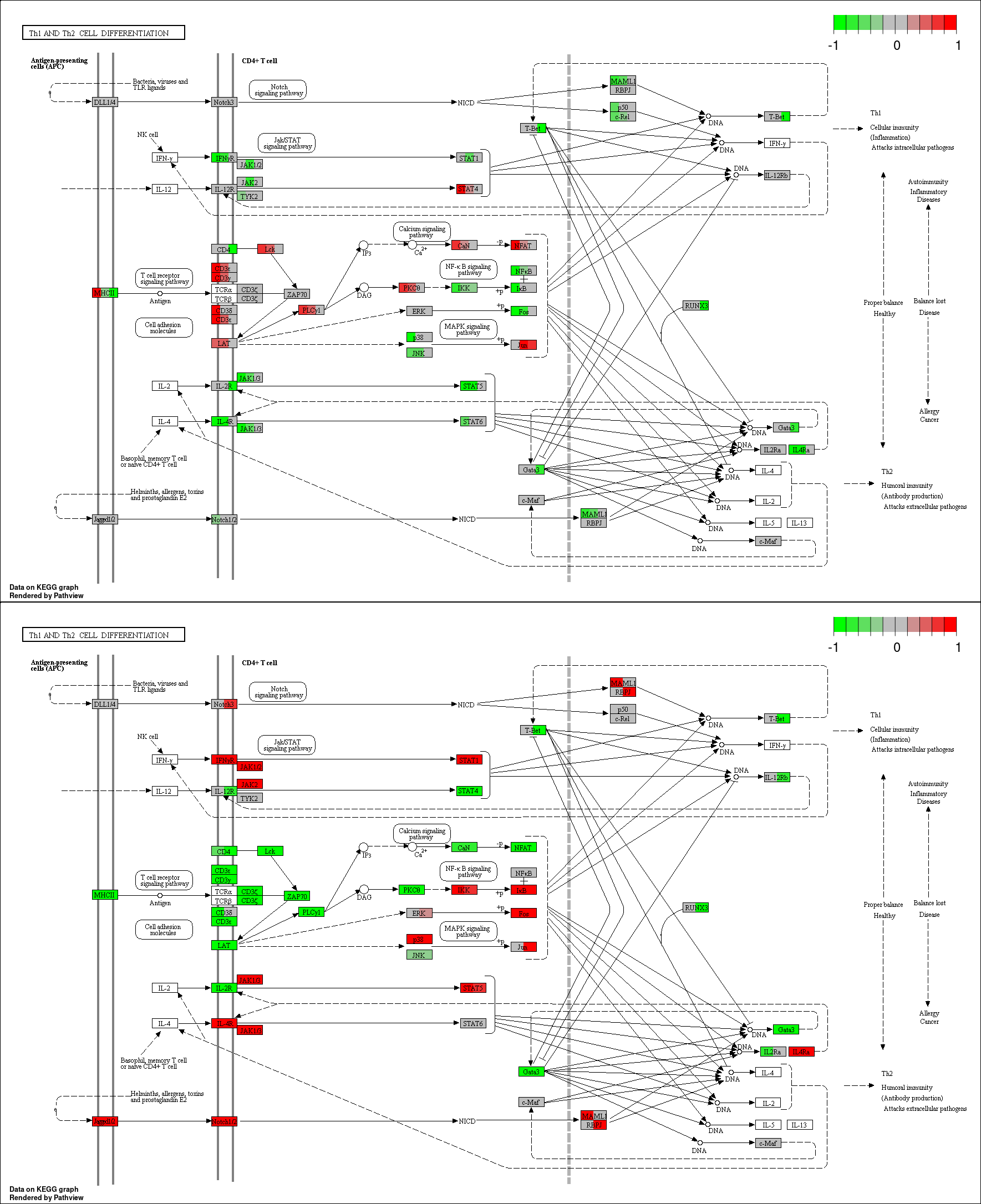

Supplement: Supplementary Figure 10 — KEGG map colored with pathview (117) “Th1 and Th2 differentiation” (hsa04658). Significantly DEGs (adj. P-Value < 0.01) are colored based on their log2 fold change. Upper panel: boxes are separated into 3 slots, for OXY0 versus INFL, OXY1 versus INFL and TUBE-early versus INFL. Lower panel: boxes are separated into 2 slots, for INFL versus HTLY and TUBE_early versus HLTY. [file Image_10.png]

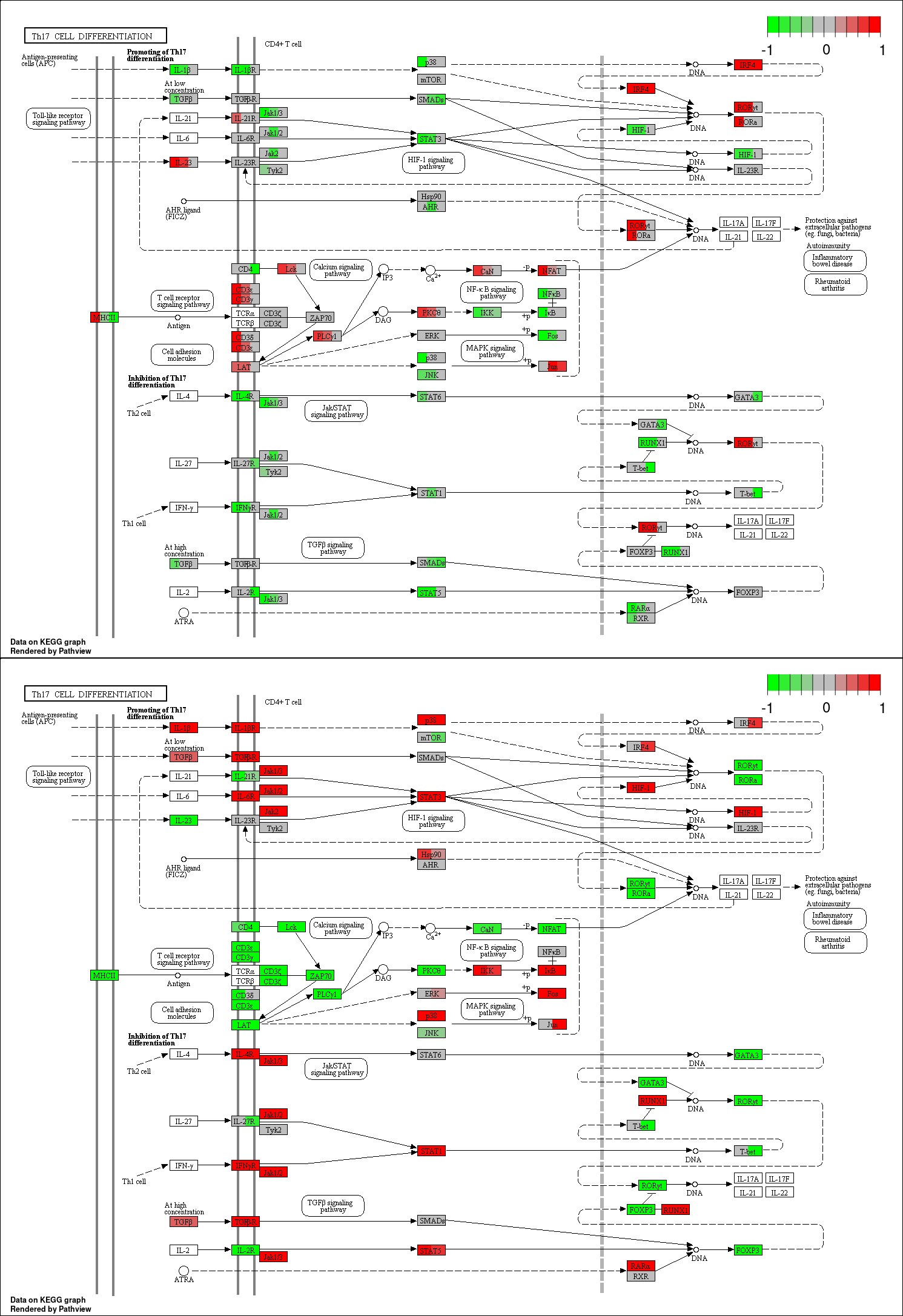

Supplement: Supplementary Figure 11 — KEGG map colored with pathview (117) “Th17 differentiation” (hsa04659). Significantly DEGs (adj. P-Value < 0.01) are colored based on their log2 fold change. Upper panel: boxes are separated into 3 slots, for OXY0 versus INFL, OXY1 versus INFL and TUBE-early versus INFL. Lower panel: boxes are separated into 2 slots, for INFL versus HTLY and TUBE_early versus HLTY. [file Image_11.png]

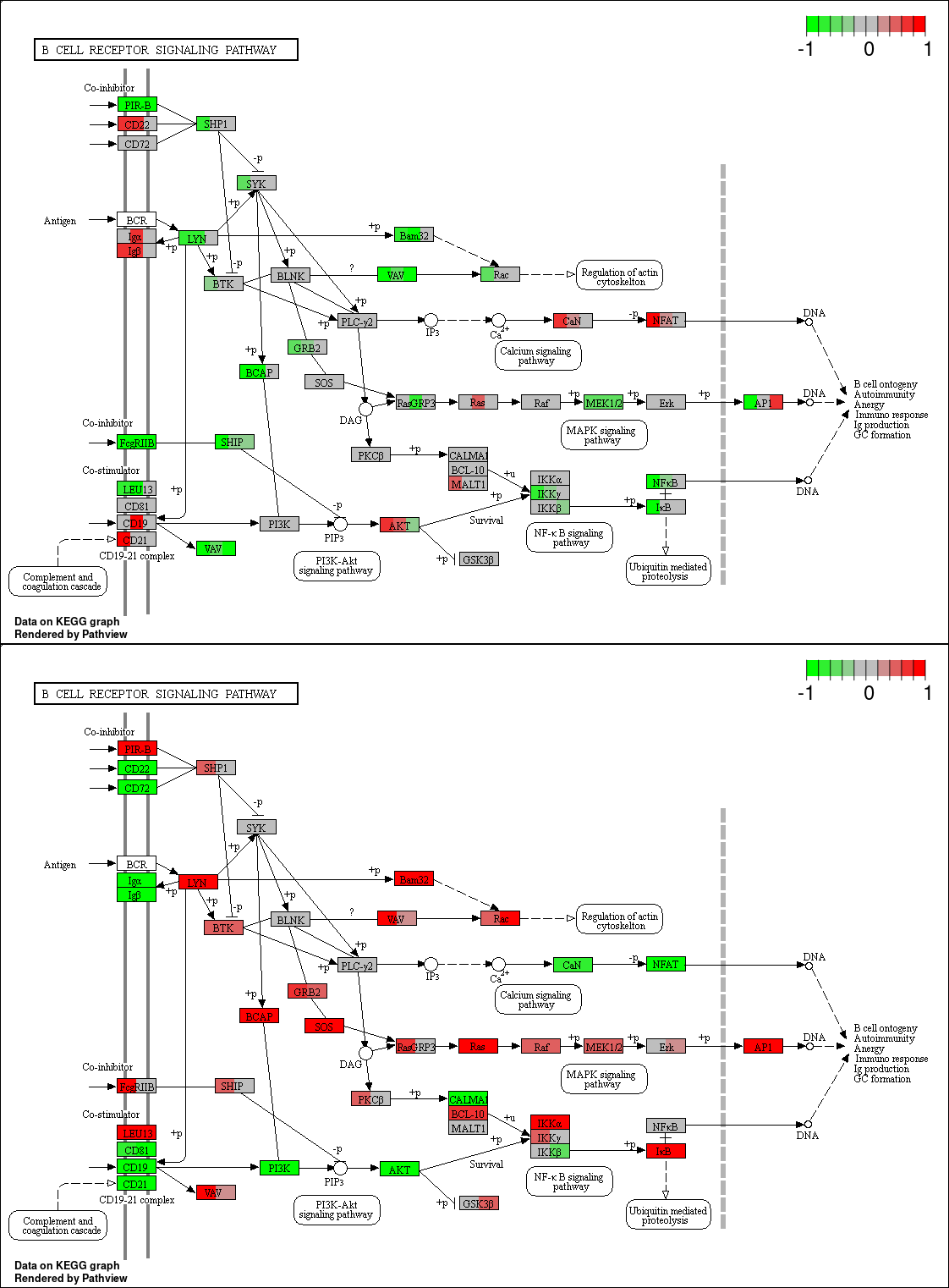

Supplement: Supplementary Figure 12 — KEGG map colored with pathview (117) "B cell receptor signaling pathway" (hsa04662). Significantly DEGs (adj. P-Value < 0.01) are colored based on their log2 fold change. Upper panel: boxes are separated into 3 slots, for OXY0 versus INFL, OXY1 versus INFL and TUBE-early versus INFL. Lower panel: boxes are separated into 2 slots, for INFL versus HTLY and TUBE_early versus HLTY. [file Image_12.png]

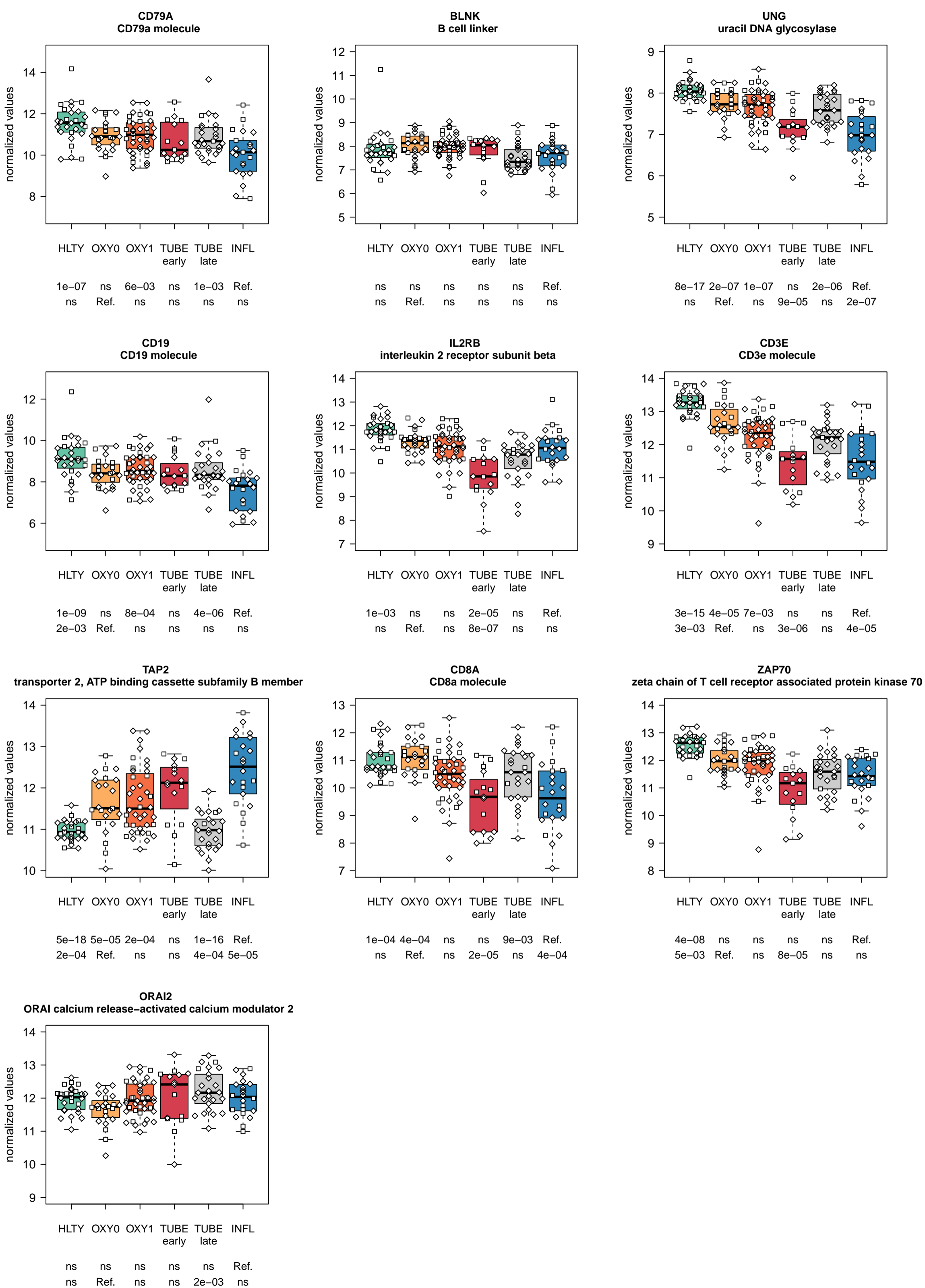

Supplement: Supplementary Figure 13 — Box plots for selected genes involved in B cells functions/maturation/regulation. [file Image_13.pdf]

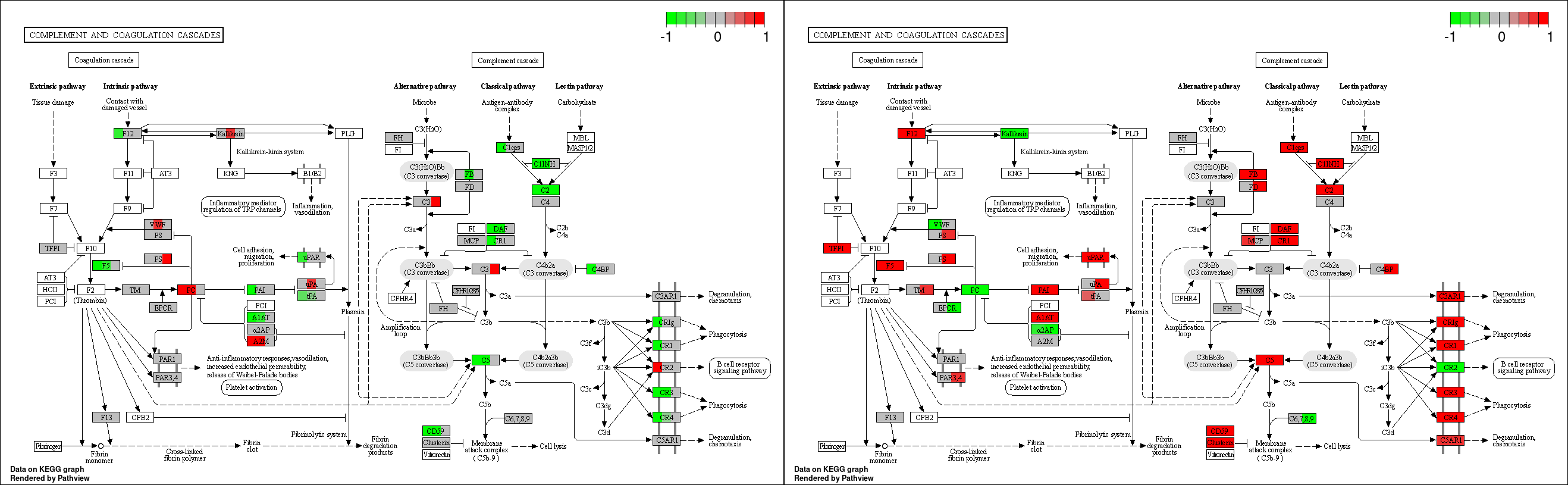

Supplement: Supplementary Figure 14 — KEGG map colored with pathview (117) "Complement and coagulation cascades" (hsa04659). Significantly DEGs (adj. P-Value < 0.01) are colored based on their log2 fold change. Left panel: boxes are separated into 3 slots, for OXY0 versus INFL, OXY1 versus INFL and TUBE-early versus INFL. Right panel: boxes are separated into 2 slots, for INFL versus HTLY and TUBE_early versus HLTY. [file Image_14.png]

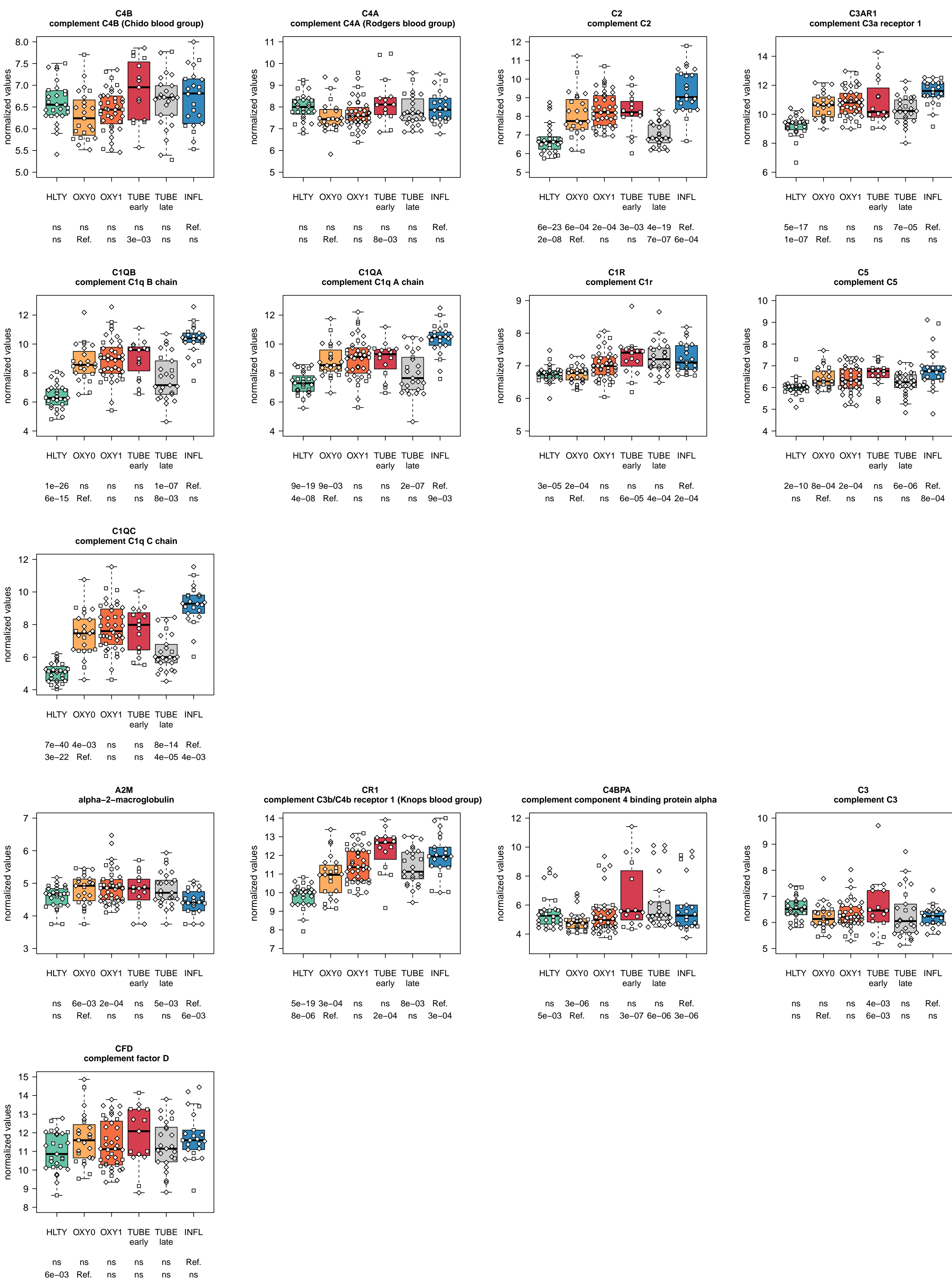

Supplement: Supplementary Figure 15 — Boxplots plots for complement, coagulation and blood groups. [file Image_15.pdf]
