# Supplementary material for: Transcriptomic Signature Differences Between SARS-CoV-2 and Influenza Virus Infected Patients
Source: Front Immunol. 2021 May 31;12:666163. doi: 10.3389/fimmu.2021.666163 (PMC8202013; doi:10.3389/fimmu.2021.666163)
Supplement: Supplementary Figure 7 — KEGG map colored with pathview (117) “Antigen processing and presentation” (hsa04612). Significantly DEGs (adj. P-Value < 0.01) are colored based on their log2 fold change. Left panel: boxes are separated into 3 slots, for OXY0 versus INFL, OXY1 versus INFL and TUBE-early versus INFL. Right panel: boxes are separated into 2 slots, for INFL versus HTLY and TUBE_early versus HLTY. [file Image_7.pdf]

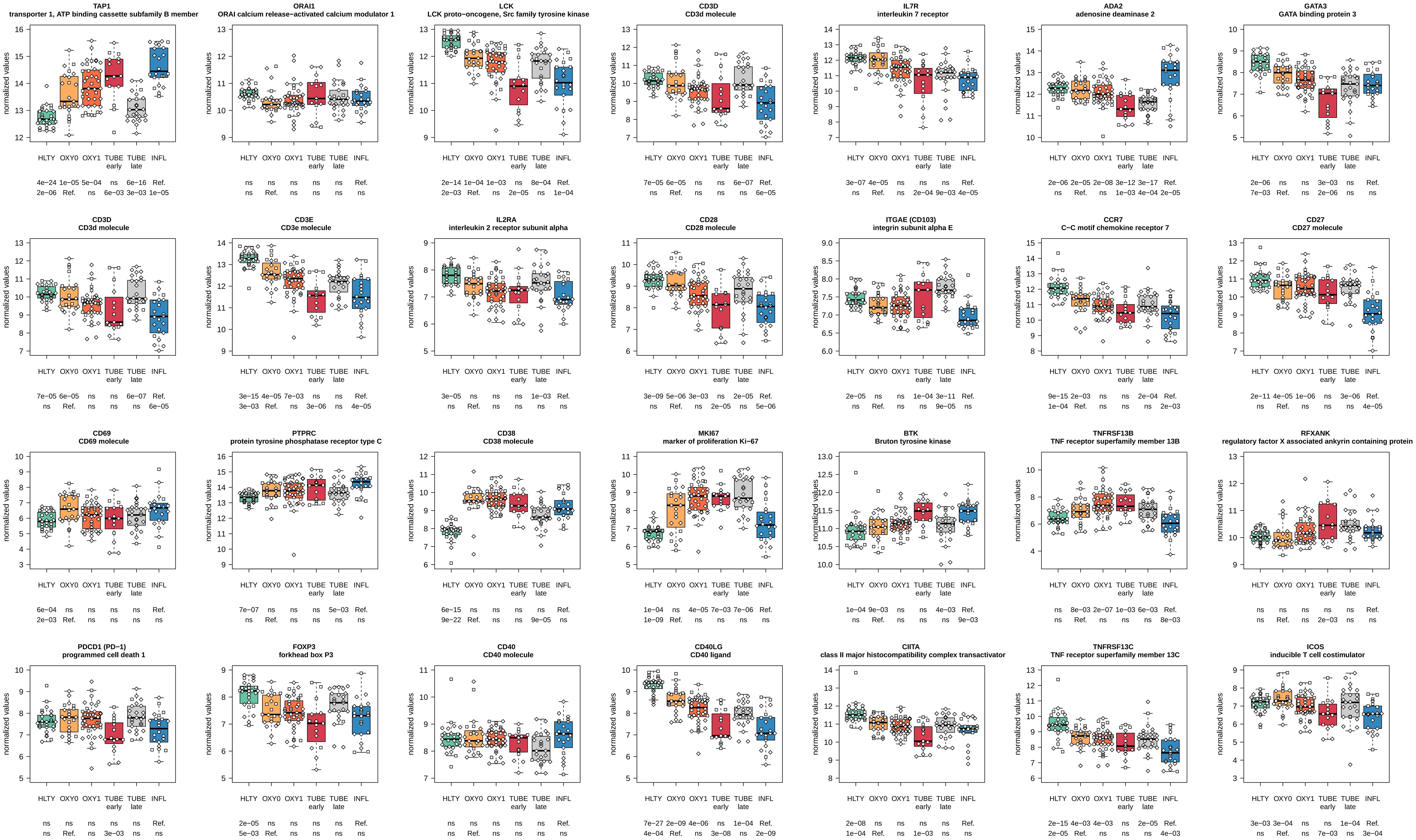
TAP1
transporter 1, ATP binding cassette subfamily B member
normalized values
HLTY OXY0 OXY1 TUBE early TUBE late INFL
4e−24 1e−05 5e−04 ns 6e−16 Ref.
2e−06 Ref. ns 6e−03 3e−03 1e−05
ORAI1
ORAI calcium release−activated calcium modulator 1
normalized values
HLTY OXY0 OXY1 TUBE early TUBE late INFL
ns ns ns ns ns Ref.
ns Ref. ns ns ns ns
LCK
LCK proto−oncogene, Src family tyrosine kinase
normalized values
HLTY OXY0 OXY1 TUBE early TUBE late INFL
2e−14 1e−04 1e−03 ns 8e−04 Ref.
2e−03 Ref. ns 2e−05 ns 1e−04
CD3D
CD3d molecule
normalized values
HLTY OXY0 OXY1 TUBE early TUBE late INFL
7e−05 6e−05 ns ns 6e−07 Ref.
ns Ref. ns ns ns 6e−05
IL7R
interleukin 7 receptor
normalized values
HLTY OXY0 OXY1 TUBE early TUBE late INFL
3e−07 4e−05 ns ns ns Ref.
ns Ref. ns 2e−04 9e−03 4e−05
ADA2
adenosine deaminase 2
normalized values
HLTY OXY0 OXY1 TUBE early TUBE late INFL
2e−06 2e−05 2e−08 3e−12 3e−17 Ref.
ns Ref. ns 1e−03 4e−04 2e−05
GATA3
GATA binding protein 3
normalized values
HLTY OXY0 OXY1 TUBE early TUBE late INFL
2e−06 ns ns 3e−03 ns Ref.
7e−03 Ref. ns 2e−06 ns ns
CD3D
CD3d molecule
normalized values
HLTY OXY0 OXY1 TUBE early TUBE late INFL
7e−05 6e−05 ns ns 6e−07 Ref.
ns Ref. ns ns ns 6e−05
CD3E
CD3e molecule
normalized values
HLTY OXY0 OXY1 TUBE early TUBE late INFL
3e−15 4e−05 7e−03 ns ns Ref.
3e−03 Ref. ns 3e−06 ns 4e−05
IL2RA
interleukin 2 receptor subunit alpha
normalized values
HLTY OXY0 OXY1 TUBE early TUBE late INFL
3e−05 ns ns ns 1e−03 Ref.
ns Ref. ns ns ns ns
CD28
CD28 molecule
normalized values
HLTY OXY0 OXY1 TUBE early TUBE late INFL
3e−09 5e−06 3e−03 ns 2e−05 Ref.
ns Ref. ns 2e−05 ns 5e−06
ITGAE (CD103)
integrin subunit alpha E
normalized values
HLTY OXY0 OXY1 TUBE early TUBE late INFL
2e−05 ns ns 1e−04 3e−11 Ref.
ns Ref. ns ns 9e−05 ns
CCR7
C−C motif chemokine receptor 7
normalized values
HLTY OXY0 OXY1 TUBE early TUBE late INFL
9e−15 2e−03 ns ns 2e−04 Ref.
1e−04 Ref. ns ns ns 2e−03
CD27
CD27 molecule
normalized values
HLTY OXY0 OXY1 TUBE early TUBE late INFL
2e−11 4e−05 1e−06 ns 3e−06 Ref.
ns Ref. ns ns ns 4e−05
CD69
CD69 molecule
normalized values
HLTY OXY0 OXY1 TUBE early TUBE late INFL
6e−04 ns ns ns ns Ref.
2e−03 Ref. ns ns ns ns
PTPRC
protein tyrosine phosphatase receptor type C
normalized values
HLTY OXY0 OXY1 TUBE early TUBE late INFL
7e−07 ns ns ns 5e−03 Ref.
ns Ref. ns ns ns ns
CD38
CD38 molecule
normalized values
HLTY OXY0 OXY1 TUBE early TUBE late INFL
6e−15 ns ns ns ns Ref.
9e−22 Ref. ns ns 9e−05 ns
MKI67
marker of proliferation Ki−67
normalized values
HLTY OXY0 OXY1 TUBE early TUBE late INFL
1e−04 ns 4e−05 7e−03 7e−06 Ref.
1e−09 Ref. ns ns ns ns
BTK
Bruton tyrosine kinase
normalized values
HLTY OXY0 OXY1 TUBE early TUBE late INFL
1e−04 9e−03 ns ns 4e−03 Ref.
ns Ref. ns ns ns 9e−03
TNFRSF13B
TNF receptor superfamily member 13B
normalized values
HLTY OXY0 OXY1 TUBE early TUBE late INFL
ns 8e−03 2e−07 1e−03 6e−03 Ref.
ns Ref. ns ns ns 8e−03
RFXANK
regulatory factor X associated ankyrin containing protein
normalized values
HLTY OXY0 OXY1 TUBE early TUBE late INFL
ns ns ns ns ns Ref.
ns Ref. ns 2e−03 ns ns
PDCD1 (PD−1)
programmed cell death 1
normalized values
HLTY OXY0 OXY1 TUBE early TUBE late INFL
ns ns ns ns ns Ref.
ns Ref. ns 3e−03 ns ns
FOXP3
forkhead box P3
normalized values
HLTY OXY0 OXY1 TUBE early TUBE late INFL
2e−05 ns ns ns ns Ref.
5e−03 Ref. ns ns ns ns
CD40
CD40 molecule
normalized values
HLTY OXY0 OXY1 TUBE early TUBE late INFL
ns ns ns ns ns Ref.
ns Ref. ns ns ns ns
CD40LG
CD40 ligand
normalized values
HLTY OXY0 OXY1 TUBE early TUBE late INFL
7e−27 2e−09 4e−06 ns 1e−04 Ref.
4e−04 Ref. ns 3e−08 ns 2e−09
CIITA
class II major histocompatibility complex transactivator
normalized values
HLTY OXY0 OXY1 TUBE early TUBE late INFL
2e−08 ns ns ns ns Ref.
1e−04 Ref. ns 1e−03 ns ns
TNFRSF13C
TNF receptor superfamily member 13C
normalized values
HLTY OXY0 OXY1 TUBE early TUBE late INFL
2e−15 4e−03 4e−03 ns 2e−05 Ref.
2e−05 Ref. ns ns ns 4e−03
ICOS
inducible T cell costimulator
normalized values
HLTY OXY0 OXY1 TUBE early TUBE late INFL
3e−03 3e−04 ns ns 1e−04 Ref.
ns Ref. ns 7e−03 ns 3e−04
